# CHARIOT: a phase I study of berzosertib with chemoradiotherapy in oesophageal and other solid cancers using time to event continual reassessment method

**DOI:** 10.1038/s41416-023-02542-1

**Published:** 2023-12-21

**Authors:** S. R. Javed, S. Lord, S. El Badri, R. Harman, J. Holmes, F. Kamzi, T. Maughan, D. McIntosh, S. Mukherjee, A. Ooms, G. Radhakrishna, P. Shaw, M. A. Hawkins

**Affiliations:** 1 Department of Oncology, University of Oxford, Oxford, UK; 2 Primary Care Clinical Trials Unit, Nuffield Department of Primary Care Health Sciences, University of Oxford, Oxford, UK; 3 Norfolk and Norwich University Hospitals NHS Foundation Trust, Norwich, UK; 4 Beatson West of Scotland Cancer Centre, Glasgow, UK; 5 Oxford University Hospitals NHS Foundation Trust, Oxford, UK; 6 Oxford Clinical Trials Research Unit, Centre for Statistics in Medicine, Nuffield Department of Orthopaedics, Rheumatology & Musculoskeletal Sciences, University of Oxford, Oxford, UK; 7 The Christie NHS Foundation Trust, Manchester, UK; 8 Velindre University NHS Trust, Cardiff, UK; 9 UCL Medical Physics and Biomedical Engineering, University College London, London, UK

**Keywords:** Drug development, Cancer

## Abstract

**Background:**

Berzosertib (M6620) is a highly potent (IC50  =  19 nM) and selective, first-in-class ataxia telangiectasia-mutated and Rad3-related protein kinase (ATR) inhibitor. This trial assessed the safety, preliminary efficacy, and tolerance of berzosertib in oesophageal cancer (A1 cohort) with RT and advanced solid tumours (A2 cohort) with cisplatin and capecitabine.

**Methods:**

Single-arm, open-label dose-escalation (Time-to-Event Continual Reassessment Method) trial with 16 patients in A1 and 18 in A2. A1 tested six dose levels of berzosertib with RT (35 Gy over 15 fractions in 3 weeks).

**Results:**

No dose-limiting toxicities (DLTs) in A1. Eight grade 3 treatment-related AEs occurred in five patients, with rash being the most common. The highest dose (240 mg/m^2^) was determined as the recommended phase II dose (RP2D) for A1. Seven DLTs in two patients in A2. The RP2D of berzosertib was 140 mg/m^2^ once weekly. The most common grade ≥3 treatment-related AEs were neutropenia and thrombocytopenia. No treatment-related deaths were reported.

**Conclusions:**

Berzosertib combined with RT is feasible and well tolerated in oesophageal cancer patients at high palliative doses. Berzosertib with cisplatin and capecitabine was well tolerated in advanced cancer. Further investigation is warranted in a phase 2 setting.

**Clinical trials identifier:**

EU Clinical Trials Register (EudraCT) - 2015-003965-27 ClinicalTrials.gov - NCT03641547.

## Background

Radiotherapy (RT) is a standard of care treatment for a number of tumour types, including oesophageal cancer [1]. However, treatment resistance is common and novel therapeutic strategies are needed to improve efficacy [2]. Activation of the DNA damage response (DDR) pathways aids cellular recovery from radiation and targeting these mechanisms has been identified as an attractive strategy for overcoming radio-resistance [3]. Ataxia telangiectasia and Rad3-related protein kinase (ATR) plays critical roles in the DDR by regulating cell cycle checkpoint control and activating the repair of damaged DNA by homologous recombination [4]. Berzosertib is a first in class, selective and potent inhibitor of ATR and several in vitro and in vivo preclinical studies have shown the radiation sensitising effects of ATR inhibition including synergy with berzosertib in oesophageal cancer models [5–7]. Notably, ‘normal cells’ of the gastro-intestinal tract in vivo were able to tolerate berzosertib combined with radiotherapy without significant toxicity suggesting that normal tissues may be ‘spared’ [5].

In the context of oesophageal cancer treatment chemo-radiotherapy may be employed with both palliative and curative intent and commonly cisplatin and a fluoropyrimidine (either 5FU or capecitabine) are the chemotherapy agents of choice to be given concurrently [1]. However, efficacy is limited and even when used with curative intent treatment failure is common with a typical median overall survival of 14–34 months in this setting [8–11]. In the TROG 03.01 study, the use of palliative radiotherapy for malignant dysphagia was investigated, both with and without concurrent chemotherapy [12]. Their research demonstrated that conventional, moderate-dose radiotherapy (30–35 Gy) stands as an effective standard of care of treatment. However, when concurrent chemotherapy is introduced, it substantially amplifies the risk of toxicity, leading to grade 3–4 acute toxicity in 36% of patients, as opposed to 16% in the radiotherapy-alone group (*P*  =  0.0017) [12]. In the setting of preoperative chemoradiotherapy for oesophageal cancer, pathological complete response rates are observed in 49% of squamous cell carcinomas (SCC) and only 23% in adenocarcinomas (ACA), suggesting distinct radiation responses in these histological subtypes [2]. Additionally, for conventional radiotherapy, locoregional failure rates are approximately 50% [13].

Cisplatin is a DNA-damaging platinum-based chemotherapy agent that promotes the formation of intra-strand DNA crosslinks thereby inducing replication fork collapse. Preclinical studies have shown that ATR is activated following cisplatin treatment and that ATR inhibition can profoundly sensitise tumour cells, including oesophageal cancer cells to platinum-based chemotherapy and radiotherapy [14–17]. TP53 is mutated in up to 70% of oesophageal cancers and the loss of TP53 function may also sensitise the tumour selectively to the inhibition of ATR through synthetic lethality, although it should be noted that 15–30% of patients aged over 70 may carry heterozygous TP53 mutants in normal oesophagus [18–20]. Early-phase clinical studies have now shown that the combination of cisplatin and berzosertib is well tolerated with some preliminary evidence of clinical activity [21–23].

The combined in vitro and preclinical model studies suggest that the combination of radiation with an ATR-inhibitor will be an efficacious combination in human studies [17]. Here, we present the results of a phase I dose escalation study designed to assess the safety, tolerability and preliminary activity of escalating doses of berzosertib combined with palliative radiotherapy to the oesophagus (cohort A1). We also evaluated a separate dose escalation cohort (A2) combining berzosertib with cisplatin and capecitabine in patients with any solid tumour designed to inform dosing for a future chemo-radiotherapy combination study with berzosertib in oesophageal cancer.

## Methods

### Study design and treatment

CHARIOT was an all UK-based multi-centre open label non-randomised phase I dose escalation study using the Time-To-Event Continual Reassessment Method (TiTE-CRM) to find the optimal treatment schedule and evaluate the safety of berzosertib.

Berzosertib was assessed in combination with fractionated radiotherapy in palliative treatment of oesophageal cancer (cohort A1), or in combination with cisplatin-capecitabine chemotherapy in solid tumours (cohort A2). The TiTE-CRM method uses critical toxicity summaries (dose-limiting toxicities) of accumulated patient data from all participants treated with at least one dose of the investigational medical product (IMP) within the corresponding trial stage and for whom up-to-date data has been provided to model the dose-toxicity relationship [24]. It is not necessary for a patient to complete the full observation period before consenting the next patient. This results in a better estimation of the maximum tolerated dose (MTD) and shorter study duration and can be advantageous in radiotherapy trials where the toxicity follow-up phase is longer. This manuscript reports cohorts A1 and A2.

The study was conducted according to International Conference on Harmonisation of Good Clinical Practice guidelines and the principles of the Declaration of Helsinki. The protocol was approved by the South-Central Oxford A Ethics Committee(16/SC/0395). All patients provided written informed consent before enrolment. The complete study protocol is available in the Supplementary Materials and Methods.

In cohort A1, palliative radiotherapy was a planned, single-phase treatment delivered with Intensity Modulated Radiotherapy (IMRT). 3D conformal treatment was allowed if IMRT was not possible as long as normal tissue dose constraints were met. Where possible, 4DCT planning scans were used for distal tumours. The total dose of radiation was 35 Gy in 15 fractions treating once daily with 2.3 Gy/fraction, 5 days a week Monday to Friday and prescribed and recorded as per ICRU 62. RT Quality Assurance involved timely retrospective review of radiotherapy plans, but real time review was not required. For cohort A2, 6 cycles of cisplatin 60 mg/m^2^ on Day 1 and capecitabine 625 mg/m^2^ twice daily on a 21-day cycle were administered in combination with berzosertib (see Tables 1a, b in supplementary text for dose levels tested). Dose levels were selected in consultation with Merck KGaA and in reference to prior combination with platinum-based chemotherapy [22, 23].

**Table 1:** Baseline characteristics.

Category	A1 (*n* = 16)	A2 (*n* = 20)	Total (*n* = 36)
Withdrew before trial treatment	0	2 (10.0%)	2 (5.6%)
Sex			
Male	14 (87.5%)	9 (45.0%)	23 (63.9%)
Female	2 (12.5%)	11 (55.0%)	13 (36.1%)
Ethnicity			
White British	16 (100.0%)	20 (100.0%)	36 (100.0%)
Age (years)^a^	65.0 (59.0-75.3)	64.5 (53.5-68.0)	64.5 (56.0-69.3)
WHO PS			
0	14 (87.5%)	16 (80.0%)	30 (83.3%)
1	2 (12.5%)	4 (20.0%)	6 (16.7%)
Primary tumour location			
Oesophagus	16 (100.0%)	0 (0.0%)	16 (44.4%)
Melanoma	0 (0.0%)	4 (20.0%)	4 (11.1%)
GI adenocarcinoma	0 (0.0%)	3 (15.0%)	3 (8.3%)
Cholangiocarcinoma	0 (0.0%)	2 (10.0%)	2 (5.6%)
Oesophageal SCC	0 (0.0%)	2 (10.0%)	2 (5.6%)
Sarcoma	0 (0.0%)	2 (10.0%)	2 (5.6%)
Urothelial Cancer	0 (0.0%)	2 (10.0%)	2 (5.6%)
Ductal Breast Cancer	0 (0.0%)	1 (5.0%)	1 (2.8%)
Pancreatic NET	0 (0.0%)	1 (5.0%)	1 (2.8%)
Papillary thyroid Cancer	0 (0.0%)	1 (5.0%)	1 (2.8%)
Tumour sub-location (A1 only)			
Lower thoracic and abdominal portion	8 (50.0%)		
Mid thoracic portion	5 (31.2%)		
Upper thoracic portion	3 (18.8%)		
Tumour length (cm)^a^ (A1 only)	5.1 (3.6-8.4)		
Histology (A1 only)			
Adenocarcinoma	10 (62.5%)		
Squamous cell carcinoma	6 (37.5%)		
Tumour Grade			
Well differentiated (G1)	1 (6.2%)	1 (5.0%)	2 (5.6%)
Moderately differentiated (G2)	3 (18.8%)	5 (25.0%)	8 (22.2%)
Poorly differentiated (G3)	8 (50.0%)	7 (35.0%)	15 (41.7%)
Unknown or cannot be assessed (GX)	4 (25.0%)	7 (35.0%)	11 (30.6%)
Locoregional Disease			
Yes	13 (81.2%)	10 (50.0%)	23 (63.9%)
No	3 (18.8%)	10 (50.0%)	13 (36.1%)
Distant Metastases			
Yes	6 (37.5%)	19 (95.0%)	25 (69.4%)
No	10 (62.5%)	1 (5.0%)	11 (30.6%)
Prior Radiotherapy			
Yes	1 (6.2%)	8 (40.0%)	9 (25.0%)
No	15 (93.8%)	12 (60.0%)	27 (75.0%)
Prior Systematic Treatment			
Yes	13 (81.2%)	19 (95.0%)	32 (88.9%)
No	3 (18.8%)	1 (5.0%)	4 (11.1%)
Oesophageal Stent (A1 only)			
Yes	0 (0.0%)		
No	16 (100.0%)		

^a^Data are median (interquartiles).

### Patients

For both stages, A1 and A2, eligible patients were ≥16 years of age, with ECOG performance score 0–1 and life expectancy of at least 12 weeks with adequate bone marrow, liver and kidney function, with previous chemotherapy completed at least 28 days before first study dose. Additionally, for Stage A1, eligible patients required histologically confirmed ACA or SCC of the oesophagus, suitable for palliative RT, tumour length 15 cm or less, no oesophageal stent in situ. For Stage A2, eligible patients required histologically confirmed solid metastatic or unresectable tumour where cisplatin-capecitabine treatment was deemed appropriate.

Key exclusion criteria included patients with multiple or untreated brain metastases, significant cardiovascular event within 6 months before study entry, symptomatic arrhythmia or requiring treatment, uncontrolled hypertension, second or third-degree heart block or prolonged QTc interval on ECG (>450 ms in males, >470 ms in females), presence of trachea-oesophageal fistula or invasion of the tracheo-bronchial tree, and use of strong CYP3A inhibitors and inducers or haemopoetic growth factors within 14 days before start of treatment. For Stage A2, patients with dihydropyrimidine dehydrogenase were excluded.

### Study assessment and end-points

The primary objective of Stage A1 was to determine the RP2D of berzosertib that can be administered concomitantly with palliative radiotherapy in oesophageal cancer. The primary objective of Stage A2 was to determine the RP2D of berzosertib that can be administered concomitantly with cisplatin and capecitabine chemotherapy in solid tumours. Secondary objectives for both A1 and A2 included (1) safety and toxicity profile of the combination, (2) feasibility of delivering the combination and (3) efficacy of the combination.

The corresponding primary end-point was the highest treatment schedule resulting in a ≤25% dose limiting toxicity (DLT) rate for A1 and ≤30% DLT rate for A2; corresponding secondary end-points were (1) Grade ≥3 toxicity rate and time for toxicity resolution, (2) proportion of patients completing at least 75%, 90% and 100% of the planned dose of Radiotherapy (A1) and chemotherapy (A2) and (3) objective tumour response by RECIST 1.1, PFS and OS (and in-field RT control rate in A1).

Treatment-related side-effects were reported as per National Cancer Institute (NCI) Common Terminology Criteria for Adverse Events version 4.03. The DLT assessment window was confined to weeks 1–9 for A1 and weeks 1–4 for A2. DLT was generally defined as grade ≥3 haematological or organ toxicity or cardiac abnormalities. Detailed DLT criteria are provided in the supplementary information. In Cohort A1, adverse events and blood tests were assessed weekly by a research physician for the initial 4 weeks, and then every three weeks up to the 12th week. In Cohort A2, toxicities were evaluated weekly throughout treatment weeks 1–18. Objective tumour responses were assessed using computed tomography at week 12 in Cohort A1 and in weeks 6, 12, 18 and at 8 weeks post end of treatment in Cohort A2. Responses were assessed by the investigator according to RECIST version 1.1.

### Statistical analysis

A1 and A2 both had a maximum sample size of 20 patients, both featured early-stopping rules. The sample size estimates were based on 1000 simulated Time-to-Event Continual Reassessment Method (TiTE-CRM) trials that used the same characteristics that the actual trial was based on. No formal power calculation was performed for this Phase I study.

This trial used a (TiTE-CRM) trial design to evaluate the safety of berzosertib in combination with radiotherapy in patients with oesophageal cancer in the palliative setting (Stage A1) or chemotherapy (cisplatin and capecitabine) in patients with any metastatic disease in the palliative setting (Stage A2). Patients in A1 were followed up for DLTs for nine weeks, patients in A2 had a DLT window of four weeks. Data is separated by Stage and presented by dosing schedule allocated where appropriate. Trial design parameters used in both stages are included in the Supplementary information.

The definition of the MTD for both Stages is given as the treatment schedule that is closest to but not above the target toxicity level. During dose decisions, no dose skipping when escalating was permitted, there was no restrictions on dose de-escalation. In stage A1 starting dose was 140 mg/m^2^ IV once weekly, in stage A2 starting dose was 90 mg/m2 IV once weekly.

The trial was not randomised or blinded. Patients were assigned a treatment schedule based on all available safety data and the TiTE-CRM model producing a recommended dose for the next patient. The weight function used was $w=\frac{1}{2}\left(\frac{t}{T}+\frac{d}{D}\right)$ with *t* being time the patient has been followed up for, *T* is max follow up length, *d* is number of doses the patient has received and *D* the maximum number of doses for the schedule they have been prescribed to. The non-independent Trial Management Group (TMG) made decisions on allocation of next dose, using the TiTE-CRM’s recommendations as guide for both cohorts. These decisions were reviewed regularly (at least annually) by and an independent safety review committee (SRC). If there was disagreement amongst TMG members on the next allocation, the SRC would be contacted for arbitration. Trial conduct (i.e., recruitment rates, protocol deviations, etc.) was reviewed approximately bi-annually by another independent oversight committee.

The primary safety population included any patient who received at least one dose of berzosertib. All patients who received treatment within the study were evaluable for response.

Data are analysed and reported on all available data, no imputation has been used in any primary interim or final analyses.

Planned early stopping rules were included in this study. These were:

- **Safety**: A Stage will stop for safety if, at any point in the trial, there is sufficient evidence to suggest that schedule 1 is too toxic. More specifically, we will consider schedule 1 to be too toxic if, given all the available data, there is a high probability that the DLT rate is greater than the target toxicity level, (i.e. P(Toxicity at treatment schedule 1 > Target Toxicity Level | data) >0.95).
- **A1 Success**: Stage A1 will stop for success when either a total of 10 patients have been assigned to a particular treatment schedule or 20 patients have been recruited, whichever occurs first.
- **A2 Success**: The trial will stop for success when either six patients have been assigned to the fourth treatment schedule (140 mg/m^2^ of berzosertib) twice weekly) or 20 patients in total have been recruited, whichever occurs first.

Four sensitivity analyses were presented for each dose decision meeting, they were analysed using the TiTE-CRM model as in the primary analysis. For each of these analyses the posterior probabilities of toxicity at each dose level and their associated equal-tailed 95% credible interval were presented. These analyses were:

1. Only using those patients who did not miss any of their dose prescribed on their dose schedule, and weighted using the original TiTE-CRM weights, i.e. weighting only according to length of follow-up and not taking account of how much dose has been received
2. Only using those patients who have received at least 75% of the prescribed dose, using the same weight function as in the main analysis
3. Only using those patients who have received at least 75% of the prescribed dose, but using the original TiTE-CRM weights
4. Using the same population and weighting as the primary population but assuming the ‘Most Toxic’ Scenario, i.e., all patients currently on treatment within the DLT window have been assigned a DLT.

There are no pre-specified subgroup analyses.

All analyses were performed using R version 4.1.0 (2021-05-18) [25] and OpenBUGS V3.2.3 [26]. Code used to produce results will not be available to share.

## Results

Thirty-six patients (A1 = 16; A2 = 20) were recruited between December 2018 and March 2022. The A1 component stopped short of its original recruitment target of 20 patients due to conclusion of planned recruitment period. At that point, the A1 cohort was one patient away from hitting an early stopping rule for the success of recruiting 10 on a schedule (Schedule 6). Although 20 patients were recruited to A2, two patients withdrew before any trial treatment was delivered.

### Patient demographics and disposition

The median ages (Inter Quartiles) of patients recruited to A1 and A2 were 65 (59.0–75.3) and 64.5 (53.5–68.0), respectively. In the A1 cohort, 14/16 (87.5%) of participants were male and all had a diagnosis of oesophageal cancer (6 SCC and 10 ADA). In A2, 9/20 (45%) of participants were male and the most common tumour subgroup was melanoma. Further details of baseline patient and disease characteristics of the 36 enroled patients are presented in Table 1.

In A1, all 16 patients were evaluable for primary safety analysis and 12/16 were assessable for 12-week overall RECIST efficacy analysis. In A2, 18/20 patients were evaluable for primary safety analysis and 8/20 were assessable for 12-week overall RECIST efficacy analysis.

The patient flow for A1 and A2 are described in CONSORT diagrams in the Supplementary text.

### Dose escalation, DLTs and MTD

In the A1 cohort, 16 patients were enroled and completed final (week 12) visit. No DLTs were reported in cohort A1. The TiTE-CRM recommended dose escalation for each dose level until the maximum level. Berzosertib 240 mg/m2 twice weekly (schedule 6) was identified as the RP2D in this combination.

In the A2 cohort, 18 patients started treatment, with two patients experiencing DLT; grade 3 neutropenia and grade 3 pyrexia in the first patient and sepsis, vomiting and dehydration (all grade 3) in the second patient. Both patients who experienced DLT received berzosertib at 140 mg/m² once a week (schedule 3). Although no dose-limiting toxicities (DLTs) were recorded according to the study protocol with 140 mg/m² twice a week (schedule 4), some patients experienced toxicities that nearly met the definition of DLTs, such as toxicity that resulted in the missed administration of two consecutive doses of berzosertib across cycles. DLTs observed in both cohort A1 and A2 are presented in Table 2.

**Table 2:** Dose Limiting Toxicities observed in cohort A1 and A2.

	A1			A2		
Schedule	*N*	Number of patients with DLTs	Posterior estimates of toxicity (95% Cred. Interval)	*N*	Number of patients with DLTs	Posterior estimates of toxicity (95% Cred. Interval)
**1**	3	0	0.008 (0.000, 0.064)	4	0	0.091 (0.009, 0.256)
**2**	1	0	0.012 (0.000, 0.085)	1	0	0.111 (0.014, 0.291)
**3**	1	0	0.016 (0.000, 0.108)	9	2	0.146 (0.026, 0.345)
**4**	1	0	0.019 (0.000, 0.124)	4	0	0.185 (0.042, 0.397)
**5**	1	0	0.023 (0.000, 0.140)			
**6**	9	0	0.029 (0.000, 0.165)			

Number of participants included in the primary safety analysis and 12-week overall RECIST efficacy reporting for both stages are included in the Supplementary Information. The final TiTE-CRM model estimates are presented further in Table 3.

**Table 3:** a: Five most common system organ classes to experience adverse events by grade in A1. b: Five most common system organ classes to experience adverse events by grade in A2.

	One (*n* = 66)	Two (*n* = 19)	Three (*n* = 16)	Four (*n* = 0)	Total (*n* = 101)
**a**					
System Organ Class – A1					
Gastrointestinal Disorders	20 (30.3)	6 (31.6)	2 (12.5)	0 (NaN)	28 (27.7)
Skin and subcutaneous tissue disorders	8 (12.1)	2 (10.5)	4 (25.0)	0 (NaN)	14 (13.9)
Respiratory, thoracic and mediastinal disorders	11 (16.7)	0 (0.0)	0 (0.0)	0 (NaN)	11 (10.9)
General Disorders & Administration Site Conditions	6 (9.1)	2 (10.5)	2 (12.5)	0 (NaN)	10 (9.9)
Investigations	4 (6.1)	0 (0.0)	3 (18.8)	0 (NaN)	7 (6.9)

	One (*n* = 159)	Two (*n* = 54)	Three (*n* = 26)	Four (*n* = 3)	Total (*n* = 242)
**b**					
System Organ Class – A2					
General Disorders & Administration Site Conditions	27 (17.0)	16 (29.6)	0 (0.0)	0 (0.0)	43 (17.8)
Gastrointestinal Disorders	33 (20.8)	5 (9.3)	1 (3.8)	0 (0.0)	39 (16.1)
Blood & Lymphatic System Disorders	12 (7.5)	9 (16.7)	13 (50.0)	3 (100.0)	37 (15.3)
Investigations	18 (11.3)	6 (11.1)	1 (3.8)	0 (0.0)	25 (10.3)
Infections & Infestations	14 (8.8)	5 (9.3)	1 (3.8)	0 (0.0)	20 (8.3)

Berzosertib 140 mg/m^2^ once weekly (schedule 3) was identified as the RP2D in the A2 combination. This was a dose level lower than that identified by TiTE CRM and was selected based on clinical judgement. This was due to clinically unacceptable toxicities observed with dose level 4 which did not reach DLT definition, however resulted in missed administration.

#### Toxicity, safety, treatment compliance

RT metrics: planning target volumes mean 466 cc (range: 257–912), mean lung dose 7.36 Gy (range 3–8.9 Gy), spinal cord max dose to 0.1 cc mean 27.65 Gy (range 34.5–18 Gy). (See supplementary text for full individual patient metrics). RT compliance—14/16 (87.5%) patients completed all 15 fractions of RT and two patients (both schedule 6) missed one fraction each. Neither of the missed fractions was due to toxicity. One was due to a rest day due to machine maintenance, the other due to concern that the radiotherapy dosimetry may have been affected due to changes noted at time of treatment verification on Cone beam CT.

In Cohort A2, 10 out of the 18 (56%) patients who started treatment stopped their capecitabine treatment before the end. The main reasons for early stoppages were Disease Progression and Neutropenia, accounting for 70% (seven out of 10) of all stoppages. 70% (seven out of 10) stoppages occurred in the first two cycles of treatment. Nine out of the 18 (50%) patients who started treatment stopped their Cisplatin treatment before the end. The main reason for early stoppages was Disease Progression 67% (six out of nine) of all stoppages.

In cohort A2, there were five SAEs in the cohort, occurring in three patients: pyrexia, neutropenia, sepsis, vomiting and chest pain. There were no treatment-related deaths.

A summary of all the five most common MedDRA System Organ Classes affected by adverse events for both stages is presented in Table 3a, b by event grade. There were no Grade 4 events in Stage A1, so percentages are non-calculable.

#### Efficacy

**Fig. 1:**
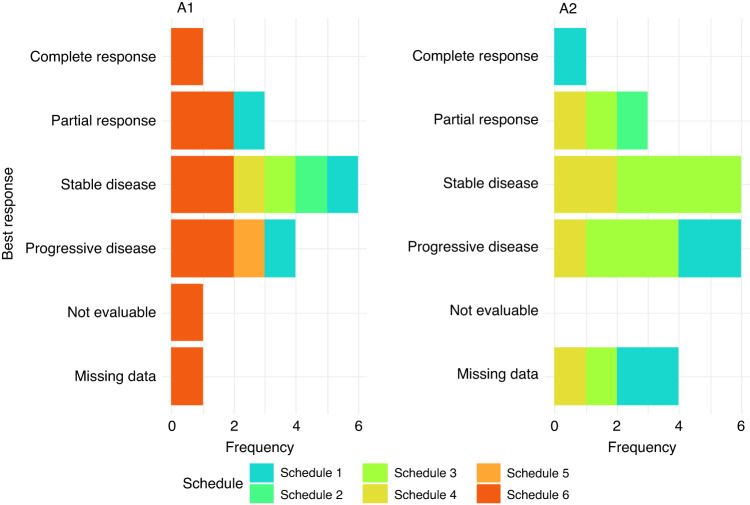
Best overall response for cohorts A1 and A2 by patient treatment schedule. All response categories were determined according to RECIST 1.1 criteria.

**Fig. 2:**
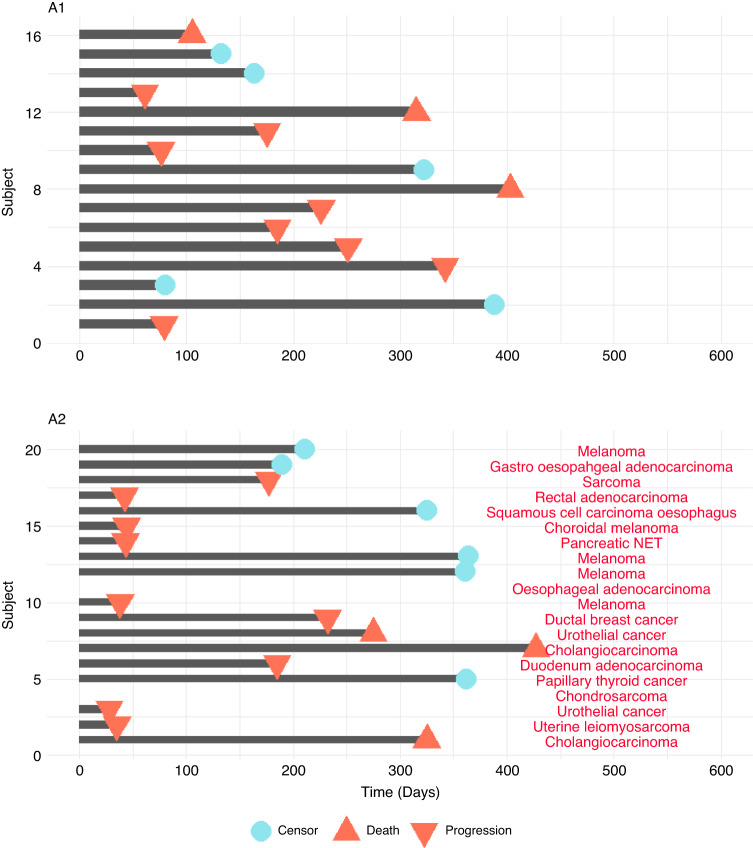
Progression-free survival times for all patients, including censoring and tumour type annotations.

In cohort A1, efficacy data was evaluable for 11 patients, with two showing partial response, six showing stable disease and three showing disease progression at 12 weeks quantified by RECIST 1.1 (Fig. 1). The median progression-free survival was 226 days, with a 95% confidence interval of (175, 342) (Fig. 2). Median overall survival was 394 days with corresponding 95% confidence interval of (263, 403). In-field radiotherapy control was achieved in six patients, four of which had a partial response and two had stable disease.

**Fig. 3:**
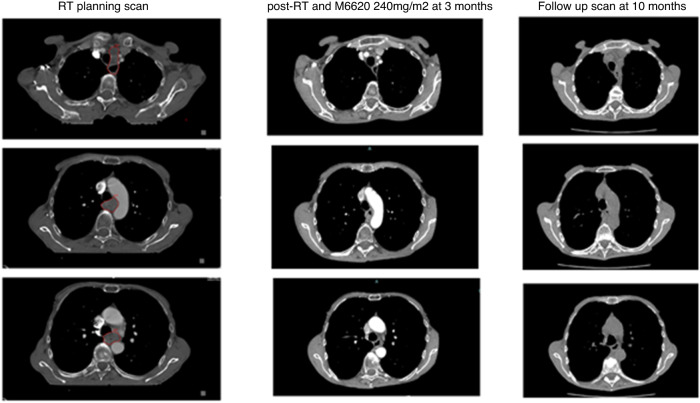
Patient case: 3 axial CT slices at radiotherapy planning, 3 months of treatment w/ berzosertib, 10-month follow-up.

Figure 3 shows the excellent radiographic response of a patient in cohort A1 with a mid-oesophageal SCC at 25 cm T4N2M1 3 months following treatment with RT + berzosertib. A further scan 6-months later shows no reported change in the oesophageal tumour and no size-significant mediastinal adenopathy.

In cohort A2, efficacy data was evaluable for eight patients, with three showing radiographic partial response, four displaying stable disease, and one displaying disease progression quantified by RECIST 1.1 at 12 weeks (Fig. 1). The median progression-free survival was 232 days (95% CI lower bound: 44), and the upper bound was non-calculable (Fig. 2). The median overall survival was 427 days (95% CI lower bound: 249), and the upper bound was also non-calculable.

## Discussion

The conventional chemoradiotherapy for oesophageal cancer can result in considerable treatment-related side effects, however, a complete clinical response is achieved only in a minority of patients (30%) [2]. Therefore, there remains an unmet clinical need for investigating drug-radiotherapy combinations which may enhance tumour control, reduce side effects and improve patient outcomes [27].

Recently, radiotherapy in combination with radiosensiters such as PARP inhibitors and immunotherapy have been investigated in oesophageal cancer, indicating a shift towards investigating novel drug-radiotherapy combinations [28, 29]. Despite the promising preclinical and early-phase studies, there has been a dearth of randomised late-phase trials exploring these combinations. As such, there has not yet been success in establishing novel drug-radiotherapy combinations in oesophageal cancer. Our multicentre study has successfully recruited the required target number of patients despite the challenging conditions of the pandemic and has determined the RP2D for further testing in a palliative setting, as well as exploring how the combination therapy could be integrated into curative settings.

In this study, we evaluated two dose escalation cohorts, cohort A1, the combination of berzosertib with palliative radiotherapy in advanced oesophageal cancer and cohort A2, the combination of berzosertib with cisplatin and capecitabine in patients with any solid tumour as a preamble to testing in the definitive setting for chemoradiation in oesophageal cancer.

Berzosertib has previously been shown to be safe and well tolerated alone and in combination with a variety of DNA-damaging cytotoxic drugs [21–23, 30]. Prior evaluation in the phase I monotherapy dose escalation study established the recommended phase II dose (RP2D) for once- or twice-weekly administration at 240 mg/m^2^ [30]. Preliminary evidence of anti-tumour activity and target engagement had been observed as monotherapy and higher doses required non-feasible infusion volumes. In this study, in combination with radiotherapy berzosertib was well tolerated for all dose levels tested including twice-weekly administration at 240 mg/m^2^. The scheduling of radiotherapy and chemotherapy 24 h prior to dosing with berzosertib is based on preclinical data suggesting that the greatest effect of ATR inhibition takes place within the first 24 h after initiation of DNA damage as at this point there is the greatest proportion of cells within S phase of the cell cycle and activation of ATR [30].

Overall, the combination of berzosertib with radiotherapy was considered well tolerated as there were no exacerbations of lung, cardiac or oesophageal toxicities seen due to local thoracic treatment despite some subjects incurring large volumes of thorax being irradiated. A maculopapular rash was the only serious adverse event that was considered possibly related to berzosertib. This was transient and resolved following administration of topical medications.

The majority of patients in A1 (81%) had received at least one line of prior systemic treatment, including checkpoint immunotherapy in two cases due to prior participation in a separate clinical trial. Median overall survival observed for first-line therapy in the treatment of advanced oesophageal cancer varies between 9 and 14 months [31, 32]. In our study for the combination of berzosertib with radiotherapy, median overall survival was 394 days (~13 months). The outcomes compare favourably with TROG 03-01 study ([20]) where the addition of Cisplatin to same radiation dose was at a cost of increased toxicity, with 36% of the patients seeing grade 3 or higher toxicity and a median OS 6.7 months (4.9–8.0). Whilst it is important to reflect that this is a small cohort of patients these data are encouraging for the clinical safety and activity of this combination.

Cohort A2 evaluated the combination of cisplatin, capecitabine and berzosertib with a view to informing dosing for a future chemo-radiotherapy combination study with berzosertib in oesophageal cancer. Expected mechanism-based myelosuppression was the predominant toxicity and two patients experienced dose limiting toxicities. The recommended phase II dosing schedule was determined to be cisplatin 60 mg/m^2^, capecitabine 625 mg/m^2^ and berzosertib 140 mg/m^2^ once weekly. This schedule differed from the recommendation from the prespecified TiTE-CRM model used in the primary analysis but clinical opinion deemed that the higher dose level was not deliverable due to the number of dose reductions and dose delays resulting from haematological toxicity that were not defined as dose-limiting toxicities. In this heavily pre-treated population four patients had objective responses by RECIST 1.1, including one patient with papillary thyroid carcinoma who had a durable complete response to treatment. Median progression-free survival 232 days is suggestive of preliminary activity for this regimen.

Identifying biomarkers for selection of patients to target tumours harbouring ATM truncating mutations or ATM protein loss, TP53 mutations, BRCA1 or BRCA2 mutations, or other alterations conferring homologous recombination repair deficiency is key for treatment success. Oesophageal cancer is a top candidate as it has been shown that accumulated DNA Damage repair (positive staining for γH2AX and phospho-ATM) was evident within tumour tissue and significantly increased in non-malignant tissue surrounding the tumour cells although activation of p53 by phosphorylation at serine 15 was observed only in tumour tissue ([32]) highlighting the opportunity for enhancing tumour kill without normal tissue damage when radiation is used.

This study has several limitations. Firstly, the small number recruited and the absence of pharmacodynamic data makes it challenging to precisely understand the radiation dose-response relationship of berzosertib. As often in phase I studies the absence of comparative arms involving chemotherapy, ATR inhibition, or radiation therapy hinders our ability to make direct comparisons among these treatment modalities. Lastly, late toxicities due to chemoradiation such as oesophageal stricturing were not assessed beyond the 12-week follow-up.

To address these limitations and refine the understanding of the optimal combination of systemic therapy, ATR inhibition, and radiation therapy in curative radiotherapy setting, further dose-finding investigations are warranted.

In summary, this study is the first clinical trial to evaluate the combination of an ATR inhibitor with radiotherapy. Berzosertib is well tolerated when scheduled with radiotherapy and encouraging clinical activity was observed. We consider that further clinical study is warranted, including combination with chemo-radiotherapy.

### Supplementary information

Supplementary information figures and tables

Supplementary information - CHARIOT Protocol

Supplementary information - CHARIOT Radiotherapy Quality Assurance Summary

## Data Availability

The data collected for the study, including individual participant data and a data dictionary defining each field in the set, will be made available to researchers on request to the study team and with appropriate reason, via octo-enquiries@oncology.ox.ac.uk. The shared data will be de-identified participant data and will be available for 5 years following publication of the study. Data will be shared with investigator support, after approval of a proposal and with a signed data access agreement.

## References

[1] Allum WH, Blazeby JM, Griffin SM, Cunningham D, Jankowski JA, Wong R (2011). Guidelines for the management of oesophageal and gastric cancer. Gut.

[2] van Hagen P, Hulshof MCCM, van Lanschot JJB, Steyerberg EW, van Berge, Henegouwen MI (2012). Preoperative chemoradiotherapy for esophageal or junctional cancer. N Engl J Med.

[3] Huang R, Zhou PK. DNA damage response signaling pathways and targets for radiotherapy sensitization in cancer. Sig Transduct Target Ther. 2020;5:1–27. 10.1038/s41392-020-0150-x PMC7192953 32355263

[4] Blackford AN, Jackson SP (2017). ATM, ATR, and DNA-PK: the trinity at the heart of the DNA damage response. Mol Cell.

[5] Fokas E, Prevo R, Pollard JR, Reaper PM, Charlton PA, Cornelissen B (2012). Targeting ATR in vivo using the novel inhibitor VE-822 results in selective sensitization of pancreatic tumors to radiation. Cell Death Dis.

[6] Pires IM, Olcina MM, Anbalagan S, Pollard JR, Reaper PM, Charlton PA (2012). Targeting radiation-resistant hypoxic tumour cells through ATR inhibition. Br J Cancer.

[7] Leszczynska KB, Dobrynin G, Leslie RE, Ient J, Boumelha AJ, Senra JM (2016). Preclinical testing of an Atr inhibitor demonstrates improved response to standard therapies for esophageal cancer. Radiother Oncol.

[8] Wong RK, Malthaner RA, Zuraw L, Rumble RB, Cancer Care Ontario Practice Guidelines Initiative Gastrointestinal Cancer Disease Site Group. (2003). Combined modality radiotherapy and chemotherapy in nonsurgical management of localized carcinoma of the esophagus: a practice guideline. Int J Radiat Oncol Biol Phys.

[9] Crosby T, Hurt CN, Falk S, Gollins S, Staffurth J, Ray R (2017). Long-term results and recurrence patterns from SCOPE-1: a phase II/III randomised trial of definitive chemoradiotherapy +/− cetuximab in oesophageal cancer. Br J Cancer.

[10] Hulshof MCCM, Geijsen ED, Rozema T, Oppedijk V, Buijsen J, Neelis KJ (2021). Randomized study on dose escalation in definitive chemoradiation for patients with locally advanced esophageal cancer (ARTDECO study). J Clin Oncol.

[11] Conroy T, Galais MP, Raoul JL, Bouché O, Gourgou-Bourgade S, Douillard JY (2014). Definitive chemoradiotherapy with FOLFOX versus fluorouracil and cisplatin in patients with oesophageal cancer (PRODIGE5/ACCORD17): final results of a randomised, phase 2/3 trial. Lancet Oncol.

[12] Penniment MG, De Ieso PB, Harvey JA, Stephens S, Au HJ, O’Callaghan CJ (2018). Palliative chemoradiotherapy versus radiotherapy alone for dysphagia in advanced oesophageal cancer: a multicentre randomised controlled trial (TROG 03.01). Lancet Gastroenterol Hepatol.

[13] Deng W, Lin SH (2018). Advances in radiotherapy for esophageal cancer. Ann Transl Med.

[14] Pabla N, Huang S, Mi QS, Daniel R, Dong Z (2008). ATR-Chk2 signaling in p53 activation and DNA damage response during cisplatin-induced apoptosis. J Biol Chem.

[15] Wengner AM, Siemeister G, Lücking U, Lefranc J, Wortmann L, Lienau P (2020). The novel ATR inhibitor BAY 1895344 is efficacious as monotherapy and combined with DNA damage-inducing or repair-compromising therapies in preclinical cancer models. Mol Cancer Ther.

[16] Reaper PM, Griffiths MR, Long JM, Charrier JD, Maccormick S, Charlton PA (2011). Selective killing of ATM- or p53-deficient cancer cells through inhibition of ATR. Nat Chem Biol.

[17] van Bijsterveldt L, Durley SC, Maughan TS, Humphrey TC (2021). The challenge of combining chemo- and radiotherapy with checkpoint kinase inhibitors. Clin Cancer Res.

[18] Fisher OM, Lord SJ, Falkenback D, Clemons NJ, Eslick GD, Lord RV (2017). The prognostic value of TP53 mutations in oesophageal adenocarcinoma: a systematic review and meta-analysis. Gut.

[19] Murai K, Dentro S, Ong SH, Sood R, Fernandez-Antoran D, Herms A (2022). p53 mutation in normal esophagus promotes multiple stages of carcinogenesis but is constrained by clonal competition. Nat Commun.

[20] Middleton MR, Dean E, Evans TRJ, Shapiro GI, Pollard J, Hendriks BS (2021). Phase 1 study of the ATR inhibitor berzosertib (formerly M6620, VX-970) combined with gemcitabine ± cisplatin in patients with advanced solid tumours. Br J Cancer.

[21] Shapiro GI, Wesolowski R, Devoe C, Lord S, Pollard J, Hendriks BS (2021). Phase 1 study of the ATR inhibitor berzosertib in combination with cisplatin in patients with advanced solid tumours. Br J Cancer.

[22] Telli ML, Tolaney SM, Shapiro GI, Middleton M, Lord SR, Arkenau HT (2022). Phase 1b study of berzosertib and cisplatin in patients with advanced triple-negative breast cancer. NPJ Breast Cancer.

[23] Cheung YK, Chappell R (2000). Sequential designs for phase I clinical trials with late-onset toxicities. Biometrics.

[24] R Core Team (2021). R: a language and environment for statistical computing. R Foundation for Statistical Computing, Vienna, Austria. (Accessed 20 Oct 2023].

[25] Lunn DJ, Thomas A, Best N, Spiegelhalter D (2000). WinBUGS—a Bayesian modelling framework: concepts, structure, and extensibility. Stat Comput.

[26] Sharma RA, Plummer R, Stock JK, Greenhalgh TA, Ataman O, Kelly S (2016). Clinical development of new drug-radiotherapy combinations. Nat Rev Clin Oncol.

[27] Sheikh H, Ryder D, Bateman A, Chalmers A, & Jackson A. Radiotherapy and olaparib in combination for carcinoma of the oesophagus: a phase I study. Clin Transl Radiat Oncol, 2023;40. 10.1016/j.ctro.2023.100614 PMC10025123 36949958

[28] Middleton M, Karydis I, Lu X, Ryan A, Venhaus R, Ramirez K (2023). Long-term outcomes from adding durvalumab to neoadjuvant treatment of operable gastroesophageal cancers: Results from a multicenter study LUD2015-005. J Clin Oncol.

[29] Yap TA, O’Carrigan B, Penney MS, Lim JS, Brown JS, de Miguel Luken MJ (2020). Phase I trial of first-in-class ATR inhibitor M6620 (VX-970) as monotherapy or in combination with carboplatin in patients with advanced solid tumors. J Clin Oncol.

[30] Cunningham D, Starling N, Rao S, Iveson T, Nicolson M, Coxon F (2008). Capecitabine and oxaliplatin for advanced esophagogastric cancer. N Engl J Med.

[31] Sun JM, Shen L, Shah MA, Enzinger P, Adenis A, Doi T (2021). Pembrolizumab plus chemotherapy versus chemotherapy alone for first-line treatment of advanced oesophageal cancer (KEYNOTE-590): a randomised, placebo-controlled, phase 3 study. Lancet.

[32] He H, Tian D, Guo J, Liu M, Chen Z, Hamdy FC (2013). DNA damage response in peritumoral regions of oesophageal cancer microenvironment. Carcinogenesis.

