# Supplementary material for: CHARIOT: a phase I study of berzosertib with chemoradiotherapy in oesophageal and other solid cancers using time to event continual reassessment method
Source: Br J Cancer. 2023 Dec 21;130(3):467–75. doi: 10.1038/s41416-023-02542-1 (PMC10844302; doi:10.1038/s41416-023-02542-1)
Supplement: Supplementary file 1 — Supplementary information figures and tables [file 41416_2023_2542_MOESM1_ESM.docx]

**Supplementary information**


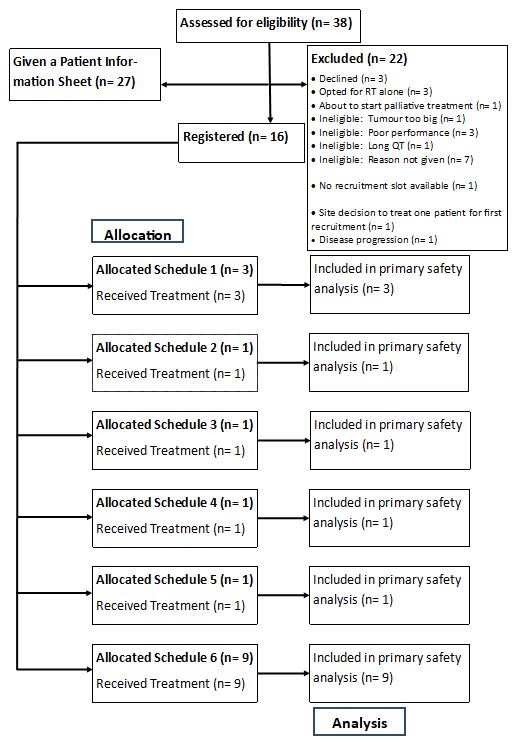


### Figure 1a: A1 CONSORT Diagram


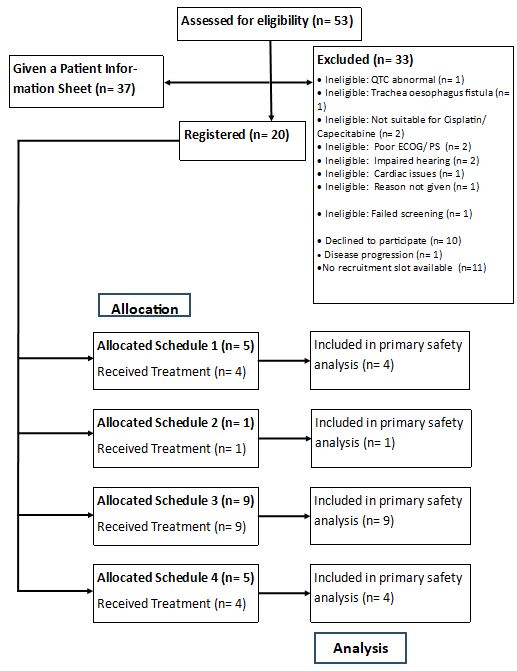


### Figure 1b: A2 CONSORT Diagram

| **Treatment Schedule** | **Dose of berzosertib and delivery days of the schedule** |
| --- | --- |
| 1 | 90mg/m² once a week 3 doses (days 2, 9, 16) by IV infusion |
| 2 | 90mg/m² twice a week 5 doses (days 2, 5, 9, 12, 16) by IV infusion |
| 3 | 140mg/m² twice a week 6 doses (days 2, 5, 9, 12, 16, 19) by IV infusion |
| 4 | 240mg/m² once a week 3 doses (days 2, 9, 16) by IV infusion |
| 5 | 240mg/m² twice a week 5 doses (days 2, 5, 9, 12, 16) by IV infusion |
| 6 | 240mg/m² twice a week 6 doses (days 2, 5, 9, 12, 16, 19) by IV infusion |

### Table 1a: dose levels of berzosertib tested in A1

| **Treatment Schedule** | **Dose of berzosertib and delivery days of the schedule** |
| --- | --- |
| 1 | 90mg/m² once a week for 18 weeks by IV infusion (Tuesdays) |
| 2 | 90mg/m² twice a week for 18 weeks by IV infusion (Tuesdays and Fridays) |
| 3 | 140mg/m² once a week for 18 weeks by IV infusion (Tuesdays) |
| 4 | 140mg/m² twice a week for 18 weeks by IV infusion (Tuesdays and Fridays) |

### Table 1b: dose levels of berzosertib tested in A2


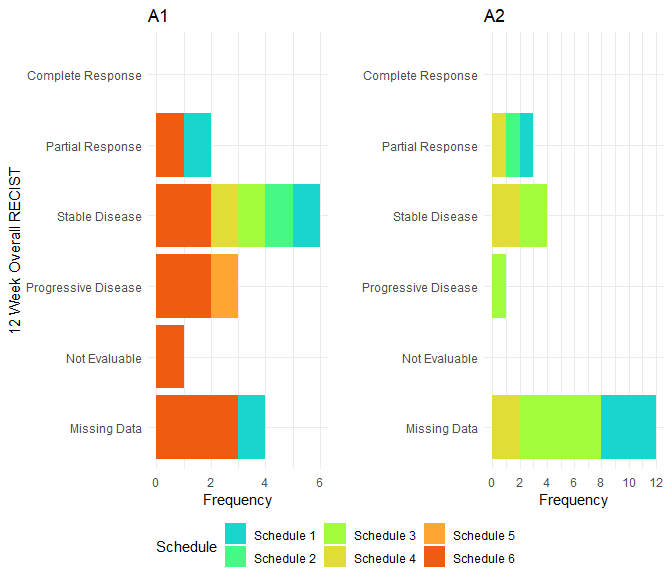


### Figure 2: 12-week response by RECIST

|  | **Schedule 1  (n=3)** | **Schedule 2  (n=1)** | **Schedule 3  (n=1)** | **Schedule 4  (n=1)** | **Schedule 5  (n=1)** | **Schedule 6  (n=9)** | **Total  (n=16)** |
| --- | --- | --- | --- | --- | --- | --- | --- |
| **Primary Safety** | 3 (100.0) | 1 (100.0) | 1 (100.0) | 1 (100.0) | 1 (100.0) | 9 (100.0) | 16 (100.0) |
| **Efficacy (12 Week Overall RECIST)** | 2 (66.7) | 1 (100.0) | 1 (100.0) | 1 (100.0) | 1 (100.0) | 6 (66.7) | 12 (75.0) |

|  | **Schedule 1  (n=5)** | **Schedule 2  (n=1)** | **Schedule 3  (n=9)** | **Schedule 4  (n=5)** | **Total  (n=20)** |
| --- | --- | --- | --- | --- | --- |
| **Primary Safety** | 4 (80.0) | 1 (100.0) | 9 (100.0) | 4 (80.0) | 18 (90.0) |
| **Efficacy (12 Week Overall RECIST)** | 1 (20.0) | 1 (100.0) | 3 (33.3) | 3 (60.0) | 8 (40.0) |

### Table 2: Numbers analysed cohort A1 and A2

| **Haematological:** |
| --- |
| - Absolute neutrophil count (ANC) <0.5 x 10^9^/L for >7days |
| - Febrile neutropenia (fever of unknown origin without clinically or microbiologically documented infection) (ANC <1.0 x 10^9^/L, fever >38.5^o^C) lasting >3 days |
| - Infection (documented clinically or microbiologically) with Grade 3 or 4 neutropenia (absolute neutrophil count <1.0 x 10^9^/L) |
| - Platelets <25 x 10^9^/L |
| - Clinically significant bleeding attributed to grade 3 thrombocytopenia or requiring platelet transfusion |
|  |
| **Organ Toxicity:** |
| - Grade ≥3 oesophagitis onset within 2 weeks of starting radiotherapy |
| - Grade ≥3 pneumonitis onset within 3 months of starting radiotherapy |
| - Grade ≥3 nausea or vomiting not controlled by optimal outpatient anti-emetic treatment |
| - Grade ≥3 diarrhoea despite optimal outpatient anti-diarrhoeal medication use |
| - Other grade 3 ≥ effects thought to be directly treatment related to the combination of M6620 (berzosertib) with radiotherapy |
| - Any toxicity causing a delay of radiotherapy completion by greater than one week |
| - Missing 2 consecutive doses of M6620 (berzosertib) within a cycle due to Grade ≥3 toxicity |
| - A delay of any of the 3 treatments of 7 days or more within a cycle due to treatment related toxicity |
| - An elevation of ALT or AST >5 x ULN lasting 8 days or more |
| - A concurrent elevation of ALT or AST >3 × ULN and total bilirubin >2 × ULN in whom there is no evidence of biliary obstruction or other causes that can reasonably explain the concurrent elevation |
| - Death due to drug related complications |
|  |
| **Cardiac:** |
| - QTc prolongation (any QTc interval ≥500 msec or any change in QTc interval ≥60 msec from baseline) on ECG, unless related to an electrolyte abnormality and prolongation resolves with correction of electrolyte abnormality |
| - Any of the following (CTCAE criteria): Grade 2 or greater ventricular arrhythmia (second or third degree AV block), severe sustained/symptomatic sinus bradycardia less than 45 beats per minute (bpm) or sinus tachycardia >120 bpm not due to other causes (e.g., fever), persistent supraventricular arrhythmia (e.g., uncontrolled/new atrial fibrillation, flutter, atrioventricular nodal tachycardia, etc.) lasting more than 24 hours, ventricular tachycardia defined as >9 beats in a row or any length of torsades de pointes (polymorphic ventricular tachycardia with long QTc), or unexplained recurrent syncope |
| - Symptoms suggestive of congestive heart failure with confirmed Ejection Fraction (EF) <40% (by 2D-echocardiogram or Multiple Gated Acquisition [MUGA] scan) |
| - Troponin T: level which is consistent with myocardial infarction |

### Table 3: Dose limiting toxicities (DLT)

| **Design Parameter** | **Stage A1** | **Stage A2** |
| --- | --- | --- |
| Number of Schedules | 6 | 4 |
| Target Toxicity Level | 0.3 | 0.25 |
| Starting Schedule | 1 | 1 |
| Max Number of Patients | 20 | 20 |
| TiTE Weight Function Used | $w=\frac{1}{2}(\frac{t}{T}+\frac{d}{D})$ | $w=\frac{1}{2}(\frac{t}{T}+\frac{d}{D})$ |
| DLT Window Length | 61 Days | 21 Days |
| Dose Toxicity Curve Model | Power ($dose_{i}^{exp\left( \alpha\right)}$) | Power ($dose_{i}^{exp\left( \alpha\right)}$) |
| Priors | $\alpha\sim N\left( 0,{1.158}^{2} \right)$ | $\alpha\sim N\left( 0,{1.158}^{2} \right)$ |
| Prior Probability of Toxicity on Each Schedule | 0.12, 0.15, 0.18, 0.20, 0.22, 0.25 | 0.17, 0.20, 0.25, 0.30 |

*t = amount of time each patient has been observed, d = amount of M6620 given, T = DLT Window, D = Max M6620 on a given schedule.* *= dose level i, i = 1,…, K. K = 6 for A1, K = 4 for A2.*


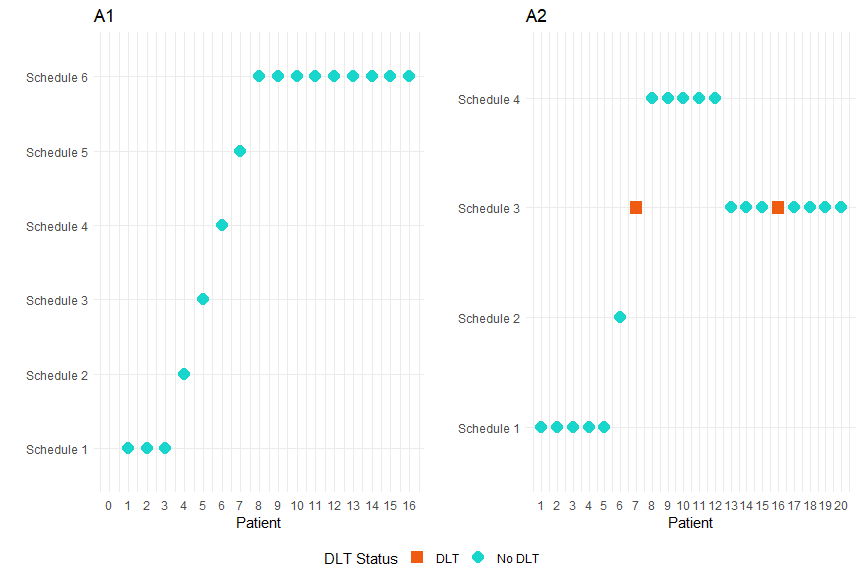


### Figure 3: DLT by dose for A1 and A2 patients
