# Supplementary material for: CHARIOT: a phase I study of berzosertib with chemoradiotherapy in oesophageal and other solid cancers using time to event continual reassessment method
Source: Br J Cancer. 2023 Dec 21;130(3):467–75. doi: 10.1038/s41416-023-02542-1 (PMC10844302; doi:10.1038/s41416-023-02542-1)
Supplement: Supplementary file 2 — Supplementary information - CHARIOT Protocol [file 41416_2023_2542_MOESM2_ESM.docx]

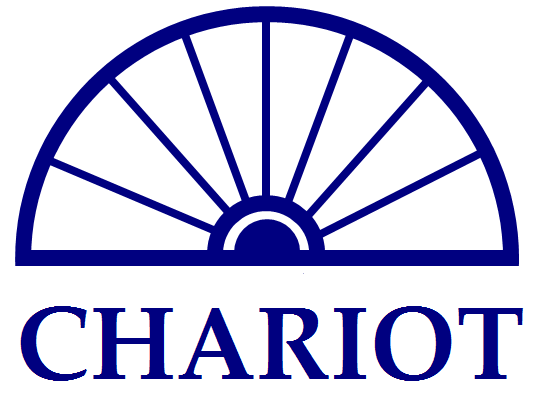


Full title: A phase I dose escalation safety study combining the ATR inhibitor M6620 (Berzosertib) with chemoradiotherapy in oesophageal cancer & other solid cancers using time to event continual reassessment method

Short title: M6620 (Berzosertib) plus standard treatment in oesophageal and other cancer

| **Protocol Version & date:** | CHARIOT_Protocol_V5.0_26Oct2020 |
| --- | --- |
| **Protocol Number:** | OCTO_072 |
| **EudraCT Number:** | 2015-003965-27 |
| **IRAS Project ID:** | 190687 |
| **Ethics Number:** | 16/SC/0395 |

| **Sponsor:** | University of Oxford |
| --- | --- |
| **Funders:** | CRUK Grant Ref: C43735/A20874  Merck KGaA, Darmstadt Germany |
| **Conflict of Interest statement** | None of the protocol authors have declared a potential conflict of interest |
| **Confidentiality Statement** | This document contains confidential information that must not be disclosed to anyone other than the Sponsor, the Trial Office, the Investigator Team, host NHS Trust(s), regulatory authorities, and members of the Research Ethics Committee unless authorised to do so. |

| 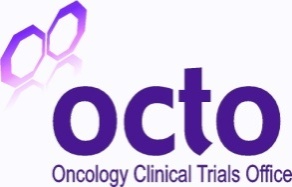 | 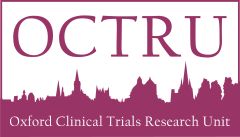 | 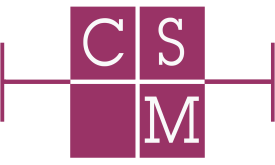 |
| --- | --- | --- |

**Key trial contacts**

| **Chief Investigator** |  |  |
| --- | --- | --- |
|  | Professor Maria A. Hawkins  Medical Physics and Biomedical Engineering  Malet Place Engineering Building  University College London  Gower Street  London, WC1E 6BT |  |
|  | Email: m.hawkins@ucl.ac.uk |  |
| **Co-Investigators** | Dr Somnath Mukherjee  Consultant Clinical Oncologist  Oxford University Hospitals NHS Trust  Cancer and Haematology Centre  Churchill Hospital  Old Road, Oxford, OX3 7LE  Email: somnath.mukherjee@oncology.ox.ac.uk  Dr Simon Lord  Consultant in Medical Oncology & Senior Clinical Researcher in Experimental Cancer Therapeutics  Department of Oncology  University of Oxford, Old Road Campus Research Building  Off Roosevelt Drive, Oxford, OX3 7DQ  Email: simon.lord@oncology.ox.ac.uk |  |
|  | Prof Tim Maughan  Dr Ester Hammond  Dr Ganesh Radhakrishna  Dr David McIntosh  Dr. Paul Shaw |  |
|  |  |  |
| **Oncology Clinical Trial Office (OCTO)** | CHARIOT Trial |  |
|  | Oncology Clinical Trials Office (OCTO) |  |
|  | Department of Oncology, The University of Oxford |  |
|  | Old Road Campus Research Building |  |
|  | Oxford OX3 7DQ, UK |  |
|  | Tel: +44 (0)1865 617084 |  |
|  | Email: octo-chariot@oncology.ox.ac.uk |  |
|  | Website: http://www.oncology.ox.ac.uk/trial/chariot |  |
|  |  |  |
| **Sponsor** |  |  |
|  | University of Oxford Clinical Trials and Research Governance Team |  |
|  | Joint Research Office,  1st Floor, Boundary Brook House |  |
|  | Churchill Drive |  |
|  | Headington  Oxford, OX3 7GB  Email: CTRG@admin.ox.ac.uk |  |
| **Lead NHS Trust** |  |  |
|  | R&D Department, Oxford University Hospitals NHS Trust |  |
|  | Second Floor, OUH Cowley Unipart House Business Centre Garsington Road Oxford OX4 2PG |  |
|  | E-mail: [ouhtma@nhs.net](mailto:heather.house@ouh.nhs.uk) |  |
| **Statistician** | Jane Holmes, Medical Statistician |  |
|  | Centre for Statistics in Medicine Nuffield Department of Orthopaedics, Rheumatology and Musculoskeletal Sciences  The University of Oxford Windmill Road Oxford OX3 7LD |  |
|  | Tel : 01865 223452 |  |
|  | Email: [jane.holmes@csm.ox.ac.uk](mailto:jane.holmes@csm.ox.ac.uk)  AlexAlexander Ooms, Medical Statistician  Centre for Statistics in Medicine Nuffield Department of Orthopaedics, Rheumatology and Musculoskeletal Sciences  The University of Oxford Windmill Road Oxford OX3 7L  Tel: 01865 223453  Email: alexalexander.ooms@csm.ox.ac.uk |  |
|  |  |  |
| **Trial oversight is provided by the Radiotherapy and Imaging Oversight Committee (RIOC).** | |  |
|  |  |  |
| **RIOC Chairman** | Prof Peter Hoskin |  |
|  |  |  |
|  |  |  |
|  |  |  |

**Clinical Queries**

**During office hours**: Clinical Queries should be directed to OCTO Trial Manager (details as above) for action.

**Out of office hours**: Call the Churchill Hospital switchboard on Tel: **01865 741166.** The switchboard team hold emergency contact details for the trial.

**Patient Registration**: To register a patient on the trial please **scan and email as a PDF attachment the registration form to** [**octo-CHARIOT@oncology.ox.ac.uk**](mailto:octo-CHARIOT@oncology.ox.ac.uk)

**Protocol Authors and conflict of interest statement**

Eleni Frangou^*3^ Alex Ooms^*3^

Claire Brooks^*1^ Usharani Wahengbam^*1^

Maria Hawkins^*1^

Jane Holmes^*3^

Stephanie Levy ^*1^

Naomi McGregor^*1^

*There are no relationships/conditions/circumstances that present an actual or potential conflict of interest

^1^University of Oxford, Department of Oncology

^2^Oxford University NHS Trust

^3^University of Oxford, Centre for Statistics in Medicine

**TABLE OF CONTENTS**

Protocol synopsis 9

SUMMARY SCHEDULE OF EVENTS STAGE A1 11

SUMMARY SCHEDULE OF EVENTS STAGE A2 12

SUMMARY SCHEDULE OF EVENTS STAGE B 13

Study Flow Charts 14

Abbreviations 16

1 Introduction 16

1.1 Background 16

1.2 Investigational Medicinal Product(s) used in the study 18

1.3 Pre-clinical rationale 19

1.4 Clinical rationale 19

2 TRial design 20

2.1 Stage A1 21

2.2 Stage A2 21

2.3 Stage B 22

2.4 Duration of patient participation 23

2.5 Post-trial care and follow-up 23

2.6 Recruitment Schematic 24

3 Objectives and endpoints 25

3.1 Stage A 25

3.1.1 Stage A1 25

3.1.2 Stage A2 25

3.2 Stage B 26

4 Patient selection 27

4.1 Eligibility criteria 27

4.2 Protocol deviations and waivers to entry criteria 29

4.3 Re-screening if patient does not meet inclusion/exclusion criteria first time round 30

4.4 Patient registration 30

4.5 Registration procedure 30

5 Trial assessments and procedures 30

5.1 Informed consent 30

5.2 Contraception Requirement and Contraceptive/ Pregnancy counselling 31

5.2.1 Contraception Requirement 31

5.2.2 Female participant of child-bearing potential 31

5.2.3 Male participants 31

5.2.4 Pregnancy counselling 31

5.3 Pre-dosing evaluations (all stages) 31

5.4 Stage A Evaluations 32

5.4.1 Stage A1 evaluations during the study 32

5.4.2 Stage A1 evaluations on early withdrawal 33

5.4.3 Stage A1 off-Study and Follow-up Evaluations 33

5.4.4 Stage A2 evaluations during the study 34

5.4.5 Stage A2 evaluations on early withdrawal 35

5.4.6 Stage A2 off-Study and Follow-up Evaluations 35

5.5 Stage B evaluations 35

5.5.1 Stage B evaluations during the study 35

5.5.2 Stage B evaluations on early withdrawal 36

5.5.3 Stage B off-Study and Follow-up Evaluations 36

6 Early patient withdrawal 37

6.1 Treatment Withdrawal 37

6.2 Consent Withdrawal 37

6.3 Patient evaluability and replacement 37

7 Samples for laboratory analysis 38

7.1 Samples to be analysed in local Trust’s laboratories 38

7.2 Blood and Tissue samples for translational research 38

7.2.1 Biopsy samples 38

7.2.2 Research blood samples 38

7.3 Labelling and confidentiality of samples sent 38

7.4 Clinical reporting of exploratory research assay results 39

7.5 Trial sample retention at end of study 39

7.6 Withdrawal of consent for sample collection and/or retention 39

8 Investigational medicinal products (IMP) 40

8.1 Stage A1 Treatment 40

8.1.1 M6620 (Berzosertib) treatment schedules - Stage A1 40

8.1.2 Radiotherapy dose and duration –Stage A1 40

8.1.3 Stage A1: Dose Escalation Schema 41

8.2 Stage A2 Treatment 42

8.2.1 M6620 (Berzosertib) treatment schedule – Stage A2 42

8.2.2 Chemotherapy dose and duration - Stage A2 42

8.2.3 Stage A2 Dose escalation schema 42

8.3 Stage B Treatment 43

8.3.1 M6620 (Berzosertib) Treatment dose and schedule – Stage B 43

8.3.2 Chemoradiotherapy dose and duration - Stage B 43

8.3.3 Radiotherapy dose and duration - Stage B 43

8.3.4 Stage B Dose frequency escalation schema 44

8.4 Management of M6620 (Berzosertib) drug administration 46

8.5 Management of capecitabine administration 47

8.6 Management of cisplatin administration 47

8.7 Managing delays to Chemotherapy and Radiotherapy 48

8.8 Laboratory values required for administration of chemotherapy 48

8.9 Calculating and recalculating doses 48

8.10 Chemotherapy Dose-banding 48

8.11 Dose capping 48

8.12 Compliance 48

8.13 Management of overdose 48

9 toxicity Management 49

9.1 Dose Limiting Toxicity 49

9.1.1 Reporting a DLT 49

9.1.2 Definition of a DLT 49

9.2 Management of treatment toxicities 51

9.3 Management of M6620 (Berzosertib) drug toxicities 51

9.3.1 Additional guidance Stage A2 52

9.3.2 Toxicities associated with M6620 (Berzosertib) 52

9.4 Management of chemotherapy toxicities 52

9.4.1 Haematological toxicity 52

9.4.2 Non-haematological toxicity 53

9.4.3 Additional capecitabine toxicities 53

9.4.4 Additional Cisplatin toxicities 54

10 Other Treatments (NON-IMPs) 55

10.1 Background systemic therapy 55

10.2 Support medication 55

10.3 Concomitant medication and non-drug therapies 55

10.4 Prohibited therapies 55

10.5 Potential Drug Interactions 55

10.5.1 M6620 (Berzosertib) 55

10.5.2 Capecitabine and Cisplatin 56

11 Drug management 56

11.1 Drug supplies 57

11.1.1 M6620 (Berzosertib) 57

11.1.2 Capecitabine and cisplatin 57

11.2 Drug ordering 57

11.3 IMP Receipt 57

11.4 Handling and storage 57

11.4.1 M6620 (Berzosertib) 57

11.4.2 Capecitabine and cisplatin 57

11.5 Labelling 57

11.5.1 M6620 (Berzosertib) 57

11.5.2 Capecitabine and cisplatin: 57

11.6 Dosing and dispensing 58

11.6.1 M6620 (Berzosertib) 58

11.6.2 Cisplatin and capecitabine 58

11.7 Drug accountability 58

11.8 Drug destruction 58

11.9 Occupational safety 58

12 Radiotherapy (or chemoradiotherapy) 58

12.1 Dose prescription and fractionation 58

12.2 Radiotherapy localisation 59

12.3 Target volume definition (TVD) 59

12.4 Organs at risk 61

12.5 Treatment Plan Optimisation 61

12.6 Dose constraints 61

12.7 Treatment delivery and verification 62

12.8 Management of radiotherapy toxicity 62

12.9 The management of unscheduled gaps in radiotherapy treatment 63

12.10 Radiotherapy quality assurance 64

13 Evaluation of response 64

13.1 Tumour assessment 64

13.2 Tumour response 64

13.3 Other definitions of outcome: 64

14 safety reporting 64

14.1 Adverse Event Definitions 65

14.2 Clinical laboratory abnormalities and other abnormal assessments as AEs and SAEs 65

14.3 Determining adverse event causality 66

14.4 Reference safety information (RSI) for assessment of expectedness 66

14.4.1 Expected adverse events associated with Radiotherapy 67

14.5 Summary of trial safety reporting requirements 67

14.6 Suspected Unexpected Serious Adverse Drug Reactions (SUSARs) 69

14.7 Expedited reporting of SAEs 69

14.8 Follow-up of Serious Adverse Events 69

14.9 Reporting Adverse Events on the CRF 70

14.10 Events exempt from being reported as AE/ SAEs 70

14.11 Informing Investigators of new safety information 70

15 pregnancy 70

16 Defining the end of trial 71

17 Statistical considerations 71

17.1 Sample size and power 71

18 Statistical analysis plan 71

18.1 Inclusion in analysis 71

18.2 Subgroup analysis 72

18.3 Interim Analyses 72

18.4 Procedures for reporting any deviation(s) from the protocol 72

18.5 Analysis for Safety 72

18.6 Final analysis 72

19 Trial committees 72

19.1 Trial Management Group (TMG) 72

19.2 Safety Review Committee 72

19.3 Trial Steering Committee 72

20 Data management 73

20.1 Database considerations 73

20.2 Case reports forms (CRFs) 73

20.3 Accounting for missing, unused, or spurious data. 73

20.4 Clinical study report 73

21 Study site management 73

21.1 Study site responsibilities 73

21.2 Study site protocol deviations 74

21.3 Study site set up and activation 74

21.4 Arrangements for sites outside the UK 74

21.5 Study documentation 74

22 Regulatory and ethical considerations 74

22.1 Ethical conduct of the trial and ethics approval 74

22.2 Regulatory Authority approval 74

22.3 NHS Research Governance 74

22.4 Protocol amendments 74

22.5 Urgent safety measures 75

22.6 Temporary halt 75

22.7 Serious Breaches 75

22.8 Trial Reports 75

23 Expenses and benefits 76

24 Quality assurance 76

24.1 Risk assessment 76

24.2 Monitoring 76

24.3 Audit and Regulatory Inspection 76

25 Records retention & archiving 76

26 Patient confidentiality 77

27 Study funding 77

28 Sponsorship and indemnity 77

28.1 Sponsorship 77

28.2 Indemnity 77

28.3 Contracts/Agreements 77

29 Publication policy 77

30 References 78

31 APPENDIX A: ECOG performance scale 79

32 Appendix B: measurement of disease - recist criteria 79

33 Appendix C: RTOG Late Toxicity score 83

34 Appendix D: Mellow Dysphagia score 83

35 Appendix E: Protocol Amendment history 84

# Protocol synopsis

| Full Title of study: | A phase I dose escalation safety study combining the ATR inhibitor M6620 (Berzosertib) with chemoradiotherapy in oesophageal cancer & other solid cancers using time to event continual reassessment method | |
| --- | --- | --- |
| Short Title: | M6620 (Berzosertib) plus standard treatment in oesophageal and other cancer | |
| Trial Acronym: | CHARIOT | |
| Clinical Phase: | Phase I | |
| Study Design: | Interventional | |
| **Stage A1** | **Objectives** | **Endpoints/ Outcome Measures** |
| Primary: | To determine the best tolerated M6620 (Berzosertib) treatment schedule (or phase II recommended dose (RPTD)) administered concomitantly with radiotherapy (RT) only in the palliative treatment of oesophageal cancer | Highest treatment schedule resulting in less than 25% dose limiting toxicity (DLT) rate (see section 9.1.2 for DLT definitions) |
| Secondary: | - To determine the safety and toxicity profile of M6620 (Berzosertib) administered concomitantly with RT only in the palliative treatment of oesophageal cancer - To determine if M6620 (Berzosertib) can be delivered in combination with palliative RT - Efficacy of the combination | - Any toxicity grade ≥3 graded according to CTCAE v4.03 and length of time for toxicity to resolve - Proportion of patients completing at least 75%, 90% and 100% of the planned RT dose - Objective tumour response (OR) as evaluated by CT scan and quantified by Response Criteria Evaluation (RECIST 1.1) & PFS and OS from D1 - In field radiotherapy control |
| Tertiary: | - Explore tumour characteristics associated with response | - Genotyping of tumours |
| **Stage A2** | **Objectives** | **Endpoints/ Outcome Measures** |
| Primary: | To determine the best tolerated M6620 (Berzosertib) treatment schedule (or phase II recommended dose (RPTD)) administered concomitantly with chemotherapy (Cisplatin and Capecitabine) only in the palliative treatment of solid cancer | Highest treatment schedule resulting in less than 30% dose limiting toxicity (DLT) rate (see section 9.1.2 for DLT definitions) |
| Secondary: | - To determine the safety and toxicity profile of M6620 (Berzosertib) administered concomitantly with chemotherapy (Cisplatin and Capecitabine) only in the palliative treatment of solid cancer - To determine if M6620 (Berzosertib) can be delivered in combination with palliative chemotherapy - Efficacy of the combination | - Any toxicity grade ≥3 graded according to CTCAE v4.03 and length of time for toxicity to resolve - Proportion of patients completing at least 75%, 90% and 100% of the planned dose - Objective tumour response (OR) as evaluated by CT scan and quantified by Response Criteria Evaluation (RECIST 1.1) & PFS and OS from D1 |
| **Stage B** | **Objectives** | **Endpoints/ Outcome Measures** |
| Primary: | To determine the best tolerated M6620 (Berzosertib) treatment schedule (or phase II recommended dose (RPTD)) administered concomitantly with radiotherapy (dCRT) in combination with cisplatin and capecitabine in the radical treatment of oesophageal cancer | Highest treatment schedule resulting in less than 45% dose limiting toxicity (DLT) rate (see section 9.1.2 for DLT definitions) |
| Secondary: | - To determine the safety and toxicity profile of M6620 (Berzosertib) administered concomitantly with dCRT in combination with cisplatin and capecitabine in the radical treatment of oesophageal cancer - To determine tolerance and ability to deliver M6620 (Berzosertib) in combination with standard dCRT - Efficacy and safety of the combination | - Any toxicity grade ≥3 graded according to CTCAE v4.03 and length of time for toxicity to resolve - Treatment tolerance and deliverability measured by proportion of patients completing at least 80% of the planned chemotherapy dose and at least 20 fractions of RT - Objective tumour response (OR) as evaluated by CT scan and quantified by Response Criteria Evaluation (RECIST 1.1) and endoscopic and biopsy findings & PFS and OS from D1 |
| Tertiary | To explore target effects in tissue | - Change in level of ATR inhibition and apoptosis in M6620 (Berzosertib) treated tissue using IHC. - Genotyping of tumours - Aim to identify markers for oesophageal cancer in the blood |
| Planned enrolment: | Stage A1: Maximum 20 participants  Stage A2: Maximum 20 participants  Stage B: Maximum 25 participants | |
| Target Population: | Stage A1: Oesophageal tumours for palliative radiotherapy  Stage A2: Metastatic or advanced inoperable solid tumours for chemotherapy  Stage B: Oesophageal tumours for radical chemoradiotherapy | |
|  | **Name of drug** | **Formulation, dose, route of administration** |
| Investigational Medicinal Product(s) | M6620 (Berzosertib) | Solution for infusion, 90 – 240 mg/m^2^ |
|  | Cisplatin | Solution for infusion, 60 mg/m^2^ |
|  | Capecitabine | Tablet, 625mg/m^2^ bd, oral |
| Other interventions: | Stage A1: Palliative radiotherapy  Stage B: Definitive radiotherapy | |
| Treatment Duration | Stage A1: 3 weeks  Stage A2: maximum 18 weeks  Stage B: 11 weeks | |
| Follow-up duration (last study visit from start of treatment) | Stage A1: 12 weeks  Stage A2: maximum 26 weeks  Stage B: 24 weeks  Participants will be followed up through their medical records at 6 and 12 months for Stage A1 & A2 | |
| End of study | For the purpose of the Research Ethics Committee approval the trial end date will be the Last Patient start of treatment for Stage B plus 24 weeks. | |

## SUMMARY SCHEDULE OF EVENTS STAGE A1

|  |  |  | Week 1 | | | | | | | Week 2 | | | | | | | Week 3 | | | | | | | Week 4**^9^** | Week 9 | Week 12 |
| --- | --- | --- | --- | --- | --- | --- | --- | --- | --- | --- | --- | --- | --- | --- | --- | --- | --- | --- | --- | --- | --- | --- | --- | --- | --- | --- |
| Study Day | Screening | Post Registration | 1 | 2 | 3 | 4 | 5 | 6 | 7 | 8 | 9 | 10 | 11 | 12 | 13 | 14 | 15 | 16 | 17 | 18 | 19 | 20 | 21 | Follow up^8^ | | |
|  |  |  | M | T | W | T | F | S | S | M | T | W | T | F | S | S | M | T | W | T | F | S | S |  |  |  |
| Informed Consent | X |  |  |  |  |  |  |  |  |  |  |  |  |  |  |  |  |  |  |  |  |  |  |  |  |  |
| Screening assessments**^1^** | X |  |  |  |  |  |  |  |  |  |  |  |  |  |  |  |  |  |  |  |  |  |  |  |  |  |
| M6620 (Berzosertib) schedule assignment |  | X |  |  |  |  |  |  |  |  |  |  |  |  |  |  |  |  |  |  |  |  |  |  |  |  |
| Radiotherapy planning | X | |  |  |  |  |  |  |  |  |  |  |  |  |  |  |  |  |  |  |  |  |  |  |  |  |
| Radiotherapy (35GY in 15#) |  |  | X | X | X | X | X |  |  | X | X | X | X | X |  |  | X | X | X | X | X |  |  |  |  |  |
| M6620 (Berzosertib) |  |  |  | X |  |  | X**^2^** |  |  |  | X |  |  | X**^2^** |  |  |  | X |  |  | X**^3^** |  |  |  |  |  |
| Haematology/Biochemistry**^4^** | X |  | X |  |  | X**^2^** |  |  |  | X |  |  | X**^2^** |  |  |  | X |  |  | X**^3^** |  |  |  | X |  |  |
| Coagulation | X |  | X |  |  |  |  |  |  |  |  |  |  |  |  |  |  |  |  |  |  |  |  | X |  |  |
| DLT assessment**^5^** |  |  |  |  |  | X |  |  |  | X |  |  | X**^2^** |  |  |  | X |  |  | X**^3^** |  |  |  | X | X |  |
| AE assessment**^5^** |  |  | X |  |  | X |  |  |  | X |  |  | X**^2^** |  |  |  | X |  |  | X**^3^** |  |  |  | X | X | X |
| Concurrent medications**^5^** | X |  | X |  |  | X |  |  |  | X |  |  | X**^2^** |  |  |  | X |  |  | X**^3^** |  |  |  | X | X | X |
| Physical exam**^5^** | X |  | X |  |  | X |  |  |  | X |  |  | X**^2^** |  |  |  | X |  |  | X**^3^** |  |  |  | X | X | X |
| Weight**^5^** | X |  | X |  |  |  |  |  |  | X |  |  |  |  |  |  | X |  |  |  |  |  |  | X | X | X |
| ECOG performance status | X |  | X |  |  | X^2^ |  |  |  | X |  |  | X**^2^** |  |  |  | X |  |  | X**^3^** |  |  |  | X | X | X |
| ECG**^6^** | X |  | X |  |  |  |  |  |  |  |  |  |  |  |  |  |  |  |  |  |  |  |  | X |  | X |
| Vital signs | X |  | X**^7^** | | | | | | | | | | | | | | | | | | | | |  |  |  |
| CT chest abdomen pelvis | X**^8^** |  |  | | | | | | | | | | | | | | | | | | | | |  |  | X^10^ |
| Collection of archival biopsy |  |  |  | | | | | | | | | | | | | | | | | | | | |  |  | X^11^ |

^1^ Screening assessments including: pregnancy test (to be repeated for WOCBP 4 weekly during treatment), height and medical history, see section 5.3

^2^ Dose schedules -2, -1, 2, 3, 5 & 6 only

^3^ Dose schedules -1, 3 & 6 only

^4^ Screening and within 24 hours prior to dosing with M6620 (Berzosertib): Haemoglobin, ANC, lymphocytes, WBC, Platelets, Bilirubin, ALP, AST or ALT, urea, Serum creatinine, eGFR, K^+^, Na^+^

^5^ +/- 24 hours, must be prior to dosing with M6620 (Berzosertib); day 4 all patients (excluding weight day 4)

^6^ ECG to be carried out at screening, baseline (pre-M6620 (Berzosertib)) week 4 and week 12 and if clinically indicated on treatment

^7^ Per clinical requirement

^8^ Screening CT only needs to be done if diagnostic CT carried out > 42 days prior to start of treatment

^9^ Week 4 follow-up should be 1 week after last dose/fraction (+7 days)

^10^ CT Scan can be done up to 7 days prior to the Week 12 visit

^11^ Optional

## SUMMARY SCHEDULE OF EVENTS STAGE A2

|  | | | Cycle 1* | | | Cycle 2 | | | Cycle 3 | | | Cycle 4 | | | Cycles 5-6**^10^** | Follow up**^11^** | |
| --- | --- | --- | --- | --- | --- | --- | --- | --- | --- | --- | --- | --- | --- | --- | --- | --- | --- |
|  | Screening | Post Registration | Week 1 | Week 2 | Week 3 | Week 4 | Week 5 | Week 6 | Week 7 | Week 8 | Week 9 | Week 10 | Week 11 | Week 12 |  | 2 weeks post EOT | 8 weeks post EOT |
| Informed Consent | X |  |  |  |  |  |  |  |  |  |  |  |  |  | Repeat assessments done for cycles 3-4 in cycles 5-6 (if applicable) |  |  |
| Screening assessments**^1^** | X |  |  |  |  |  |  |  |  |  |  |  |  |  |  |  |  |
| M6620 (Berzosertib) schedule assignment |  | X |  |  |  |  |  |  |  |  |  |  |  |  |  |  |  |
| Capecitabine**^2^** |  |  | X | X | X | X | X | X | X | X | X | X | X | X |  |  |  |
| Cisplatin**^3^** |  |  | X^3^ |  |  | X^3^ |  |  | X^3^ |  |  | X^3^ |  |  |  |  |  |
| M6620 (Berzosertib)**^4^** |  |  | X^4^ | X^4^ | X^4^ | X^4^ | X^4^ | X^4^ | X^4^ | X^4^ | X^4^ | X^4^ | X^4^ | X^4^ |  |  |  |
| Haematology/Biochemistry**^5^** | X |  | X | X | X | X | X | X | X | X | X | X | X | X |  | X | X |
| Coagulation | X |  | X |  |  |  |  |  |  |  |  |  |  |  |  | X |  |
| ECOG performance status^6^ | X |  | X | X | X | X | X | X | X | X | X | X | X | X |  | X | X |
| Physical Exam**^6^** | X |  | X | X | X | X | X | X | X | X | X | X | X | X |  | X | X |
| Weight**^6^** | X |  | X | X | X | X | X | X | X | X | X | X | X | X |  | X | X |
| Vital signs | X |  | X**^8^** | | | | | | | | | | | |  |  |  |
| ECG**^7^** | X |  | X |  |  | X |  |  | X |  |  | X |  |  |  |  |  |
| Audiogram monitoring^13^ |  | X |  |  |  | X |  |  |  |  |  |  |  |  |  |  |  |
| Concurrent medications**^6^** | X |  | X | X | X | X | X | X | X | X | X | X | X | X |  | X | X |
| DLT Assessment**^6^** |  |  | X | X | X | X |  |  |  |  |  |  |  |  |  |  |  |
| AE Assessment**^6^** |  |  | X | X | X | X | X | X | X | X | X | X | X | X |  | X | X |
| CT chest abdomen pelvis**^9^** | X |  |  |  |  |  |  | X |  |  |  |  |  | X |  |  | X |

**^1^** Screening assessments including: pregnancy test (to be repeated for WOCBP 4 weekly during treatment), height and medical history, see section 5.3

**^2^** Capecitabine taken bd weeks 1 to 18

^3^ Cisplatin given on day 1 (Mon)

^4^ M6620 (Berzosertib) given on day 2 only (Tues) dose schedules 1 & 3; M6620 (Berzosertib) given on day 2 & day 5 (Tues/Fri) dose schedules 2 & 4

^5^ Check Haem/Biochem within 72 hours prior to cisplatin and 24 hours preceding M6620 (Berzosertib) (only one sample needs to be taken if it satisfies both Cisplatin and M6620 (Berzosertib) requirements): Hb, ANC, lymphocytes, WBC, Platelets, Bilirubin, ALP, AST or ALT, Serum creatinine, Urea, K^+^, Na^+^, eGFR, Ca, Mg, Phosphate

M6620 (Berzosertib)M6620 (Berzosertib)^6^ Assessment done within 24 hours prior to M6620 (Berzosertib) dosing (weight once weekly)

^7^ECG to be carried out at screening, pre-treatment and once per cycle

^8^ Per clinical requirement

^9^ CT chest abdomen pelvis in weeks 6, 12, 18 and at 8 weeks post EOT. Screening CT only needs to be done if staging CT was carried out > 35 days prior to start of treatment.

^10^ Patients can continue with treatment after 4 cycles if CT shows no progression and at the discretion of the PI.

^11^ Two week follow-up should be 2 weeks after last dose (+/-7 days); 8 week follow-up should be 8 weeks after last dose (+/- 2 weeks)

^12^ If required. *Each cycle lasts 3 weeks

## SUMMARY SCHEDULE OF EVENTS STAGE B

|  | | | **Cycle 1*** | | | **Cycle 2** | | | **Cycle 3** | | | | | | | | | | | | | | | **Cycle 4** | | | | | | | | | | **Follow up** | | |
| --- | --- | --- | --- | --- | --- | --- | --- | --- | --- | --- | --- | --- | --- | --- | --- | --- | --- | --- | --- | --- | --- | --- | --- | --- | --- | --- | --- | --- | --- | --- | --- | --- | --- | --- | --- | --- |
|  | Screening | Baseline | Week 1 | Week 2 | Week 3 | Week 4 | Week 5 | Week 6 | Week 7 | | | | | Week 8 | | | | | Week 9 | | | | | Week 10 | | | | | Week 11 | | | | | Week 12^13^ | Week 18 | Week 24 |
|  |  |  |  |  |  |  |  |  | 1 | 2 | 3 | 4 | 5 | 8 | 9 | 10 | 11 | 12 | 15 | 16 | 17 | 18 | 19 | 22 | 23 | 24 | 25 | 26 | 29 | 30 | 31 | 32 | 33 |  |  |  |
|  |  |  |  |  |  |  |  |  | M | T | W | Th | F | M | T | W | Th | F | M | T | W | Th | F | M | T | W | Th | F | M | T | W | Th | F |  |  |  |
| Informed Consent | X |  |  |  |  |  |  |  |  |  |  |  |  |  |  |  |  |  |  |  |  |  |  |  |  |  |  |  |  |  |  |  |  |  |  |  |
| Screening assessments**^1^** | X |  |  |  |  |  |  |  |  |  |  |  |  |  |  |  |  |  |  |  |  |  |  |  |  |  |  |  |  |  |  |  |  |  |  |  |
| M6620 (Berzosertib) schedule assignment |  |  |  |  |  |  |  | X |  |  |  |  |  |  |  |  |  |  |  |  |  |  |  |  |  |  |  |  |  |  |  |  |  |  |  |  |
| Radiotherapy planning |  |  |  | X | | |  |  |  |  |  |  |  |  |  |  |  |  |  |  |  |  |  |  |  |  |  |  |  |  |  |  |  |  |  |  |
| IMRT (50GY in 25#) |  |  |  |  |  |  |  |  | X | X | X | X | X | X | X | X | X | X | X | X | X | X | X | X | X | X | X | X | X | X | X | X | X |  |  |  |
| Capecitabine**^2^** |  |  | X | X | X | X | X | X | X | X | X | X | X | X | X | X | X | X | X | X | X | X | X | X | X | X | X | X | X | X | X | X | X |  |  |  |
| Cisplatin (Day 1) |  |  | D1 |  |  | D1 |  |  | X |  |  |  |  |  |  |  |  |  |  |  |  |  |  | X |  |  |  |  |  |  |  |  |  |  |  |  |
| M6620 (Berzosertib) (Day 2) |  |  | D2 |  |  | D2 |  |  |  | X^4^ |  |  | X^5^ |  | X |  |  | X^6^ |  | X |  |  | X^8^ |  | X |  |  | X^5^ |  | X |  |  | X^7^ |  |  |  |
| Haematology/Biochemistry**^3^** | X |  | X |  |  | X |  |  | X |  |  | X^5^ |  | X |  |  | X^6^ |  | X |  |  | X^8^ |  | X |  |  | X^5^ |  | X |  |  | X^7^ |  | X | X |  |
| Coagulation | X |  | X |  |  |  |  |  |  |  |  |  |  |  |  |  |  |  |  |  |  |  |  |  |  |  |  |  |  |  |  |  |  | X |  |  |
| ECOG performance status | X |  | X | X |  | X | X |  | X |  |  | X^5^ |  | X |  |  | X^6^ |  | X |  |  | X^8^ |  | X |  |  | X^5^ |  | X |  |  | X^7^ |  | X | X | X |
| Physical Exam**^9^** | X |  | X | X |  | X | X |  | X |  |  | X^5^ |  | X |  |  | X^6^ |  | X |  |  | X^8^ |  | X |  |  | X^5^ |  | X |  |  | X^7^ |  | X | X | X |
| Weight**^9^** | X |  | X |  |  | X |  |  | X |  |  |  |  | X |  |  |  |  | X |  |  |  |  | X |  |  |  |  | X |  |  |  |  | X | X | X |
| Vital signs**^9^** | X |  | X | X |  | X | X |  | X |  |  | X^5^ |  | X |  |  | X^6^ |  | X |  |  | X^8^ |  | X |  |  | X^5^ |  | X |  |  | X^7^ |  | X | X | X |
| ECG^10^ | X |  | X |  |  | X |  |  | X |  |  |  |  |  |  |  |  |  |  |  |  |  |  | X |  |  |  |  |  |  |  |  |  |  |  | X |
| Audiogram monitoring^14^ |  | X |  |  |  | X |  |  |  |  |  |  |  |  |  |  |  |  |  |  |  |  |  |  |  |  |  |  |  |  |  |  |  |  |  |  |
| Concurrent medications**^9^** | X |  | X | X |  | X | X |  | X |  |  | X^5^ |  | X |  |  | X^6^ |  | X |  |  | X^8^ |  | X |  |  | X^5^ |  | X |  |  | X^7^ |  | X | X | X |
| DLT Assessment**^9^** |  |  |  | X |  | X | X |  | X |  |  | X^5^ |  | X |  |  | X^6^ |  | X |  |  | X^8^ |  | X |  |  | X^5^ |  | X |  |  | X^7^ |  | X | X | X |
| AE Assessment**^9^** |  |  | X | X |  | X | X |  | X |  |  | X^5^ |  | X |  |  | X^6^ |  | X |  |  | X^8^ |  | X |  |  | X^5^ |  | X |  |  | X^7^ |  | X | X | X |
| Mellow Score**^9^** |  |  | X | X |  | X | X |  | X |  |  | X^5^ |  | X |  |  | X^6^ |  | X |  |  | X^8^ |  | X |  |  | X^5^ |  | X |  |  | X^7^ |  | X | X | X |
| Research blood sample |  | X |  |  |  |  |  |  |  |  | X^11^ | | |  |  |  |  |  |  |  |  |  |  |  |  |  |  |  |  |  |  |  |  | X |  |  |
| Research biopsy |  |  |  |  |  |  |  |  |  |  | X^11^ | | |  |  |  |  |  |  |  |  |  |  |  |  |  |  |  |  |  |  |  |  |  |  | X |
| CT | X^12^ |  | | | | | | | | | | | | | | | | | | | | | | | | | | | | | | | | | | X |

**^1^** Screening assessments including: pregnancy test (to be repeated for WOCBP 4 weekly during treatment), height, medical history, Echo/MUGA and Lung function test, see section 5.3

**^2^** Capecitabine taken bd weeks 1 to 6; capecitabine taken bd Mon to Fri weeks 7 to 11

^3^ Check Haem/Biochem within 72 hours prior to cisplatin and 24 hours preceding M6620 (Berzosertib) (only one sample needs to be taken if it satisfies both Cisplatin and M6620 (Berzosertib) requirements): Hb, ANC, lymphocytes, WBC, Platelets, Bilirubin, ALP, AST or ALT, Serum creatinine, Urea, K^+^, Na^+^, eGFR, Ca, Mg, Phosphate

^4^ Except schedule 1 **^5^** M6620 (Berzosertib) schedules 3, 4, 5 & 6 only **^6^** M6620 (Berzosertib) schedules 4, 5 & 6 only **^7^** M6620 (Berzosertib) schedules 5 & 6 only ^8^ M6620 (Berzosertib) schedule 6 only

**^9^** Assessment done within 24 hours prior to M6620 (Berzosertib) dosing; and in weeks 2 & 5 of induction chemotherapy on day 1 or day 2 (excluding weight weeks 2 & 5)

^10^ ECG in cycle 1 to be carried out pre-treatment

^11^ On treatment biopsy/blood sample should be done from radiotherapy fraction 3 to fraction 5 (but up to fraction 7 is permitted if required)

^12^ Screening CT only needs to be done if diagnostic CT carried out > 42 days prior to start of treatment

^13^ Week 12 follow up to be done one week after last dose/fraction (+7 days) ^14^If required. *Each cycle lasts 3 weeks

## Study Flow Charts

**Stage A1**

Week

1

2

3

4

9

12

Follow

up visit

Follow

up visit

Screening + RT planning

Duration of daily RT + intermittent M6620 (Berzosertib)

End of study visit

Clinical review and assessment of toxicity before each M6620 (Berzosertib) administration, and at follow up

**Stage A2**

Week

9 10 11 12 13 14 15 16 17 18

3

4

5

6

8

2

20

7

1

26

3 weekly cycles of chemotherapy^1^ + intermittent M6620 (Berzosertib) (once or twice a week)

Screening

Follow up visit

End of study visit

**^1^** Daily Capecitabine; Cisplatin on day 1 of 21 day cycle

Clinical review and assessment of toxicity before each M6620 (Berzosertib) administration, and at follow up

Cisplatin IV infusion

**Stage B**

1

18

24

Screening

Induction Chemotherapy**^1^** Chemoradiotherapy**^2^**

M6620 (Berzosertib) dosing**^3^**

Follow up visit

Follow up visit

End of study visit

Cisplatin IV infusion

Clinical review and assessment of toxicity pre M6620 (Berzosertib) administration,

and at follow up

Research endoscopy Research Blood Sample

2

3

4

5

6

7

8

9

10

111

122

Week

M6620 (Berzosertib) schedule assignment

**Treatment**

**^1^** Daily Capecitabine; Cisplatin on day 1 of 21 day cycle

**^2^** Capecitabine (Mon to Fri) and RT (Mon to Fri) for 5 weeks; Cisplatin continues on day 1

of 21 day cycle

**^3^** On day 2 of 21 day cycle during induction chemotherapy. Assignment of intermittent

schedule in week 6 and commencement of intermittent schedule in week 7

# Abbreviations

| 4DCT | 4D computed tomography |
| --- | --- |
| 4DCECT | 4D contrast enhanced computed tomography |
| ACA | Adenocarcinoma |
| AE | Adverse Event |
| BOI | Beginning of infusion |
| CR | Complete Response |
| CRT | Chemoradiotherapy |
| D | Day |
| dCRT | Definitive Chemoradiotherapy |
| DLT | Dose Limiting Toxicity |
| EOI | End of infusion |
| FDG-PET | Fluorodeoxyglucose Positron Emission Tomography |
| GTV | Gross Tumour Volume |
| IB | Investigator Brochure |
| ICRU 62 | International Commission on Radiation Units Report 62 |
| IHC | Immunohistochemistry |
| IMP | Investigational Medicinal Product |
| IMRT | Intensity-Modulated Radiation Therapy |
| mOS | Mean Overall Survival |
| MA | Marketing Authorisation |
| MTD | Maximum Tolerated Dose |
| OAC | Oesophageal Adenocarcinoma |
| OS | Overall Survival |
| PI | Principal Investigator |
| RSI | Reference Safety Information |
| RT | Radiotherapy |
| RTTQA | Radiotherapy Trials Quality Assurance |
| SAR | Serious Adverse Reaction |
| SCC | Squamous Cell Carcinoma |
| SmPC | Summary of Product Characteristics |
| LPLV | last visit of the last patient undergoing the trial |
| SUSAR | Suspected Unexpected Serious Adverse Drug Reaction |
| TiTE-CRM | Time to Event Continual Reassessment Method |
|  |  |
|  |  |

# Introduction

## Background

Oesophageal cancer has been identified by CRUK as a cancer of unmet need, because of persistent low 5-year survival (13%) and lack of research in this disease. The incidence of oesophageal cancer has risen in recent decades, coinciding with a shift in histology and primary tumour location (www.cancerresearchuk.org/cancer-info/cancerstats/types/oesophagus/). In 2010, 8500 people were diagnosed with oesophageal cancer in the UK and in 2011 there were 7600 deaths, making it the 4th most common cause of death in males. In the UK it accounts for around 5% of all cancer deaths (Cancer Research UK, n.d.). Surgery has been the cornerstone of curative treatment of oesophageal adenocarcinoma (OAC) but is only appropriate for 10-20% of the patient population as at presentation the majority of patients are unsuitable for surgery and have locally advanced or metastatic disease irrespective of histologic type. Surgery for squamous cell carcinoma (SCC) is used less often; the proximal nature of the disease making reconstructive surgery more challenging and patients often being less fit as a result of co-existing cardio-pulmonary disease due to the common aetiology of the diseases.

**Radical Setting**

Definitive chemo-radiotherapy (dCRT) is usually offered to those patients who have non-metastatic oesophageal cancer who are unsuitable for surgery but this modality has been increasingly considered a standard of care for patients with operable SCC [15]. The recently reported SCOPE 1 trial, a CRUK-funded trial which investigated the addition of Cetuximab to standard cisplatin/fluoropyrimidine based dCRT [12], reported unprecedented good outcomes in the standard dCRT arm with a mOS of 25.4 mo and 2-yr OS of 56%. This study demonstrated that with a detailed protocol and a robust radiotherapy trials quality assurance (RTTQA) programme, high quality dCRT can be delivered throughout the UK and lead to outcomes equivalent to that seen in published surgical series. The dCRT outcome was comparable to surgical outcomes despite the patient population in this trial having a relatively poor prognosis. Unfortunately, other chemotherapy regimens have not shown an improved survival over the Cisplatin Fluorouracil combination. There was no increase in median PFS after Oxaliplatin and Fluorouracil (FOLFOX) dCRT when compared with Cisplatin Fluorouracil dCRT 9.7 months (95% CI 8.1–14.5) vs 9.4 months (8.1–10.6) or median OS 20.2 months (95% CI 14.7–25.6),vs 17.5 months (13.9–19.4) respectively[2] highlighting the need for better treatment in this patient group.

A recent study in oesophageal cancer reported patterns of failure after dCRT, assessed by FDG-PET scan in 239 patients [3]. With a median follow-up of 52.6 months, 119 patients (50%) had relapsed locally, 90% of which were within the Gross Tumour Volume (GTV). Having a failure within the GTV (as opposed to all other failure patterns plus patients without failure) influenced OS as well; the median OS time for patients with GTV failure was 23.3 months (95% CI, 20.00-31.32) versus 31.6 months for those with no GTV failure (95% CI, 24.31-not reached; p< .0009). A similar study by Button et al reviewed the patterns of relapse in 145 patients treated with dCRT [4]. Of the 85 (60%) patients who had evidence of relapse after a median on 18 months, 55 had relapse within the irradiated field, 13 relapsed with metastatic disease and 14 had a combination of local and distant disease. Another study assessed patterns of relapse in 274 patients treated in non-randomised trials by TROG between 1985 and 1999[5]. Local failure was observed in 42.3% and distant failure in isolation occurred in 18.1%. In the subgroup with least favourable survival, adenocarcinoma of the lower third of the oesophagus local failure rate (51.5%) dominated over distant failure (36.1%). Taken together, these studies suggest that both local and systemic failures are competing risk factors, and integrating a new agent with the standard dCRT schedule is most likely to succeed if this can enhance the activity of both cisplatin (systemic component) and radiotherapy (local component).

In SCOPE 1[1] patients who were failure free at 24 weeks (76% in the standard arm) had significantly better median overall survival than did those who were not failure free (26.7 mo [24.5–42.7]vs. 8.3 mo [95% CI 6.7–12.5] respectively. The RTOG 0436 study evaluated whether the addition of cetuximab to paclitaxel, cisplatin and RT improved overall survival in patients with oesophageal cancer who are treated without surgery [6]. The study (comparable to SCOPE1) failed to show that adding cetuximab improved survival. However, clinical assessments with endoscopy at 6-8 weeks after completion of therapy were undertaken in all evaluable patients and an analysis of responders (CR versus non CR) were predictive of survival HR=2.2(1.59-2.83) [6].

**Palliative setting**

dCRT treatment is toxic with a death rate of 2% due to significant life threatening toxicity. Cisplatin is nephrotoxic, and fluoropyrimidines can precipitate acute coronary syndrome therefore CRT is only offered to good performance status (PS) patients with adequate renal function and no significant ischaemic heart disease. Patients unsuitable for CRT may be treated with radiotherapy alone as a non-invasive means of palliating dysphagia and comprise around half of patients referred for RT based treatment.

TROG 03.01, a multinational phase III study in advanced oesophageal cancer ([7](#_ENREF_7)) comparing palliation of dysphagia and quality of life in patients treated with radiotherapy or chemoradiotherapy (CMT) randomised 220 patients to receive a course of palliative RT [35 Gy in 15 fractions, (n=115) or 30 Gy in 10 fractions (n=105)], or concomitant CRT with Cisplatin and 5FU (D1-4) (n=111). The primary endpoint was the proportion of patients with improved dysphagia measured at week 9 and maintained until week 13. RT alone showed a dysphagia response (at any point) of 68% similar to CMT 74%, (p=0.343). The primary endpoint of dysphagia improvement was achieved in 41% with RT and 47% with CMT (p=0.4163). There was increased toxicity in patients receiving CMT, (nausea (p=0.0019) and vomiting (p=0.0072)). Median survival was 210 days for CRT, 203 days for RT. Although the results of the trial showed equally poor survival in both arms, there were some patients (n=21, 10%) still alive at 2 years post treatment indicating that this is a group of patients who should not be denied active cancer treatment.

**Summary of design**

This phase I study will test the combination of a novel ATR inhibitor (M6620 (Berzosertib)) with chemoradiotherapy in oesophageal cancer. In the first two cohorts (Stage A1 and A2), we will investigate the safety of combining M6620 (Berzosertib) separately with [1] palliative radiotherapy (RT) for oesophageal cancer (Stage A1) and [2] with cisplatin/capecitabine chemotherapy in patients with advanced inoperable and metastatic solid tumours (Stage A2). In Stage A1, M6620 (Berzosertib) will be given in combination with high dose palliative RT treatment, aiming to deliver M6620 (Berzosertib) twice weekly during RT escalating to a dose of 240mg/m^2^. A palliative chemotherapy cohort (Stage A2) will open to recruitment simultaneously where M6620 (Berzosertib) will be given in combination with cisplatin/capecitabine chemotherapy, aiming to deliver M6620 (Berzosertib) twice weekly escalating to a dose of 140mg/m^2^ twice weekly. When adequate toxicity and follow-up information to suggest the combinations are tolerable has accumulated, the ATR inhibitor will be tested in the definitive setting (Stage B) in combination with cisplatin/capecitabine and radical RT to identify the Maximum Tolerated Dose (MTD). The MTD found in this study will be taken forward in future phase II studies.

In the palliative setting, we aim to find the schedule associated with no more than 25% Dose Limiting Toxicities (DLTs) in stage A1 on the basis that palliative oesophageal radiotherapy causes approximately 20% grade 3 and 4 toxicity and 30% Dose Limiting Toxicities (DLTs) in stage A2 are derived from capecitabine/cisplatin used in the radical setting (SCOPE1 study [12]).

In the radical setting, we aim to find the schedule associated with no more than 45% DLTs on the basis that conventional oesophageal chemoradiation causes a grade 3 and 4 toxic event rate of 28% haematological toxicity and 63% non-haematological toxicity of which 34% is gastrointestinal as reported in the standard arm of SCOPE1 study ([12](#_ENREF_12)). Comparable toxicity rates were described in the standard arm of the PRODIGE5/ACCORD17 study ([13](#_ENREF_13)): grade 3 and 4 neutropenia 29% and grade 3 and 4 dysphagia and oesophagitis 33%.

The trial will find the best optimal dose and dosing schedule using the TiTE-CRM (Time To Event Continual Reassessment Method). The CRM is a model based method for finding the MTD. It assumes that toxicity increases monotonically with increasing dose, and that efficacy also increases with increasing dose. The aim will be to find the dose that causes a DLT with the above specified target toxicity levels. TiTE-CRM is a modified CRM that accounts for the time to event of late onset toxicities. The advantages of a TiTE-CRM are that all current critical toxicity summaries are used when deciding which dose to give the next patient and it is not necessary for a patient to complete the full observation period before consenting the next patient. This results in a better estimation of the MTD and shorter study duration respectively.

## Investigational Medicinal Product(s) used in the study

**M6620 (Berzosertib)**

M6620 (Berzosertib) is an unlicensed small molecule ATR inhibitor which can be used in combination with DNA damaging agents. In pre-clinical models it has substantial activity when given with DNA damaging drugs or ionising radiation. The clinical agent (M6620 (Berzosertib)) is currently studied in a phase I trial in Oxford and other centres in combination with gemcitabine, cisplatin, gemcitabine/cisplatin and cisplatin/etoposide (see section 1.4)

**Cisplatin**

Cisplatin is a platinum based chemotherapy drug licensed to treat a number of different types of cancer (see SmPC for more details).

**Capecitabine**

Capecitabine is a chemotherapy drug licensed to treat a number of different types of cancer, it is a non-cytotoxic pre-cursor of the cytotoxic 5-fluourouracil (see SmPC for more details).

## Pre-clinical rationale

DNA damaging agents (e.g. cisplatin and RT) are key treatments for many solid tumours including oesophageal cancer. Tumours can be resistant to current DNA-damaging based therapies due to the existence of an effective DNA Damage Response (DDR). The DDR consists of a series of molecular events that allow repair of damaged DNA and promote cell survival. ATM (Ataxia Telangiectasia Mutated) and ATR (ATM-and Rad3-related), members of phosphoinositol3-kinase like kinase family (PIKKs), are key components of the DDR. During normal DNA replication, ATR is recruited to stalled replication forks (replication stress) that can progress to DNA double strand breaks if unprotected. The recruitment and activation of ATR leads to cell cycle arrest in S phase whilst DNA is repaired; otherwise nuclear fragmentation occurs and apoptosis is initiated. Therefore, blocking ATR in an environment where replication stress is elevated as a result of treatment with radiotherapy or chemotherapy should improve killing of cancer cells. Consistently, it has been demonstrated that radiation and Cisplatin are more efficacious in tumour cells where kinase dead ATR has been expressed ([1](#_ENREF_1), [2](#_ENREF_2)).

M6620 (Berzosertib) is a potent inhibitor of ATR (inhibition constant [Ki] <300 pM) that blocks ATR activity in cells, with a concentration resulting in 50% maximal inhibition (IC_50_) of 20 nM. ATR inhibition enhances the cytotoxic effect of DNA damaging drugs and IR in many cancer cell lines and primary human tumors. In contrast, normal cells tolerate ATR inhibition since they can activate compensatory DDR signaling via the ATM pathway. In xenograft models, M6620 (Berzosertib) markedly enhances the anticancer activity of numerous DNA damaging drugs and IR, often substantially delaying or completely halting tumor progression and promoting tumor regression. Dose range finding studies in mice (with gemcitabine and cisplatin) showed that maximal activity was observed when M6620 (Berzosertib) was administered intravenously at a dose of 20 mg/kg/week, given as a single dose or as two 10‑mg/kg doses 3 days apart. Dose‑responsive biomarker effects, which correlate with efficacy, support ATR inhibition as the primary mechanism of action.

Consistent with the compensatory role the ATM/p53 pathway plays in response to ATR inhibition in normal cells, defects in this pathway result in increased cell sensitivity to ATR inhibition. In isogenic cell studies it has been shown that loss of ATM itself or one of its principle substrates, p53, can markedly increase cell sensitivity to ATR inhibition. Similarly, in a large panel of 119 genetically-diverse cancer cell lines, *TP53* mutational status was shown to correlate with response to ATR inhibition in combination with DNA damaging agents.

Pires *et al* ([10](#_ENREF_10)) showed that VE-821 also inhibited ATR-mediated signalling in response to the replication arrest induced by severe hypoxia and that ATR inhibition consistently sensitised tumour cell lines to radiation across a range of oxygen tensions. In addition, it was shown for the first time that treatment with the ATR inhibitor led to a decrease in HIF-1-mediated signalling, suggesting that it could also inhibit the biological consequences of tumour hypoxia such as increased invasion, metastasis and angiogenesis.

Fokas *et al* ([11](#_ENREF_11)) demonstrated radio-sensitisation and chemosensitisation to gemcitabine using the ATR inhibitor VE-822 in p53 and KRAS mutant pancreatic ductal adenocarcinoma (PDAC) *in vitro* and *in vivo* (VE-822 is another pre-clinical ATR inhibitor chemically identical to M6620 (Berzosertib)). The selectivity of VE-822 was initially demonstrated through selective reduction in CHK1 phosphorylation without inhibition of ATM or DNA-PK signalling pathways. In xenograft experiments, the activity was profound, to the extent that the combination of RT with VE-822 prevented MiaPaCa-2 tumour regrowth in some mice. Importantly, VE-822 did not increase normal cell radiosensitivity and chemosensitivity *in vitro*, similar to the VE-821 studies described previously.

ATR inhibition has not been previously tested specifically on oesophageal cancer cell lines or xenografts. We have recently demonstrated chemosensitisation (cisplatin) and radiosensitisation of ACA and SCC cell lines using VE-822. These data show that the addition of the ATR inhibitor (VE-822) increases sensitivity to radiation as well as cisplatin in 3 oesophageal cell lines (OE21, FLO-1 and OE33) both under normoxic and hypoxic conditions (<0.1% O_2_) (Hammond, unpublished data).

## Clinical rationale

There is strong scientific rationale for combining ATR inhibitors with DNA damaging agents such as radiation and cisplatin. In particular, ATR inhibition has been shown to be cytotoxic to tumour cells with an impaired DNA damage response (DDR), such as those with deficiency in the ATM- or p53 pathway [7]. The high incidence of p53 mutations (~89.9% in SCC of the oesophagus and ~72% in ACA) [8, 9] and the fact that cisplatin and radiation are key therapeutics, makes oesophageal cancer an attractive tumour type to test the activity of an ATR inhibitor [8-10]. Given the reported synthetic lethal relationship between ATM and ATR, it is likely that ATR inhibition in an ATM- or p53- deficient background will offer a specific and effective way of targeting OAC and SCC of the oesophagus, and enhance the current standard of care.

The in vitro and in vivo studies mentioned above, have shown that M6620 (Berzosertib) can enhance sensitivity of cancer cells to chemotherapy and radiotherapy without enhancing radiosensitivity in normal tissue [7, 11]. This tumour selectivity suggests there will be little or no enhancement of radiation toxicity and therefore, is likely to allow delivery of full doses of chemotherapy and radiotherapy. In contrast, recently reported studies in gastro-oesophageal cancer, toxicity due to addition of novel agents resulted in reduction of dose intensity of standard treatment and inferior survival in the experimental arms[1, 12] underlining the importance of this lack of toxicity in normal tissues.

In an ongoing study (VX12-970-001), M6620 (Berzosertib) is being dosed in combination gemcitabine and in combination with cisplatin to determine the MTD of M6620 (Berzosertib) in combination with these agents. To date, 140 mg/m2 of M6620 (Berzosertib) in combination with 75 mg/m2 of cisplatin was tolerated. Also, 210 mg/m2 of M6620 (Berzosertib) in combination with 1000 mg/m2 of gemcitabine was tolerated and is the recommended phase II dose. In another ongoing study (VX13-970-002), M6620 (Berzosertib) at 90 mg/m2 in combination with AUC 5 of carboplatin was tolerated. Also, evaluation of on-target tumor biopsies at these doses demonstrated target engagement as measured by ATR-mediated phosphorylation of Chk1 (P-Chk1).

The trial will be divided into 2 stages, stage A and Stage B. Stage A will consist of 2 parts, part A1 will explore the combination of M6620 (Berzosertib) plus radiotherapy and Stage A2 will explore the combination of M6620 (Berzosertib) plus chemotherapy in the palliative setting. Stage B, will explore the combination of all 3, M6620 (Berzosertib) plus chemoradiotherapy in the radical setting. In Stage A1 of the study M6620 (Berzosertib) will be combined with radiotherapy for the first time and the starting dose will be 140mg/m^2^ M6620 (Berzosertib), which has been well-tolerated. Intravenous (IV) administration was shown to be better tolerated than oral administration in dogs and will be used in the study. We have chosen to administer M6620 (Berzosertib) with daily palliative radiotherapy in Stage A1 to study specific interaction of M6620 (Berzosertib) with radiotherapy which is the DNA damaging agent at this stage. In Stage A2 of the study M6620 (Berzosertib) will be combined with Cisplatin and Capecitabine combination chemotherapy for the first time and the starting dose will be 90mg/m^2^ M6620 (Berzosertib). We have chosen to administer M6620 (Berzosertib) after chemotherapy to explore possible additional interactions with cisplatin which binds with DNA to form intrastrand crosslinks and adducts that cause changes in the conformation of the DNA and affects DNA replication fluoropyrimidines whose primary mechanism of action is the inhibition of thymidylate synthase. Stage A1 and A2 will give an indication of toxicity profile before administration with radiotherapy, Capecitabine and Cisplatin during chemoradiotherapy. Data from the VERTEX study VX12-970-001 indicates maximum benefit from the chemotherapy and M6620 (Berzosertib) treatment combination comes with administration of M6620 (Berzosertib) between 14 and 36 hours post administration of DNA damaging agent (VERTEX, unpublished results). Therefore we propose to administer M6620 (Berzosertib) 24 hours post cisplatin infusion.

# TRial design

This will be a single arm, open-label, phase I dose escalation trial using the Time-To-Event Continual Reassessment Method (TiTE-CRM) to find the optimal treatment schedule. The TiTE-CRM method uses critical toxicity summaries of accumulated patient data from all participants treated with at least one dose of the IMP within the corresponding trial stage and for whom up-to-date data has been provided (trial unit will endeavour to ensure contemporaneous data is received for all participants) and it is not necessary for a patient to complete the full observation period before consenting the next patient. This results in a better estimation of the MTD and shorter study duration respectively and is particularly useful in trials involving radiotherapy where the toxicity follow-up phase is longer.

The trial design ensures no treatment schedule skipping and the treatment schedule assigned will be that estimated to be closest to but not above the MTD. However, if the lowest schedule is estimated to be above the MTD we will keep assigning the lowest schedule until we are certain it is too toxic, at which point the trial may start again using a lower dose of drug. When escalating, the treatment schedule cannot skip an untried dose but there will be no restriction on treatment schedule de-escalation. Each escalation decision will be made by the TMG based on the recommendation from the TiTE-CRM model and the accumulated experience of the recommended schedule. If the TMG is unable to reach a decision or a stopping rule has been met the Safety Review Committee (SRC) will meet.

The trial consists of three stages A1, A2 and B which are described in the following sections. Stages A1 and A2 will run concurrently and will inform the starting dose of M6620 (Berzosertib) for Stage B.

## Stage A1

The aim is to find the M6620 (Berzosertib) treatment schedule when combined with radiotherapy that is associated with no more than 25% dose limiting toxicity rate on the basis that palliative oesophageal radiotherapy is associated with approximately 15-20% grade 3/4 toxicity. Six treatment schedules are proposed. Each schedule comprises a specific combination of dose and dosing frequency. There are two possible M6620 (Berzosertib) doses and three dosing frequencies (see section 8.1). The radiation dose remains consistent across all treatment schedules.

The treatment involves 3 weeks of daily radiotherapy and M6620 (Berzosertib) at a pre-determined frequency dependent on the treatment schedule allocated to the individual patient. The follow-up of a further 6 weeks provides a DLT observation window of a total of 9 weeks. An initial cohort of three patients will receive the starting schedule (lowest dosing frequency) at the starting dose, 140mg/m^2^. The fourth patient will not be recruited until all three patients have been followed for the minimum of 9 weeks from the start of radiotherapy or the occurrence of a DLT.

Subsequently, all eligible patients will be continuously recruited and the TiTE-CRM will be used to assign their treatment schedule. To ensure enough information is accumulated to inform the assignment of the treatment schedule to the subsequent patient, recruitment will be managed through allocation of treatment slots (see section 4.4 for further details).

**2.1.1. Stage A1 stopping rules**

Stage A1 will pause for safety if, at any point in the trial, there is sufficient evidence to suggest that schedule 1 is too toxic. More specifically, we will consider schedule 1 to be too toxic if, given all the available data, there is a high probability that the DLT rate is greater than the target toxicity level of 0.25. If all 3 patients in the first cohort have DLTs then schedule 1 is too toxic and the trial will be re-started. At this point, three extra schedules will be introduced at 90mg/m^2^ and varying dosing frequencies, namely (schedule -3, -2 and -1). Once the trial is restarted, the lowest schedule, schedule -3, will be explored first. There will then be 9 treatment schedules to explore (the original 6 plus the 3 dosing frequencies at the lower dose). If the first 3 patients recruited to schedule -3 experience DLTs then the trial will stop. If schedule 1 is found to be too toxic later in the trial when more than 3 patients have been recruited, a SRC meeting will be convened to decide whether the trial should be restarted using the lower dose of 90mg/m^2^.

Stage A1 will stop for success when either a total of 10 patients have been assigned to a particular treatment schedule or 20 patients have been recruited, whichever occurs first. When 10 patients in Stage A1 have been assigned to a particular treatment schedule, recruitment will be paused until there are no more than three patients without full follow-up (either DLT or 6 weeks after the end of treatment), i.e. until there is full follow-up information on at least seven patients. If the MTD changes, recruitment may start again.

Based on simulations and assuming a patient will be recruited every 8 weeks, the average number of patients required for Stage A1 is 18, which we aim to recruit in 24 months.

## Stage A2

The aim is to find the M6620 (Berzosertib) treatment schedule when combined with palliative combination chemotherapy (Cisplatin and Capecitabine) that is associated with no more than a 30% dose limiting toxicity rate. Four treatment schedules are proposed. Each schedule comprises a specific combination of dose and dosing frequency. There are two possible M6620 (Berzosertib) doses and two dosing frequencies (see section 8.2). Chemotherapy dose remains consistent across all treatment schedules.

The treatment involves six cycles of chemotherapy with three weekly Cisplatin and Capecitabine and M6620 (Berzosertib) at a pre-determined frequency dependent on the treatment schedule allocated to the individual patient. The follow-up of a further 8 weeks provides a total observation window of 26 weeks. DLT assessments will be carried out during the first 4 weeks of treatment. The MTD will be determined during this period using the TiTE-CRM. An initial cohort of three patients will receive the starting schedule (lowest dosing frequency) at the starting dose. The fourth patient will not be recruited until all three patients have been followed for a minimum of 4 weeks from the start of chemotherapy or until the occurrence of a DLT.

From the fourth patient, all eligible patients will be continuously recruited and the TiTE-CRM will be used to assign their treatment schedule. To ensure enough information is accumulated to inform the assignment of the treatment schedule to the subsequent patient, recruitment will be managed by allocating treatment slots (see section 4.4 for further details).

**2.2.1. Stage A2 stopping rules**

Stage A2 will stop for safety if, at any point in the trial, there is sufficient evidence to suggest that schedule 1 is too toxic. More specifically, we will consider schedule 1 to be too toxic if, given all the available data, there is a high probability that the DLT rate is greater than the target toxicity level of 0.3. If the first three patients recruited to Stage A2 have DLTs at treatment schedule 1, then the starting schedule (treatment schedule 1) will be deemed too toxic and the trial will stop.

The trial will stop for success when either six patients have been assigned to the fourth treatment schedule (140 mg/m2 of M6620 (Berzosertib) twice weekly) or 20 patients in total have been recruited, whichever occurs first. When six patients in Stage A2 have been assigned to the fourth treatment schedule, recruitment to Stage A2 will be paused until there is full DLT follow-up information on at least five patients. If the MTD has changed, recruitment to Stage A2 may start again.

Based on simulations and assuming a patient will be recruited every 3 weeks, the average number of patients required for Stage A2 is 16, which we aim to recruit in 12 months.

## Stage B

The aim is to find the M6620 (Berzosertib) treatment schedule when combined with chemoradiotherapy that is associated with no more than 45% dose limiting toxicity rate on the basis that conventional oesophageal chemoradiation causes a grade 3 and 4 toxic event rate of 28% haematological toxicity and 63% non-haematological toxicity, of which 34% is gastrointestinal, as reported in the standard arm of the SCOPE1 study. Comparable toxicity rates were described in the standard arm of the PRODIGE5/ACCORD17 study: grade 3 and 4 neutropenia 29% and grade 3 and 4 dysphagia and oesophagitis 33% ([13](#_ENREF_13)). A maximum of 25 patients will be recruited to Stage B.

There are three proposed M6620 (Berzosertib) treatment schedules (same dose but increasing dosing frequencies) to be explored during Stage B. Before each patient enters Stage B, a TMG will be held to confirm recruitment. If permitted to enrol in the study, the patient will also be assigned a provisional treatment schedule based on the TiTE-CRM’s recommendation. If necessary, a confirmation meeting for the M6620 (Berzosertib) treatment schedule assignment will occur prior to the start of chemoradiotherapy 6 weeks after a patient is recruited. This will maximise the accumulation of information on each patient before deciding on the treatment schedule for the subsequent patient.

The dose of M6620 (Berzosertib) in Stage B will be 140mg/m^2^, allocation will start on schedule 1, which is the middle of the 3 schedules. Recruitment will be continuous; however, escalation will not occur until at least one patient full DLT window of 24 weeks is complete. At this point escalation to schedule 2 will be possible if it is estimated to be safe, and dose decisions thereafter will be made once each new patient is recruited and confirmed (if there is reason to think their allocation may have changed) when they have been treated for 6 weeks (the induction period which is the same for all schedules). De-escalation to schedule -1 is possible at any point in the trial. Although recruitment will be continuous, the TMG retain the option to pause recruitment should they decide more follow-up data is needed before continuing. This may be, for example, to prevent too many patients being treated with a sub-optimal, or too toxic, schedule. No more than 7 patients will be treated on schedule 1 before there is full follow-up data on at least one patient.

We will recommend starting stage B:

- If 10 patients have been recruited to A1 and it has not restarted at the lower dose
- If 10 patients have been assigned to at least schedule 3 in A2 (i.e. are on any of the schedules with a dose of 140mg/m2) or the stopping rule is satisfied (6 treated on schedule 4)

If one of the above starting rules are satisfied then an SRC meeting will be convened to review the data and may recommend starting stage B.

**2.3.1 Stage B stopping rules**

Stage B will stop for safety if, at any point in the trial, there is sufficient evidence to suggest that schedule -1 is too toxic. More specifically, we will consider schedule -1 to be too toxic if, given all the available data, there is a high probability that the DLT rate is greater than the target toxicity level of 0.45. There will be no early stopping rules for success. We expect to recruit a minimum of 15 patients.

## Duration of patient participation

**Stage A1:** Participants will be in the study for 12 weeks from first trial dose or intervention to last protocol visit.

**Stage A2**: Participants will be in the study for maximum of 26 weeks from first trial dose to last protocol visit.

**Stage B:** Participants will be in the study for 24 weeks from first trial dose or intervention to last protocol visit.

## Post-trial care and follow-up

Following the end of study visit, patients will receive standard care. They may receive further chemotherapy if appropriate as per the standard practice of the clinical care team. Patients in Stage A1 & A2 will be followed for progression free survival, overall survival and late toxicity via routine medical oncology follow up clinics. The physician will be asked to provide these data at 6 and 12 months from the start of treatment for Stage A1 and Stage A2.

## Recruitment Schematic

3 patients are recruited and assigned to

- Stage A1: Schedule 1
- Stage A2: Schedule 1

1 patient is recruited and assigned to

- *Stage B: Schedule 1

Recruitment paused for at least 9 weeks from start of treatment of the 3rd patient. If all three patients have toxicities, Stage A1 will be re-started at a lower dose.

Check stopping and safety rules

Stage A2

Stage A1

**Stage A** recruited patients.

Recruitment is paused while enough information is acquired for these.

Recruitment paused for at least 4 weeks from start of treatment of the 3rd patient

Stage B

Once a patient is ready to be recruited, if they are eligible for dose escalation, run the TiTE-CRM model based on

the accumulated critical toxicity data from all participants treated with at least one dose of the IMP within the corresponding trial stage.

Recruitment continuous but no escalation until full 24 week DLT window completed by 1 participant. Max of 7 recruited in the 1^st^ patient’s DLT.

Stopping

rule met:

call SRC meeting

No dose skipping for dose escalation

No restrictions for dose de-escalation

**Stop**

**Trial**

Stopping

rule

**not** met

***Stage B will start once:**

**A1:** 10 patients have been recruited to A1 and it has not restarted at the lower dose or 20 patients have been recruited.

**OR**

**A2**: 10 patients have been assigned to at least treatment schedule 3 or 6 on schedule 4 or the maximum of 20 patients have been recruited.

There are no early success stopping rules for Stage B. The maximum number of participants that can be recruited is 25 patients.

Stage B MTD will be estimated at the end of follow up.

The TMG reviews the output of the TiTE-CRM model and decides on patient’s treatment plan assignment. If TMG cannot reach a decision, SRC will review the decision

Patients are followed up for

Stage A1 – 12 weeks

Stage A2 – 26 weeks

Stage B – 24 weeks

# Objectives and endpoints

| **Primary Objective** | **Endpoints/ Outcome Measures** | **Time point(s) for evaluation of end point** |
| --- | --- | --- |
| - To determine the best tolerated M6620 (Berzosertib) treatment schedule (or phase II recommended dose (RPTD)) administered concomitantly with Radiotherapy only in the palliative treatment of oesophageal cancer | Highest treatment schedule resulting in less than 25% dose limiting toxicity (DLT) rate (see section 9.1.2 for DLT definitions) | - Week 9 |
| **Secondary Objectives** | **Endpoints/ Outcome Measures** |  |
| - To determine the safety and toxicity profile of M6620 (Berzosertib) administered concomitantly with RT only in the palliative treatment of oesophageal cancer | - Any toxicity grade ≥3 graded according to CTCAE v4.03 and length of time for toxicity to resolve | - During radiotherapy Weeks 1-3 - Week 4, 9 and week 12 |
| - To determine if M6620 (Berzosertib) can be delivered in combination with palliative RT | - Proportion of patients completing at least 75%, 90% and 100% of the planned RT dose | - End of radiotherapy   End of Week 3 |
| - Efficacy of the combination | - Objective tumour response (OR) as evaluated by CT scan and quantified by Response Criteria Evaluation (RECIST 1.1) - PFS and OS from D1 - In field radiotherapy control | - 12 weeks - 6 and 12 months |
| **Tertiary Objectives** | **Endpoints/ Outcome Measures** |  |
| - Explore tumour characteristics associated with response | - Genotyping of tumours | - Pre-trial archival biopsy |

## 3.1 Stage A

## Stage A1

## Stage A2

| **Primary Objective** | **Endpoints/ Outcome Measures** | **Time point(s) for evaluation of end point** |
| --- | --- | --- |
| - To determine the best tolerated M6620 (Berzosertib) treatment schedule (or phase II recommended dose (RPTD)) administered concomitantly with chemotherapy (Cisplatin and Capecitabine) only in the palliative treatment of solid cancer | - Highest treatment schedule resulting in less than 30% dose limiting toxicity (DLT) rate (see section 9.1.2 for DLT definitions) | - Week 4 |
| **Secondary Objectives** | **Endpoints/ Outcome Measures** |  |
| - To determine the safety and toxicity profile of M6620 (Berzosertib) administered concomitantly with chemotherapy (Cisplatin and Capecitabine) only in the palliative treatment of solid cancer | - Any toxicity grade ≥3 graded according to CTCAE v4.03 and length of time for toxicity to resolve | - During chemotherapy Week 1-18 - Week 20, 26 |
| - To determine if M6620 (Berzosertib) can be delivered in combination with palliative chemotherapy | - Proportion of patients completing at least 75%, 90% and 100% of the planned dose | - End of chemotherapy Week 18 |
| - Efficacy of the combination | - Objective tumour response (OR) as evaluated by CT scan and quantified by Response Criteria Evaluation (RECIST 1.1) - PFS and OS from D1 | - Week 6, 12, 18, 26 - Week 26 & 12 months |

## 3.2 Stage B

| **Primary Objective** | **Endpoints/ Outcome measures** | **Time point(s) for evaluation of end point** |
| --- | --- | --- |
| - To determine the best tolerated M6620 (Berzosertib) treatment schedule (or phase II recommended dose (RPTD)) administered concomitantly with radiotherapy (dCRT) in combination with cisplatin and capecitabine in the radical treatment of oesophageal cancer | Highest treatment schedule resulting in less than 45% dose limiting toxicity (DLT) rate (see section 9.1.2 for DLT definitions) | - Up to Week 24 |
| **Secondary Objectives** | **Endpoints/ Outcome Measures** |  |
| - To determine the safety and toxicity profile of M6620 (Berzosertib) administered concomitantly with dCRT in combination with cisplatin and capecitabine in the radical treatment of oesophageal cancer | - Any toxicity grade ≥3 graded according to CTCAE v4.03 and length of time for toxicity to resolve | - Up to week 24 |
| - To determine tolerance and ability to deliver M6620 (Berzosertib) in combination with standard dCRT | - Treatment tolerance and deliverability measured by proportion of patients completing at least 80% of the planned chemotherapy dose and at least 20 fractions of RT | - End of induction chemotherapy and dCRT. End of week 11 |
| - Efficacy and safety of the combination | - Objective tumour response (OR) as evaluated by CT scan and quantified by Response Criteria Evaluation (RECIST 1.1) and endoscopic and biopsy findings. - PFS and OS from D1 | - 24 weeks |
| **Tertiary/Exploratory Objectives** | **Endpoints/ Outcome Measures** |  |
| - To explore target effects in tissue | - Change in level of ATR inhibition and apoptosis in M6620 (Berzosertib) treated tissue using IHC. - Genotyping of tumours - Aim to identify markers for oesophageal cancer in the blood | - Biopsies at baseline, week 7 and 24 - Blood samples at baseline, week 7 and week 12 |

# Patient selection

Written informed consent must be obtained before any study specific procedures are performed. The Investigator will determine patient eligibility based on the following criteria.

## Eligibility criteria

***Inclusion criteria:***

A patient will be eligible for inclusion in this study if all of the following criteria apply.

**Stage A1**

1. Histologically confirmed adenocarcinoma or squamous cell carcinoma of the oesophagus (not including cervical oesophagus).
2. Tumour length 15 cm or less.
3. Any stage of disease that is unsuitable for radical CRT or surgery but suitable for palliative RT.
4. Baseline investigations available: staging CT scan (within 42 days before first study dose) and endoscopy.
5. Previous chemotherapy treatment completed 28 days before first study dose.
6. No oesophageal stent in situ.
7. Any gender, age ≥ 16 years.
8. Life expectancy of at least 12 weeks.
9. ECOG performance score of 0-1.
10. Able to comply with protocol fully - absence of any physical, psychological, familial, sociological or geographical condition potentially hampering compliance with the study protocol and follow-up schedule; those conditions should be discussed with the patient before registration in the trial.
11. Able to give written (signed and dated) informed consent according to GCP before registration.
12. Haematological and biochemical indices within the ranges shown below:

| Lab Test | Value required |
| --- | --- |
| Haemoglobin (Hb) | ≥ 8.0 g/dL |
| Platelet count | ≥ 100 x 10^9/L |
| Absolute neutrophil count (ANC) | ≥ 1.5 x 10^9/L |
| Total bilirubin | ≤ 1.5 x upper limit of normal unless the subject has known or suspected Gilbert’s syndrome |
| AST (SGOT)/ALT (SGPT) | ≤ 2.5 x upper limit of normal; ≤5 X ULN if liver metastases |
| Estimated glomerular filtration rate | ≥40mL/min |

**Stage A2**

1. **Any** histologically confirmed advanced solid tumour that is metastatic or unresectable where investigator considers Cisplatin and Capecitabine based regimen as appropriate.
2. Baseline investigations available: staging CT scan (within 35 days before first study dose).
3. Previous chemotherapy treatment completed 28 days before first study dose.
4. Any gender, age ≥ 16 years.
5. Life expectancy of at least 12 weeks.
6. ECOG performance score of 0-1.
7. Able to comply with protocol fully - absence of any physical, psychological, familial, sociological or geographical condition potentially hampering compliance with the study protocol and follow-up schedule; those conditions should be discussed with the patient before registration in the trial.
8. Able to give written (signed and dated) informed consent according to GCP before registration.
9. Haematological and biochemical indices within the ranges shown below:

| Lab Test | Value required |
| --- | --- |
| Haemoglobin (Hb) | ≥ 10.0 g/dL |
| Platelet count | ≥ 100 x 10^9/L |
| Absolute neutrophil count (ANC) | ≥ 1.5 x 10^9/L |
| Total bilirubin | ≤ 1.5 x upper limit of normal unless the subject has known or suspected Gilbert’s syndrome |
| AST (SGOT)/ALT (SGPT) | ≤ 2.5 x upper limit of normal or ≤5 x ULN in presence of liver metastases |
| Ca, Mg, Phosphate | Normal limits |
| Estimated glomerular filtration rate | ≥60mL/min |

**Stage B**

1. Histologically confirmed adenocarcinoma or squamous cell carcinoma of the oesophagus including Siewert type 1 or 2 tumours with ≤2cm gastric mucosal extension (not including cervical oesophagus).
2. Tumour length 7cm or less.
3. Suitable for radical CRT and surgery not an option due to being medically unfit or unsuitable for surgery or patient choice.
4. No oesophageal stent in situ.
5. Endoscopically or radiologically documented measureable disease.
6. Diagnostic PET CT scan*
7. Staging CT scan*

*either CT or PET CT within 42 days of first study dose

1. Adequate respiratory and cardiac function tests for safe delivery of CRT in the opinion of the Principal Investigator, specifically cardiac ejection fraction ≥60% and lung function FEV1 >1 litre or 40% of predicted value or KCO (DLCO/VA) > 40% predicted value.
2. Any gender, age ≥ 16 years.
3. ECOG performance score of 0-1.
4. Able to comply with protocol fully - absence of any physical, psychological, familial, sociological or geographical condition potentially hampering compliance with the study protocol and follow-up schedule; those conditions should be discussed with the patient before registration in the trial.
5. Able to give written (signed and dated) informed consent according to GCP before registration.
6. Haematological and biochemical indices within the ranges shown below:

| Lab Test | Value required |
| --- | --- |
| Haemoglobin (Hb) | ≥ 10.0 g/dL |
| Platelet count | ≥ 100 x 10^9/L |
| Absolute neutrophil count (ANC) | ≥ 1.5 x 10^9/L |
| Total bilirubin | ≤ 1.5 x upper limit of normal unless the subject has known or suspected Gilbert’s syndrome |
| AST (SGOT)/ALT (SGPT) | ≤ 2.5 x upper limit of normal |
| Ca (corrected), Mg, Phosphate | Normal limits |
| Estimated glomerular filtration rate | ≥60mL/min |

***Exclusion criteria:***

A patient will not be eligible for the trial if any of the following apply:

1. Pregnant or breast-feeding women or women of childbearing potential unless highly effective methods of contraception are used. (see Section 5.2)
2. Untreated and multiple brain metastases.
3. Clinically significant cardiovascular event within 6 months before study entry to include:
   1. congestive heart failure requiring therapy
   2. unstable angina pectoris
   3. myocardial infarction
   4. Class II/III/IV cardiac disease (New York Heart Association)
   5. presence of severe valvular heart disease;
   6. presence of a ventricular arrhythmia requiring treatment
4. History of arrhythmia that is symptomatic or requires treatment (CTCAE 3), symptomatic or uncontrolled atrial fibrillation, despite treatment, or asymptomatic sustained ventricular tachycardia. Subjects with atrial fibrillation controlled by medication are permitted.
5. Uncontrolled hypertension (blood pressure ≥160/100 despite optimal therapy).
6. Second or third degree heart block with or without symptoms.
7. QTc >450 msec in adult male and >470 msec in adult females (by Fridericia’s correction) not due to electrolyte abnormality and that does not resolve with correction of electrolytes.
8. History of congenital long QT syndrome.
9. History of torsades de pointes (or any concurrent medication with a known risk of inducing torsades de pointes).
10. Trachea-oesophageal fistula or invasion of the tracheo-bronchial tree.
11. Treatment with any other investigational agent, or treatment in another clinical trial within 28 days prior to treatment start.
12. Strong CYP3A inhibitors and inducers or Haemopoetic growth factors within 14 days before first dose M6620 (Berzosertib).
13. HER2 gastro-oesophageal positive cancer where anti-Her2 therapies may be more appropriate (however patients who have failed anti-HER2 therapy may be eligible for stage A1 and A2).
14. Unable to have or unwilling to change to low molecular weight heparin instead of Warfarin.
15. Other psychological, social or medical condition, physical examination finding or a laboratory abnormality that the Investigator considers would make the patient a poor trial candidate or could interfere with protocol compliance or the interpretation of trial results.
16. Any other active malignancy, with the exception of adequately treated cone-biopsied in situ carcinoma of the cervix uteri and non-melanoma skin lesions.
17. Patients who are known to be serologically positive for active infection with Hepatitis B, Hepatitis C or HIV.

**Additional exclusion criteria Stage A1 and B**

1. Previous radiotherapy to thorax or upper abdomen.

**Additional exclusion criteria Stage A2 and B**

1. History of hand-foot syndrome.
2. History of hearing impairment.
3. Live vaccine received within 30 days prior to treatment start.
4. Complete or Partial DPD deficiency.

**Additional exclusion criteria Stage B**

1. Previous chemotherapy.

## Protocol deviations and waivers to entry criteria

Protocol adherence is a fundamental part of the conduct of a clinical study. Changes to the approved protocol need prior approval unless for urgent safety measures.

Investigators must contact OCTO to obtain guidance and/or clarification as necessary if unsure whether the patient satisfies all the entry criteria and to clarify matters of clinical discretion. OCTO will contact the Chief Investigator or clinical coordinators as necessary. Investigators should not request a protocol waiver to enter a patient who does not satisfy the selection criteria.

## Re-screening if patient does not meet inclusion/exclusion criteria first time round

Patients may be re-screened once, for example if a slot is not available.

## Patient registration

Participants will be recruited from patients usually referred from the upper gastrointestinal MDT.

A screening log must be kept of all patients considered for the study including any that are subsequently excluded; the reason for exclusion must be recorded on this form. A copy of the screening log should be sent to the Trial Office on request, but without patient identifiers. The original must be retained on site.

Before entering a patient onto the study the Principal Investigator or designee will confirm eligibility. If in any doubt the Chief Investigator must be consulted before entering the patient. Details of the query and outcome of the decision should be documented.

Patient recruitment will be managed by the allocation of slots and there will be a minimum of 4 weeks between start of treatment for consecutive patients in stage A1 and a minimum of 3 weeks in stage A2. However, the TMG may decide to release a slot sooner if there is sufficient accumulated experience of the current schedule. If the TMG release a slot sooner, the patient can start treatment before the 3 & 4 week minimum treatment gap. In Stage B, recruitment will be managed by the allocation of slots but a pause between the treatment of consecutive patients is not required.

## Registration procedure

The site should contact OCTO to check the availability of a screening slot and if available reserve the slot prior to giving out a Participant Information Sheet. A screening number should be requested prior to screening the patient and the site should register the participant within 2 weeks of receiving the screening number or relinquish the slot unless an extension is agreed with the trial office.

Site staff will complete the trial registration form and email the form with an anonymised copy of the histopathology report to the Trial Office to confirm the patient’s eligibility. A copy of the histology report is required for verification of eligibility (which will identify the patient by screening number only). The original copy of the registration form should be stored in the site file and a copy in the patient notes.

M6620 (Berzosertib) dose and schedule assignment will be determined by the TMG before the participant is registered on the trial database. The site (including Principal Investigator, Research Nurse and Trial Pharmacist) will be informed of the dose and treatment schedule by email once a patient is registered by the Trial Office.

# Trial assessments and procedures

## Informed consent

Potential participants will be given a current, approved version of the Patient Information Sheet and Consent Form. They will also receive clear verbal information about the study detailing no less than: the nature of the study; the implications and constraints of the protocol; the known side effects and any risks involved in taking part. It will be explained that they will be free to withdraw from the study at any time, for any reason, without prejudice to future care, and with no obligation to give a reason for withdrawal. They will have at least 24 hours to consider the information provided and the opportunity to question the Investigator, their GP or other independent parties before deciding whether to participate.

The Investigator who obtains consent must be suitably qualified and experienced. All delegates must be authorised by the Principal Investigator to obtain consent. The Investigator is responsible for ensuring that the trial consent procedures comply with current applicable GCP Regulatory and ethical requirements. Informed consent discussions and outcomes must be well documented in the medical record. The Investigator must be satisfied that the patient has made an informed decision before taking consent. The patient and the Investigator must personally sign and date the current approved version of the informed consent form in each other’s presence. A copy of the Patient Information Sheet and signed consent form will be given to the participant. The original signed form will be retained in the Investigator Site File (if local policy permits) at the trial site, with a copy held in the medical record. Patient consent will be checked using the consent notification section in the Patient Registration Form.

## Contraception Requirement and Contraceptive/ Pregnancy counselling

## Contraception Requirement

M6620 (Berzosertib) has not been assessed in developmental and reproductive toxicity studies at this stage of development. However, M6620 (Berzosertib) inhibits DNA damage repair and will be administered in conjunction with cytotoxic radiotherapy and chemotherapy, thus the potential for teratogenicity should be considered high. Subjects will be required to take stringent measures to avoid fathering or bearing children while on study drug and for 6 months after discontinuation of M6620 (Berzosertib).

## Female participant of child-bearing potential

Female participants of child-bearing potential are required to use highly effective contraceptive measures (see below) from the start of study treatment until a minimum 6 months after completion of all treatment (chemotherapy, radiotherapy and M6620 (Berzosertib)). Highly effective contraceptive methods considered to have a low user dependency* should preferably be used, in particular when contraception is introduced as a result of participation in the clinical trial. The use of birth control methods does not apply if the female partner has a bilateral oophorectomy, hysterectomy or is postmenopausal. Use of a condom by male partners in addition to use of a highly effective contraceptive measure (double barrier method) is not mandated but it can be recommended.

**Highly effective contraceptive measures:**

- Combined (oestrogen and progestogen containing) hormonal contraception associated with inhibition of ovulation (oral, intravaginal, transdermal)
- Progestogen-only hormonal contraception associated with inhibition of ovulation (oral, injectable, implantable*****)
- Intrauterine device (IUD) in place for at least 90 days prior to start of study drug *****
- Intrauterine hormone-releasing system (IUS) *****
- Bilateral tubal occlusion *****
- Vasectomised partner *****(provided that partner is the sole sexual partner of the WOCBP trial participant and that the vasectomised partner has received medical assessment of the surgical success)
- True abstinence: When this is in line with the preferred and usual lifestyle of the subject. Periodic abstinence (e.g. calendar, ovulation, symptothermal, post-ovulation methods), declaration of abstinence for the duration of exposure to IMP, and withdrawal are not acceptable methods of contraception).

## Male participants

Male participants are required to use a condom during treatment (and must avoid donating sperm) for a minimum 6 months after completion of all treatment (chemotherapy, radiotherapy and M6620 (Berzosertib)). Female partners of male participants, who are of child bearing potential, should also consider contraceptive options.

## Pregnancy counselling

Participants will be counselled to inform the Investigator of any pregnancy (also applies to female partners of male trial subjects) occurring within 6 months of the last dose of the study drug. If a pregnancy is confirmed female participants will be withdrawn immediately from any ongoing treatment. Participants will be asked to provide follow-up information on the outcome of any pregnancy and infants will be followed up for a year after birth for congenital abnormality (see section 15 for pregnancy reporting requirements).

## Pre-dosing evaluations (all stages)

The majority of evaluations will be standard of care for patients. The following assessments must be performed/obtained within the 2 weeks (+7 days allowed) before the patient receives the first study dose (unless otherwise specified below). Informed consent must be obtained prior to performing any study specific evaluations. Confirmation of eligibility and registration on study must be completed as soon as possible after consent to allow time for radiotherapy planning in Stage A1.

- Written informed consent
- Demographic details include age, sex, and self-reported race/ethnicity
- Medical History to include cancer history, prior cancer therapies and procedures, reproductive status, smoking history, and clinically significant disease history and concomitant diseases
- Concomitant medications
- Physical examination to include lungs, abdomen, heart, nodal regions, neurological examination and symptom driven examination. Any abnormality identified at baseline should be recorded.
- Mellow score (Stage B only)
- Height, weight and body surface area (BSA)
- ECOG performance status
- Vital signs: systolic/diastolic blood pressure (BP), pulse rate, temperature
- Screening blood tests:
  - Haematology – Hb, white blood cells (WBC) with differential count (neutrophils and lymphocytes) and platelets
  - Biochemistry – sodium, potassium, urea, creatinine, bilirubin, ALP, AST or ALT, Ca, Mg, Phosphate
  - Coagulation – INR, APTT or PTT.
- Pregnancy test (in females of child bearing potential only): serum or urine Human Chorionic Gonadotropin (HCG) test to rule out pregnancy at study entry; results must be obtained and reviewed at least 1 week before first dose of IMP. During treatment applicable patients must have pregnancy testing every 4 weeks. Pregnancy test not required for post-menopausal or surgically sterile females.
- Electrocardiogram (ECG)
- Pathology report confirming histological diagnosis (archival diagnostic sample will be requested for analysis see section 7.3.1).

**In addition for Stage A1 and A2:**

- Staging CT scan within 42 days for Stage A1 and 35 days for Stage A2 of first study dose

**In addition for Stage B:**

- Staging CT or PET scan within 42 days before first study dose
- Echo cardiogram or MUGA (Multi Gated Acquisition Scan) within 3 months prior to start of treatment
- Lung function tests within 3 months prior to start of treatment

**In addition for Stage A2 and B:**

- (If required) Audiogram monitoring for patients with significant hearing impairment
- DPD deficiency testing (any time before trial enrolment). This is not required for Stage A2 participants with previous use of Capecitabine.

## Stage A Evaluations

## Stage A1 evaluations during the study

**Post registration and prior to start of Radiotherapy**

- M6620 (Berzosertib) schedule assignment
- Radiotherapy planning (or pre-registration during screening)

**Evaluations Week 1:**

The following assessments should be done Day 1 and Day 4 (+/-24 hours but must be pre-M6620 (Berzosertib)) unless stated otherwise:

- Venous blood sample within 24 hours prior to M6620 (Berzosertib) including:
- Haematology – Haemoglobin, white blood cells (WBC) with differential count (neutrophils and lymphocytes) and platelets
- Biochemistry – sodium, potassium, urea, creatinine, bilirubin, ALP, AST or ALT
- Coagulation – INR, APTT or PTT.
- Adverse Event (AE) Assessment
- Assessment of concomitant medications
- Physical examination
- Weight (Day 1 only)
- ECOG performance status
- Assessment of DLT (Day 4 only)
- ECG (Day 1 prior to treatment)
- On days of M6620 (Berzosertib) monitor patient for reactions for 20 minutes after administration

**Evaluation during radiotherapy and concomitant M6620 (Berzosertib) (Weeks 2 - 3)**

The following assessments should be done the day before each administration of M6620 (Berzosertib) (+/-24 hours but must be pre-M6620 (Berzosertib)) unless stated otherwise:

- Venous blood sample within 24 hours prior to administration of M6620 (Berzosertib) including:
- Haematology – Haemoglobin, white blood cells (WBC) with differential count (neutrophils and lymphocytes) and platelets
- Biochemistry – sodium, potassium, urea, creatinine, bilirubin, ALP, AST or ALT
- Assessment of AE
- Assessment of concomitant medications
- Physical examination
- Weight (Day 8 & 15 only)
- ECOG performance status
- Assessment of DLT
- On days of M6620 (Berzosertib) monitor patient for reactions for 20 minutes after administration

**Evaluations on Week 4, 9 & 12**

- Assessment of AE
- Assessment of concomitant medications
- Physical examination
- Weight
- ECOG performance status
- Assessment of DLT (not week 12)
- Coagulation – INR, APTT or PTT (week 4 only)
- Haematology – Haemoglobin, white blood cells (WBC) with differential count (neutrophils and lymphocytes) and platelets (week 4 only)
- Biochemistry – sodium, potassium, urea, creatinine, bilirubin, ALP, AST or ALT (week 4 only)ECG (weeks 4 & 12 only)
- CT chest, abdomen, pelvis (week 12 only). If disease progression is already identified by CT scan during the follow up period, a repeat CT scan is not necessary at week 12.

## Stage A1 evaluations on early withdrawal

Where possible, patients will be followed up as per the follow up visits which should be brought forward to 1, 6 and 9 weeks post end of treatment; including assessments of AEs and DLTs see section 6.

## Stage A1 off-Study and Follow-up Evaluations

Where possible, patients will be followed up as per standard of care for 9 weeks post radiotherapy. Patients should be counselled on the continued use of contraception for 6 months following the end of treatment **if appropriate** and encouraged to report any pregnancies to the study team. The clinician in charge will be asked to provide the following information at 6 and 12 months.

- date and cause of death, if applicable
- details of any clinically significant events

Date and site of progression.

- Further radiotherapy, or other intervention required.

## Stage A2 evaluations during the study

**Post registration**

- M6620 (Berzosertib) schedule assignment
- Audiogram monitoring (if applicable)

**Weekly Evaluations during treatment weeks 1 - 18**

- Assessment of concomitant medications
- Weight
- ECOG performance status
- Physical examination
- Assessment of AE
- Assessment of DLT (Weeks 1-4 only)
- ECG (once per cycle, week 1 pre-treatment)
- Coagulation – INR, APTT or PTT (week one only, pre-cisplatin)

**Additional evaluations during treatment weeks 1 - 18**

- Venous blood sample within 72 hours prior to Cisplatin including:
- Haematology – Hb, white blood cells (WBC) with differential count (neutrophils and lymphocytes) and platelets
- Biochemistry – sodium, potassium, urea, creatinine, bilirubin, ALP and AST or ALT, Ca, Mg, Phosphate
- Venous blood samples within 24 hours prior to M6620 (Berzosertib) administration including:
- Haematology – Hb, white blood cells (WBC) with differential count (neutrophils and lymphocytes) and platelets
- Biochemistry – sodium, potassium, urea, creatinine, bilirubin, ALP and AST or ALT, Ca, Mg, Phosphate

**Note: only one set of blood tests needs to be done if the timing satisfies both Cisplatin and M6620 (Berzosertib) requirements above.**

- On days of M6620 (Berzosertib) monitor patient for reactions for 20 minutes after administration

**Additional evaluation Week 4**

- Audiogram monitoring (if applicable)

**Additional evaluation Week 6, 12, 18**

- CT chest abdomen and pelvis to assess response

**Evaluations at 2 & 8 week follow up**

- Venous blood samples:
  - Haematology – Hb, white blood cells (WBC) with differential count (neutrophils and lymphocytes) and platelets
  - Biochemistry – sodium, potassium, urea, creatinine, bilirubin, ALP and AST or ALT, Ca, Mg, Phosphate
- Assessment of concomitant medications
- Weight, ECOG performance status
- Physical examination
- Coagulation – INR, APTT or PTT (week 2 only)
- Assessment of AE
- CT chest abdomen and pelvis to assess response (week 26 only)

## Stage A2 evaluations on early withdrawal

Where possible, patients will be followed up as per the follow up visits at 2 and 8 weeks post end of treatment, including assessments of AEs and DLTs (see section 6).

## Stage A2 off-Study and Follow-up Evaluations

The final study visit will occur 8 weeks after completion of treatment. Patients should be counselled on the continued use of contraception for 6 months following the end of treatment **if appropriate** and encouraged to report any pregnancies to the study team. The clinician in charge will be asked to provide the following reports at 6 & 12 months.

- date and cause of death, if applicable
- date and site of progression
- details of any clinically significant events
- further chemotherapy or other intervention required

## Stage B evaluations

**Post registration and prior to start of Induction chemotherapy**

- Research blood sample
- Audiogram monitoring (if applicable)

## Stage B evaluations during the study

**Evaluations during treatment weeks 1 & 4 (induction chemotherapy + M6620 (Berzosertib)) and weeks 7, 8, 9, 10 & 11 (chemoradiotherapy + M6620 (Berzosertib))**

- The following assessments should be done within 24 hours prior to M6620 (Berzosertib) administration: Venous blood samples within 24 hours prior to M6620 (Berzosertib) administration including:
  - Haematology – Hb, white blood cells (WBC) with differential count (neutrophils and lymphocytes) and platelets
  - Biochemistry – sodium, potassium, urea, creatinine, bilirubin, ALP and AST or ALT, Ca, Mg, Phosphate
- Assessment of concomitant medications
- Weight
- ECOG performance status
- Vital signs: systolic/diastolic blood pressure (BP), pulse rate, temperature
- Physical examination
- ECG (weeks 1, 4, 7 and 10 pre-cisplatin treatment)
- Coagulation (INR, APTT or PTT, week 1 only pre-cisplatin)
- Mellow score
- Assessment of AE
- Assessment of DLT (not week 1)

The following assessments should be done on days of M6620 (Berzosertib) administration during induction chemotherapy. If problems were identified, this should be continued during chemoradiotherapy:

- Monitor patient for reactions for 20 minutes after administration of M6620 (Berzosertib)

**Additional assessments weeks 1, 4, 7 & 10**

- Venous blood sample within 72 hours prior to Cisplatin including:
- Haematology – Hb, white blood cells (WBC) with differential count (neutrophils and lymphocytes) and platelets
- Biochemistry – sodium, potassium, urea, creatinine, bilirubin, ALP and AST or ALT, Ca, Mg, Phosphate

**Note: only one set of blood tests needs to be done if the timing satisfies both Cisplatin and M6620 (Berzosertib) requirements above.**

**Additional evaluation week 4**

- Audiogram monitoring (if applicable)

**Additional assessments week 7 (see section 7.3 for details)**

- Research endoscopy and biopsy
- Research blood sample

**Radiotherapy planning should take place in weeks 2 to 4 and M6620 (Berzosertib) schedule assignment will take place in week 6**

**Evaluations during induction chemotherapy weeks 2 & 5**

Evaluations to be done on day 1 or day 2:

- Assessment of concomitant medications
- ECOG performance status
- Vital signs: systolic/diastolic blood pressure (BP), pulse rate, temperature
- Physical examination
- Mellow score
- Assessment of AE
- Assessment of DLT

**Evaluations at weeks 12 & 18**

- Venous blood samples:
  - Haematology – Hb, white blood cells (WBC) with differential count (neutrophils and lymphocytes) and platelets
  - Biochemistry – sodium, potassium, urea, creatinine, bilirubin, ALP and AST or ALT, Ca, Mg, Phosphate
- Assessment of concomitant medications
- ECOG performance status
- Vital signs: systolic/diastolic blood pressure (BP), pulse rate, temperature
- Coagulation (INR, APTT or PTT, week 12 only)
- Physical examination
- Mellow score
- Assessment of AE
- Assessment of DLT
- Research blood sample (week 12 only)

**Evaluations at week 24**

- Assessment of concomitant medications
- ECOG performance status
- Vital signs: systolic/diastolic blood pressure (BP), pulse rate, temperature
- Physical examinations
- ECG
- Mellow score
- Assessment of AE
- Assessment of DLT
- Endoscopy and research biopsy
- CT chest abdomen and pelvis to assess response

## Stage B evaluations on early withdrawal

Where possible, patients will be followed up as per the follow up visits which should be brought forward to 1, 7 and 13 weeks post end of treatment, including assessments of AEs and DLTs see section 6.

## Stage B off-Study and Follow-up Evaluations

The final study visit will occur 13 weeks after completion of treatment. Patients should be counselled on the continued use of contraception for 6 months following the end of treatment **if appropriate** and encouraged to report any pregnancies to the study team. Sites will report this if notified up until 6m or 13w after last patient treatment whichever is sooner.

# Early patient withdrawal

**The Trial Office should be informed of any early patient withdrawal within 24 hours of the site becoming aware using the Early Withdrawal Form and scan and email as a PDF attachment to**

[**octo-safety@oncology.ox.ac.uk**](mailto:octo-CHARIOT@oncology.ox.ac.uk)**. If the reason for early withdrawal is an SAE then an SAE Form will also be required.**

## Treatment Withdrawal

During the course of the trial, a patient may withdraw early from treatment. This may happen for a number of reasons, including:

- Unacceptable toxicity
- AE/SAEs requiring discontinuation
- Loss to follow-up
- Significant protocol deviation or inability to comply with trial procedures
- Clinical decision
- Patient decision

When the patient stops treatment early, the ‘End of Treatment’ Form needs to be completed, and any other relevant CRFs (example SAE Form). Any evaluations carried out on early withdrawal will be captured as per sections 5.4.2, 5.4.5 and 5.5.2. The reason for withdrawing from treatment early should be clearly documented in the medical records.

The end of treatment means the patient will then enter the routine follow up stage of the trial. If M6620 (Berzosertib) treatment is stopped, the patient will continue with standard treatment and will be followed up as part of the trial.

## Consent Withdrawal

Consent withdrawal means that a patient has expressed a wish to withdraw from the study altogether. Under these circumstances, the site needs to document all relevant discussions in the patient notes and notify the Trial Office, which will allow the office to mark all future CRFs as not applicable. The site should inform the Trial Office whether any samples already collected for the study should be destroyed.

Under these conditions, investigators are still responsible to follow up any SAEs till resolution.

## 6.3 Patient evaluability and replacement

Patients will not be replaced since TiTE-CRM uses accumulated data and all patients will be evaluable for dose escalation decisions. However, the TMG may decide to replace patients if drop-out occurs early in the treatment schedule for reasons other than a DLT.

In Stage B, all patients who receive treatment within the study will be evaluable for response. All participants who receive one dose of M6620 (Berzosertib) will be evaluable for the safety analysis.

Evaluable for Objective Response. Only those patients who have measurable disease present at baseline, have received at least one cycle of therapy, and have had their disease re-evaluated will be considered evaluable for response. These patients will have their response classified according to the definitions stated in Appendix B. (Patients who exhibit objective disease progression prior to the end of cycle 1 will be considered evaluable.)

Evaluable Non-Target Disease Response. Patients who have lesions present at baseline that are evaluable but do not meet the definitions of measurable disease, have received at least one cycle of therapy, and have had their disease re-evaluated will be considered evaluable for non-target disease. The response assessment is based on the presence, absence, or unequivocal progression of the lesions.

# Samples for laboratory analysis

## Samples to be analysed in local Trust’s laboratories

### Diagnostic Laboratories

Samples for haematology and biochemistry analysis will be labelled with standard patient identifiers and sent to the local hospital diagnostic laboratory. Results will be processed in the standard way and entered into the routine hospital reporting system. Samples will be stored, held, reported and subsequently destroyed in accordance with standard local laboratory practice.

### Pathology

The routine diagnostic pathology samples and additional research samples taken at endoscopy will also be labelled, processed and reported according to the standards proposed by the Royal College of Pathologists. An anonymised copy of the diagnostic histopathology/cytology report should be sent to the Trial Office at registration.

## Blood and Tissue samples for translational research

Blood and biopsy samples will be collected in Stage B for translational research concerning the mechanism of action of M6620 (Berzosertib).

## Biopsy samples

Tissue samples will be collected at least 24 hours after the first dose of M6620 (Berzosertib) treatment. The samples will be analysed for a signal of ATR inhibition by M6620 (Berzosertib) following induction of the DDR (ATR activity) by Cisplatin or RT treatment.

Tissue should be biopsied from 3 areas of the oesophagus: normal tissue outside the radiotherapy field, normal tissue within the radiotherapy field and tumour tissue within the radiotherapy field. This will allow a comparison of the effect of M6620 (Berzosertib) on healthy tissue, irradiated tissue and tumour tissue.

All participants will have a diagnostic biopsy prior to screening. Baseline FFPE tissue samples will be analysed for common genetic mutations using a cancer panel.

**Timepoints for endoscopy and biopsy:**

- week 7 day 3, and if M6620 (Berzosertib) given carried out at least 24 hours after the administration of M6620 (Berzosertib) (on treatment biopsy should be taken from fraction 3 to fraction 5 (but up to fraction 7 is permitted if required)
- week 24

Samples will be sent to the central lab for IHC analysis (see Sample Handling Manual for further details). Remaining samples will be returned to Oxford for further IHC markers and storage in Oxford research biobank (details will be in the sampling handling manual).

## Research blood samples

Blood samples will be collected to look for biomarkers for ATR inhibition and to identify other DNA, RNA or protein markers present in oesophageal cancer.

**Timepoints for research blood samples:**

- prior to starting treatment
- week 7 from fraction 3 to fraction 5 (but up to fraction 7 is permitted if required)
- week 12

Samples will be sent to Oxford University Labs for analysis (details will be in the sampling handling manual).

## Labelling and confidentiality of samples sent

All samples sent to analytical Laboratories will be labelled with the trial code, trial patient number, schedule and date/time taken. Should a laboratory receive any samples carrying unique patient identifiers the recipient must immediately obliterate this information and re-label.

## Clinical reporting of exploratory research assay results

The results of the CHARIOT trial research assays are exploratory and are not intended to influence the individual patient’s medical care. Findings will not be reported routinely to the responsible clinician except in the unlikely event that the result might be beneficial to the patient’s clinical management.

## Trial sample retention at end of study

The Chief Investigator has overall responsibility for custodianship of the trial samples. Laboratories are instructed to retain any surplus samples pending instruction from the Chief Investigator on use, storage or destruction. It is possible that new or alternative assays may be of future scientific interest. At the end of the research study any surplus samples may be retained for use in other projects that have received ethical approval. Hence, any surplus study samples may be transferred to a licensed tissue bank where they will be managed in accordance with applicable host institution policies and the Human Tissue Act (HTA) requirements.

## Withdrawal of consent for sample collection and/or retention

A patient may withdraw consent to provide samples for research at any time without giving a reason. The Investigator must ensure that their wishes are recorded in the medical record and will inform the Trial Office accordingly. The Investigator should discuss with patients the valuable use of samples that have already been provided and under circumstances where these samples have already been processed and anonymised, it would not be possible to destroy such samples.

# Investigational medicinal products (IMP)

The trial is investigating the unlicensed drug M6620 (Berzosertib) in combination with the radiotherapy (stage A1); M6620 (Berzosertib) in combination with chemotherapy agents Cisplatin and Capecitabine (stage A2) and M6620 (Berzosertib) with chemoradiotherapy (stage B). For the purposes of the trial, M6620 (Berzosertib), Cisplatin and Capecitabine are all considered IMPs.

## Stage A1 Treatment

Two M6620 (Berzosertib) dose levels and 3 dosing frequencies (treatment schedules) are proposed. Both the dose and frequency of M6620 (Berzosertib) will vary but the administered radiation dose and fractionation schedule will remain unchanged across treatment plans. The treatment schedule will last for 3 weeks and radiotherapy must start on a Monday.

Antiemetics should be prescribed as supporting medication to be available from Day 1 of Radiotherapy, ( i.e. Domperidone 20mg tds prn PO 5/7 or Metoclopramide 20mg tds prn PO 5/7, or the preferred standard of care at the institution)

## M6620 (Berzosertib) treatment schedules - Stage A1

The starting dose of M6620 (Berzosertib) will be 140mg/m^2^ IV once weekly (schedule 1). If schedule 1 is too toxic, the trial will be re-started at 90mg/m^2^ (schedule -3). For all schedules see table 8.1. The treatment schedule of M6620 (Berzosertib) will be escalated or de-escalated using the TiTE-CRM model (see section 2 for further details).

**Table 8.1**

| **Dose Escalation schedule** | |
| --- | --- |
| **Treatment schedule** | Dose** of *M6620 (Berzosertib) and days of the schedule it will be delivered* |
| -3 | 90 *mg/m^2^* day 2, 9, 16 |
| -2 | 90 *mg/m^2^* day 2, 5, 9, 12, 16 |
| -1 | 90 *mg/m^2^* day 2, 5, 9, 12, 16, 19 |
| 1* | 140 *mg/m^2^* day 2, 9, 16 |
| 2 | 140 *mg/m^2^* day 2, 5, 9, 12, 16 |
| 3 | 140 *mg/m^2^* day 2, 5, 9, 12, 16, 19 |
| 4 | 240 *mg/m^2^* day 2, 9, 16 |
| 5 | 240 *mg/m^2^* day 2, 5, 9, 12, 16 |
| 6 | 240 *mg/m^2^* day 2, 5, 9, 12, 16, 19 |

**Starting dose and schedule. 90mg/m^2^ dose will only be explored if trial is re-started*

***Doses are stated as exact dose in units. No intermediate dose levels or further splitting of the dose allowed*

## Radiotherapy dose and duration –Stage A1

The total dose of radiation will be 35Gy in 15 fractions treated once daily, 5 days a week Monday to Friday and prescribed and recorded per ICRU 62.

## Stage A1: Dose Escalation Schema

**Schedule -3 – 90 mg/m^2^, 270 mg/m^2^ per plan**

|  | **Week 1** | | | | | | | **Week 2** | | | | | | | **Week 3** | | | | | | |
| --- | --- | --- | --- | --- | --- | --- | --- | --- | --- | --- | --- | --- | --- | --- | --- | --- | --- | --- | --- | --- | --- |
|  | M | T | W | T | F | S | S | M | T | W | T | F | S | S | M | T | W | T | F | S | S |
| **Radiotherapy** | **X** | **X** | **X** | **X** | **X** |  |  | **X** | **X** | **X** | **X** | **X** |  |  | **X** | **X** | **X** | **X** | **X** |  |  |
| **ATR Inhibitor** |  | **M** |  |  |  |  |  |  | **M** |  |  |  |  |  |  | **M** |  |  |  |  |  |

**Schedule -2 – 90 mg/m^2^, 450 mg/m^2^ per plan**

|  | **Week 1** | | | | | | | **Week 2** | | | | | | | **Week 3** | | | | | | |
| --- | --- | --- | --- | --- | --- | --- | --- | --- | --- | --- | --- | --- | --- | --- | --- | --- | --- | --- | --- | --- | --- |
|  | M | T | W | T | F | S | S | M | T | W | T | F | S | S | M | T | W | T | F | S | S |
| **Radiotherapy** | **X** | **X** | **X** | **X** | **X** |  |  | **X** | **X** | **X** | **X** | **X** |  |  | **X** | **X** | **X** | **X** | **X** |  |  |
| **ATR Inhibitor** |  | **M** |  |  | **M** |  |  |  | **M** |  |  | **M** |  |  |  | **M** |  |  |  |  |  |

**Schedule -1 – 90 mg/m^2^, 540 mg/m^2^ per plan**

|  | **Week 1** | | | | | | | **Week 2** | | | | | | | **Week 3** | | | | | | |
| --- | --- | --- | --- | --- | --- | --- | --- | --- | --- | --- | --- | --- | --- | --- | --- | --- | --- | --- | --- | --- | --- |
|  | M | T | W | T | F | S | S | M | T | W | T | F | S | S | M | T | W | T | F | S | S |
| **Radiotherapy** | **X** | **X** | **X** | **X** | **X** |  |  | **X** | **X** | **X** | **X** | **X** |  |  | **X** | **X** | **X** | **X** | **X** |  |  |
| **ATR Inhibitor** |  | **M** |  |  | **M** |  |  |  | **M** |  |  | **M** |  |  |  | **M** |  |  | **M** |  |  |

**Schedule 1 – 140 mg/m^2^ per dose, 420 mg/m^2^ per plan (starting schedule)**

|  | **Week 1** | | | | | | | **Week 2** | | | | | | | **Week 3** | | | | | | |
| --- | --- | --- | --- | --- | --- | --- | --- | --- | --- | --- | --- | --- | --- | --- | --- | --- | --- | --- | --- | --- | --- |
|  | M | T | W | T | F | S | S | M | T | W | T | F | S | S | M | T | W | T | F | S | S |
| **Radiotherapy** | **X** | **X** | **X** | **X** | **X** |  |  | **X** | **X** | **X** | **X** | **X** |  |  | **X** | **X** | **X** | **X** | **X** |  |  |
| **ATR Inhibitor** |  | **M** |  |  |  |  |  |  | **M** |  |  |  |  |  |  | **M** |  |  |  |  |  |

**Schedule 2 – 140 mg/m^2^, 700 mg/m^2^ per plan**

|  | **Week 1** | | | | | | | **Week 2** | | | | | | | **Week 3** | | | | | | |
| --- | --- | --- | --- | --- | --- | --- | --- | --- | --- | --- | --- | --- | --- | --- | --- | --- | --- | --- | --- | --- | --- |
|  | M | T | W | T | F | S | S | M | T | W | T | F | S | S | M | T | W | T | F | S | S |
| **Radiotherapy** | **X** | **X** | **X** | **X** | **X** |  |  | **X** | **X** | **X** | **X** | **X** |  |  | **X** | **X** | **X** | **X** | **X** |  |  |
| **ATR Inhibitor** |  | **M** |  |  | **M** |  |  |  | **M** |  |  | **M** |  |  |  | **M** |  |  |  |  |  |

**Schedule 3 – 140 mg/m^2^, 840 mg/m^2^ per plan**

|  | **Week 1** | | | | | | | **Week 2** | | | | | | | **Week 3** | | | | | | |
| --- | --- | --- | --- | --- | --- | --- | --- | --- | --- | --- | --- | --- | --- | --- | --- | --- | --- | --- | --- | --- | --- |
|  | M | T | W | T | F | S | S | M | T | W | T | F | S | S | M | T | W | T | F | S | S |
| **Radiotherapy** | **X** | **X** | **X** | **X** | **X** |  |  | **X** | **X** | **X** | **X** | **X** |  |  | **X** | **X** | **X** | **X** | **X** |  |  |
| **ATR Inhibitor** |  | **M** |  |  | **M** |  |  |  | **M** |  |  | **M** |  |  |  | **M** |  |  | **M** |  |  |

**Schedule 4 – 240 mg/m^2^, 720 mg/m^2^ per plan**

|  | **Week 1** | | | | | | | **Week 2** | | | | | | | **Week 3** | | | | | | |
| --- | --- | --- | --- | --- | --- | --- | --- | --- | --- | --- | --- | --- | --- | --- | --- | --- | --- | --- | --- | --- | --- |
|  | M | T | W | T | F | S | S | M | T | W | T | F | S | S | M | T | W | T | F | S | S |
| **Radiotherapy** | **X** | **X** | **X** | **X** | **X** |  |  | **X** | **X** | **X** | **X** | **X** |  |  | **X** | **X** | **X** | **X** | **X** |  |  |
| **ATR Inhibitor** |  | **M** |  |  |  |  |  |  | **M** |  |  |  |  |  |  | **M** |  |  |  |  |  |

**Schedule 5 – 240 mg/m^2^, 1200 mg/m^2^ per plan**

|  | **Week 1** | | | | | | | **Week 2** | | | | | | | **Week 3** | | | | | | |
| --- | --- | --- | --- | --- | --- | --- | --- | --- | --- | --- | --- | --- | --- | --- | --- | --- | --- | --- | --- | --- | --- |
|  | M | T | W | T | F | S | S | M | T | W | T | F | S | S | M | T | W | T | F | S | S |
| **Radiotherapy** | **X** | **X** | **X** | **X** | **X** |  |  | **X** | **X** | **X** | **X** | **X** |  |  | **X** | **X** | **X** | **X** | **X** |  |  |
| **ATR Inhibitor** |  | **M** |  |  | **M** |  |  |  | **M** |  |  | **M** |  |  |  | **M** |  |  |  |  |  |

**Schedule 6 – 240 mg/m^2^, 1440 mg/m^2^ per plan**

|  | **Week 1** | | | | | | | **Week 2** | | | | | | | **Week 3** | | | | | | |
| --- | --- | --- | --- | --- | --- | --- | --- | --- | --- | --- | --- | --- | --- | --- | --- | --- | --- | --- | --- | --- | --- |
|  | M | T | W | T | F | S | S | M | T | W | T | F | S | S | M | T | W | T | F | S | S |
| **Radiotherapy** | **X** | **X** | **X** | **X** | **X** |  |  | **X** | **X** | **X** | **X** | **X** |  |  | **X** | **X** | **X** | **X** | **X** |  |  |
| **ATR Inhibitor** |  | **M** |  |  | **M** |  |  |  | **M** |  |  | **M** |  |  |  | **M** |  |  | **M** |  |  |

## Stage A2 Treatment

Two dose levels and 2 dosing frequencies (treatment schedules) are proposed. Both the dose and frequency of M6620 (Berzosertib) will vary but the Cisplatin and Capecitabine dose and schedule will remain unchanged across treatment plans. The treatment schedule will last for 6 cycles (18 weeks).

## M6620 (Berzosertib) treatment schedule – Stage A2

The starting dose of M6620 (Berzosertib) will be 90mg/m^2^ IV once weekly (schedule 1). For all schedules see table 8.2. The treatment schedule of M6620 (Berzosertib) will be escalated or de-escalated using the TiTE-CRM model (see section 2 for further details).

**Table 8.2**

| **Dose Escalation schedule** | |
| --- | --- |
| **Treatment schedule** | Dose of *M6620 (Berzosertib) and days of the schedule it will be delivered* |
| 1 | 90 *mg/m^2^* once a week for 18 weeks (Tuesdays) |
| 2 | 90 *mg/m^2^* twice a week for 18 weeks (Tuesdays and Fridays) |
| 3 | 140 *mg/m^2^* once a week for 18 weeks (Tuesdays) |
| 4 | 140 *mg/m^2^* twice a week for 18 weeks (Tuesdays and Fridays) |

**Doses are stated as exact dose in units. No intermediate dose levels or further splitting of the dose allowed*

## Chemotherapy dose and duration - Stage A2

**Chemotherapy (Weeks 1 – 18)**

Cisplatin 60mg/m^2^ IV Day 1 of 21-day cycle for 6 cycles

Capecitabine 625mg/m^2^ po bd Days 1-21 of 21-day cycle for 6 cycles

## Stage A2 Dose escalation schema

| **Dosing schedule 1,3** |  | **Week 1** | | | | | | | **Week 2** | | | | | | | **Week 3** | | | | | | |
| --- | --- | --- | --- | --- | --- | --- | --- | --- | --- | --- | --- | --- | --- | --- | --- | --- | --- | --- | --- | --- | --- | --- |
|  |  | M | T | W | T | F | S | S | M | T | W | T | F | S | S | M | T | W | T | F | S | S |
|  | **Capecitabine** |  |  |  |  |  |  |  |  |  |  |  |  |  |  |  |  |  |  |  |  |  |
|  | **Cisplatin** | **C** |  |  |  |  |  |  |  |  |  |  |  |  |  |  |  |  |  |  |  |  |
|  | **ATR Inhibitor** |  | **M** |  |  |  |  |  |  | **M** |  |  |  |  |  |  | **M** |  |  |  |  |  |

Repeat this 3 weekly schedule for 6 cycles

| **Dosing schedule 2, 4** |  | **Week 1** | | | | | | | **Week 2** | | | | | | | **Week 3** | | | | | | |
| --- | --- | --- | --- | --- | --- | --- | --- | --- | --- | --- | --- | --- | --- | --- | --- | --- | --- | --- | --- | --- | --- | --- |
|  |  | M | T | W | T | F | S | S | M | T | W | T | F | S | S | M | T | W | T | F | S | S |
|  | **Capecitabine** |  |  |  |  |  |  |  |  |  |  |  |  |  |  |  |  |  |  |  |  |  |
|  | **Cisplatin** | **C** |  |  |  |  |  |  |  |  |  |  |  |  |  |  |  |  |  |  |  |  |
|  | **ATR Inhibitor** |  | **M** |  |  | **M** |  |  |  | **M** |  |  | **M** |  |  |  | **M** |  |  | **M** |  |  |

Repeat this 3 weekly schedule for 6 cycles

## Stage B Treatment

The dose administered in stage B will be 140mg/m^2^ (see section 2.3) and will remain constant whilst three dosing schedules are explored (see table 8.3). The chemotherapy and radiation doses and fractionation schedules will remain unchanged across dosing schedules.

The 11 weeks of treatment consists of 6 weeks of induction chemotherapy (Capecitabine and Cisplatin) with M6620 (Berzosertib) followed by 5 weeks of concomitant chemoradiotherapy (Capecitabine, Cisplatin and radiotherapy) with M6620 (Berzosertib). All patients will receive M6620 (Berzosertib) with induction chemotherapy on Cycle 1 Day 2 and Cycle 2 Day 2. In the last week of chemotherapy patients will be assigned to a M6620 (Berzosertib) treatment schedule to be administered during chemoradiotherapy (see table 8.3 below). Radiotherapy must start on a Monday.

## M6620 (Berzosertib) Treatment dose and schedule – Stage B

The dose of M6620 (Berzosertib) in Stage B will be 140mg/m^2^, allocation will start on schedule 1.

**Table 8.3**

| **Dose Escalation Schedule** | | |
| --- | --- | --- |
| **Treatment Schedule** | **M6620 (Berzosertib) administration during induction chemotherapy** | **M6620 (Berzosertib) administration during Chemoradiotherapy** |
| -1 | Cycle 1 day 2,  Cycle 2 day 2 | Days 2, 9, 16, 23, 30 |
| 1* | As above | Days 2, 5, 9, 16, 23, 26, 30 |
| 2 | As above | Days 2, 5, 9, 12, 16, 19, 23, 26, 30, 33 |

**Starting schedule. Reduced frequency schedule (-1) will only be explored if schedule 1 is too toxic.*

## Chemoradiotherapy dose and duration - Stage B

**Induction Chemotherapy (Weeks 1 – 6)**

Cisplatin 60mg/m^2^ IV Day 1 of 21-day cycle for 2 cycles (Days 1 and day 22 of chemotherapy)

Capecitabine 625mg/m^2^ po bd Days 1-21 of 21-day cycle for 2 cycles

**Concurrent chemoradiotherapy (Weeks 7 – 11)**

Cisplatin 60mg/m^2^ IV Day 1 of 21-day cycle for 2 cycles (Days 1 and day 22 of chemo**Radio**therapy)

Capecitabine 625mg/m^2^ po bd on days receiving radiotherapy (From day 1 to day 33 of chemoradiotherapy **excluding** days not receiving radiotherapy) (Total 25 days of treatment)

## Radiotherapy dose and duration - Stage B

The total dose of radiation will be 50Gy in 25 fractions treating once daily, 5 days per week Monday to Friday and prescribed and recorded as per ICRU 62.

## Stage B Dose frequency escalation schema

**Induction Chemotherapy - All patients**

|  | **Week 1** | | | | | | | **Week 2** | | | | | | | **Week 3** | | | | | | |
| --- | --- | --- | --- | --- | --- | --- | --- | --- | --- | --- | --- | --- | --- | --- | --- | --- | --- | --- | --- | --- | --- |
|  | M | T | W | T | F | S | S | M | T | W | T | F | S | S | M | T | W | T | F | S | S |
| **Capecitabine** |  |  |  |  |  |  |  |  |  |  |  |  |  |  |  |  |  |  |  |  |  |
| **Cisplatin** | **C** |  |  |  |  |  |  |  |  |  |  |  |  |  |  |  |  |  |  |  |  |
| **ATR Inhibitor** |  | **M** |  |  |  |  |  |  |  |  |  |  |  |  |  |  |  |  |  |  |  |

|  | **Week 4** | | | | | | | **Week 5** | | | | | | | **Week 6** | | | | | | |
| --- | --- | --- | --- | --- | --- | --- | --- | --- | --- | --- | --- | --- | --- | --- | --- | --- | --- | --- | --- | --- | --- |
|  | M | T | W | T | F | S | S | M | T | W | T | F | S | S | M | T | W | T | F | S | S |
| **Capecitabine** |  |  |  |  |  |  |  |  |  |  |  |  |  |  |  |  |  |  |  |  |  |
| **Cisplatin** | **C** |  |  |  |  |  |  |  |  |  |  |  |  |  |  |  |  |  |  |  |  |
| **ATR Inhibitor** |  | **M** |  |  |  |  |  |  |  |  |  |  |  |  |  |  |  |  |  |  |  |

**Chemoradiotherapy dosing frequency escalation schema**

| **Dosing schedule**  **-1** |  | **Week 7** | | | | | | | **Week 8** | | | | | | | **Week 9** | | | | | | | **Week 10** | | | | | | | **Week 11** | | | | | | |
| --- | --- | --- | --- | --- | --- | --- | --- | --- | --- | --- | --- | --- | --- | --- | --- | --- | --- | --- | --- | --- | --- | --- | --- | --- | --- | --- | --- | --- | --- | --- | --- | --- | --- | --- | --- | --- |
|  |  | M | T | W | T | F | S | S | M | T | W | T | F | S | S | M | T | W | T | F | S | S | M | T | W | T | F | S | S | M | T | W | T | F | S | S |
|  | **Capecitabine** |  |  |  |  |  |  |  |  |  |  |  |  |  |  |  |  |  |  |  |  |  |  |  |  |  |  |  |  |  |  |  |  |  |  |  |
|  | **Cisplatin** | **C** |  |  |  |  |  |  |  |  |  |  |  |  |  |  |  |  |  |  |  |  | **C** |  |  |  |  |  |  |  |  |  |  |  |  |  |
|  | **Radiotherapy** | **X** | **X** | **X** | **X** | **X** |  |  | **X** | **X** | **X** | **X** | **X** |  |  | **X** | **X** | **X** | **X** | **X** |  |  | **X** | **X** | **X** | **X** | **X** |  |  | **X** | **X** | **X** | **X** | **X** |  |  |
|  | **ATR Inhibitor** |  | **M** |  |  |  |  |  |  | **M** |  |  |  |  |  |  | **M** |  |  |  |  |  |  | **M** |  |  |  |  |  |  | **M** |  |  |  |  |  |

| **Dosing schedule**  **1** |  | **Week 7** | | | | | | | **Week 8** | | | | | | | **Week 9** | | | | | | | **Week 10** | | | | | | | **Week 11** | | | | | | |
| --- | --- | --- | --- | --- | --- | --- | --- | --- | --- | --- | --- | --- | --- | --- | --- | --- | --- | --- | --- | --- | --- | --- | --- | --- | --- | --- | --- | --- | --- | --- | --- | --- | --- | --- | --- | --- |
|  |  | M | T | W | T | F | S | S | M | T | W | T | F | S | S | M | T | W | T | F | S | S | M | T | W | T | F | S | S | M | T | W | T | F | S | S |
|  | **Capecitabine** |  |  |  |  |  |  |  |  |  |  |  |  |  |  |  |  |  |  |  |  |  |  |  |  |  |  |  |  |  |  |  |  |  |  |  |
|  | **Cisplatin** | **C** |  |  |  |  |  |  |  |  |  |  |  |  |  |  |  |  |  |  |  |  | **C** |  |  |  |  |  |  |  |  |  |  |  |  |  |
|  | **Radiotherapy** | **X** | **X** | **X** | **X** | **X** |  |  | **X** | **X** | **X** | **X** | **X** |  |  | **X** | **X** | **X** | **X** | **X** |  |  | **X** | **X** | **X** | **X** | **X** |  |  | **X** | **X** | **X** | **X** | **X** |  |  |
|  | **ATR Inhibitor** |  | **M** |  |  | **M** |  |  |  | **M** |  |  |  |  |  |  | **M** |  |  |  |  |  |  | **M** |  |  | **M** |  |  |  | **M** |  |  |  |  |  |

| **Dosing schedule 2** |  | **Week 7** | | | | | | | **Week 8** | | | | | | | **Week 9** | | | | | | | **Week 10** | | | | | | | **Week 11** | | | | | | |
| --- | --- | --- | --- | --- | --- | --- | --- | --- | --- | --- | --- | --- | --- | --- | --- | --- | --- | --- | --- | --- | --- | --- | --- | --- | --- | --- | --- | --- | --- | --- | --- | --- | --- | --- | --- | --- |
|  |  | M | T | W | T | F | S | S | M | T | W | T | F | S | S | M | T | W | T | F | S | S | M | T | W | T | F | S | S | M | T | W | T | F | S | S |
|  | **Capecitabine** |  |  |  |  |  |  |  |  |  |  |  |  |  |  |  |  |  |  |  |  |  |  |  |  |  |  |  |  |  |  |  |  |  |  |  |
|  | **Cisplatin** | **C** |  |  |  |  |  |  |  |  |  |  |  |  |  |  |  |  |  |  |  |  | **C** |  |  |  |  |  |  |  |  |  |  |  |  |  |
|  | **Radiotherapy** | **X** | **X** | **X** | **X** | **X** |  |  | **X** | **X** | **X** | **X** | **X** |  |  | **X** | **X** | **X** | **X** | **X** |  |  | **X** | **X** | **X** | **X** | **X** |  |  | **X** | **X** | **X** | **X** | **X** |  |  |
|  | **ATR Inhibitor** |  | **M** |  |  | **M** |  |  |  | **M** |  |  | **M** |  |  |  | **M** |  |  | **M** |  |  |  | **M** |  |  | **M** |  |  |  | **M** |  |  | **M** |  |  |

## Management of M6620 (Berzosertib) drug administration

M6620 (Berzosertib) will be administered intravenously over 60 minutes (±10 minutes) approximately one hour after radiotherapy in stage A1 and B. When the total volume of infusion exceeds 600mL, the infusion may be extended by up to 30 minutes. Intravenous administration of M6620 (Berzosertib) is independent of food intake. When given after chemotherapy, the M6620 (Berzosertib) should be initiated approximately 24 hours (+/- 4hours) after cessation of cisplatin infusion (continue with Capecitabine as prescribed). The cannula should be removed following each infusion. Consideration of a Peripheral Inserted Catheter should be given if there are difficulties accessing a vein.

M6620 (Berzosertib) is associated with infusion-related reactions, e.g., infusion site erythema, infusion site reaction, and catheter site related reaction. Infusion reactions are common with IV administration of drugs used to treat cancer. These reactions occur during or shortly after administration of the drug and are diverse. They may include pruritus, flushing, chills/rigors, urticaria/rash, headache, bronchospasm/dyspnea, and hypotension or hypertension, among others. Infusion-related reactions, nausea, and vomiting are considered adverse drug reactions (ADRs) for MK6620.

The infusion can be mildly irritating so it is necessary to monitor the intravenous catheter site closely for evidence of erythema, tenderness or induration. To minimize the possibility of phlebitis, M6620 (Berzosertib) should be administered through a large bore catheter into a large caliber peripheral vein. The intravenous infusion site should be monitored closely for the development of erythema, induration, purulence, tenderness, or warmth. If any subject develops phlebitis, or signs or symptoms of inflammation that may progress to phlebitis or that the patient cannot tolerate, standard measures should be employed to ameliorate these symptoms (including removal of the infusion catheter and resumption of infusion through a different vein).

If any subject develops pruritus, flushing, or any other symptom suggestive of a systemic infusion reaction, standard measures should be employed to manage these symptoms (e.g. antihistamines and/or steroids, fluid support). Any non-serious reaction such as pruritis, can be prevented in subsequent administration by appropriate desensitizing measures prior to the administration of the study drugs as long as they are not prohibited by protocol (e.g. Corticosteroid and antihistamine combinations that may be used include: 100 mg to 200 mg hydrocortisone intravenously approximately 60 minutes (± 15 minutes) before M6620 (Berzosertib) infusion, and either 10 mg of chlorphenamine intravenously approximately 30 minutes (± 10 minutes) before M6620 (Berzosertib) infusion. Alternative antihistamine and steroid doses, timing, routes of administration and agents may be considered, as long as not prohibited by protocol. In addition, treatment with an H2-blocker (e.g., ranitidine) may be considered for subjects not responsive to a regimen with an H1‑blocker. If standard procedures to limit symptoms of an infusion reaction have failed, further re-challenges are prohibited.

Serious acute hypersensitivity reactions have occurred in a few subjects receiving M6620 (Berzosertib). These reactions occurred within minutes of re-exposure to M6620 (Berzosertib), and in cases reported to date, they have occurred during the second infusion. They may include hypotension and mental status changes. All subjects have fully recovered with standard treatment for this reaction, including immediate discontinuation of the inciting infusion and administration of IV corticosteroid and antihistamine, as well as IV fluids and oxygen when clinically indicated. If a serious acute hypersensitivity reaction occurs M6620 (Berzosertib) should be permanently withdrawn and reported as an SAR. For management of M6620 (Berzosertib) toxicity or missed doses see section 9.3.

**Laboratory values required for administration of M6620 (Berzosertib) and chemotherapy**

| Laboratory parameter | Cycle 1 Day 1 | Day 1 of subsequent cycles | Other days M6620 (Berzosertib) administered alone or with Capecitabine |
| --- | --- | --- | --- |
| Haemoglobin | ≥8.0g/dL Stage A1; ≥10.0g/dL Stage A2/B | ≥8.0g/dL Stage A1/A2; ≥10.0g/dL Stage B(during radiotherapy) | ≥7.0g/dL (if asymptomatic) |
| Absolute neutrophil count | ≥1.5 X 10^9^/L | ≥1 X 10^9^/L | ≥1.0 X 10^9^/L |
| Platelet count | ≥100 X 10^9^/L | ≥75 X 10^9^/L | ≥75 X 10^9^/L |
| AST/ALT | ≤2.5 X ULN or  ≤5 X ULN if liver metastases | ≤2.5 X ULN or  ≤5 X ULN if liver metastases | ≤2.5 X ULN or  ≤5 X ULN if liver metastases or capecitabine given alone |
| Estimated glomerular filtration rate | ≥40mL/min A1 or  ≥60mL*/min Stage A2/B | ≥60mL/min Stage A2/B | Not required |
| Total Bilirubin (serum) | ≤1.5 X ULN^a^ | Not required | Not required |

a – unless subject has known or suspected Gilbert’s syndrome

* if below 60ml/min, formal Creatinine clearance required (EDTA glomerular function rate, or 24 hours urine collection), and 100% dose cisplatin may be administered if formal result is ≥50ml/min

## Management of capecitabine administration

Patients will be instructed to take capecitabine tablets at a dose of 625mg/m^2^ twice a day as per standard practice starting in the evening of day 1 of each cycle (stage A2 and B). For patients who find swallowing capecitabine difficult, it is possible to dissolve the tablets in lukewarm water. The capecitabine tablets should be placed in approximately 200ml of lukewarm water. By stirring for about 15 minutes the tablets should dissolve. There is no stability data for any form of capecitabine suspension, so this should be done immediately prior to use and the solution swallowed immediately, rinsing to ensure all of the contents are ingested. As the solution will have a bitter taste it could be flavoured with a fruit juice or squash, but grapefruit juice should not be used. The solution may also be administered through a naso-gastric tube or other enteral feeding tube. Please note that dissolving capecitabine is outside of its licensed indication.

In addition, it is recommended that patients are carefully monitored for ophthalmologic complications, such as keratitis and corneal disorders, especially if they have a prior history of eye disorders. Treatment of eye disorders should be initiated promptly. For patients with diabetes mellitus, caution must be exercised as it may be aggravated.

**Concurrent chemoradiotherapy (Weeks 7-11)**

Patients should be instructed to take the drug twice daily as above **only** on the days radiotherapy is delivered.

Where the start of radiotherapy is delayed for scheduling reasons Day 1 of the third cycle of chemotherapy, i.e. Day 1 of the start of concurrent chemotherapy, should also be delayed such that the 2 treatments start together.

**Additional dosing requirements are:**

- Missed doses/dosing day will not be made up. The next dose must be taken as scheduled. The missed tablets should be brought at next clinic visit to be handed over to the research nurse.
- If a patient vomits after taking the tablets, they should not take another dose. The next doses should be taken as scheduled.
- Doses should NOT be doubled to make up for missed doses.
- Any unused tablets should be returned to pharmacy (via research nurse). All patients are asked to keep a record of their capecitabine use in their diary card.

## Management of cisplatin administration

Cisplatin will be administered on day 1 of a 3 weekly cycle. From weeks 1 to 18 for Stage A2 and 1-11 for Stage B as an intravenous infusion at 60mg/m^2^ over 2 hours on treatment days or as per local policy. The hydration regimen for cisplatin administration will be determined by locally agreed pharmacy procedures and guidelines. Pre and post anti-emetics should be given according to local practice for high risk emesis. Laboratory values required for administration of chemotherapy will be as per standard policy (for administration of M6620 (Berzosertib) see section 8.4). Patient monitoring and management of hypersensitivity and extravasation will be as per local hospital policy. Patients with significant hearing impairment will be made aware of potential ototoxicity. For those who choose to be included, it is recommended that audiograms be carried out at baseline and prior to cycle 2. Gastric protection (Proton Pump inhibitor or H2 blocker) is recommended for all patients for duration of therapy and for at least 6 weeks thereafter.

On days of concurrent chemo-radiation, cisplatin should be completed before radiotherapy treatment. Patients may undergo their radiotherapy during the post-hydration following cisplatin, which may be interrupted provided it is completed afterwards.

## Managing delays to Chemotherapy and Radiotherapy

Where the start of radiotherapy is delayed for scheduling reasons Day 1 of the third cycle of chemotherapy, i.e. Day 1 of the start of concurrent chemotherapy, should also be delayed such that the 2 treatments start together. The decision as to the scheduling of chemotherapy as a result of delays to radiotherapy due to machine service days or breakdowns i.e. unscheduled interruptions to radiotherapy should be made at the clinical discretion of the local PI, although the first treatment of radiotherapy must be given on the same first day of cycle 3 of the chemotherapy schedule. Management of hypersensitivity and extravasation will be as per local hospital policy.

## Laboratory values required for administration of chemotherapy

See table in section 8.4**.**

## Calculating and recalculating doses

The dose of M6620 (Berzosertib) will be calculated for each patient based on actual weight. BSA will be calculated according to the DuBois and DuBois formula. The patient’s weight should be recorded prior to every chemotherapy or chemoradiotherapy cycle to determine dose of chemotherapy. If a patient’s weight changes by ≥ 10% from baseline then drug doses should be recalculated. If a patient’s weight changes by <10% the dose may be adjusted according to local policy/clinician’s discretion, but is not an absolute requirement.

## Chemotherapy Dose-banding

Dose banding will be permitted as per local hospital policy (assumes dose banding is within 5% of actual calculated dose). The Trials Office will request each site to state upfront whether or not dose banding will be used.

## Dose capping

There will be no dose capping.

## Compliance

Patients will be instructed to keep a record of compliance in terms of their capecitabine treatment, by means of using a study patient diary card provided to the patient by the site. Patients should be asked to bring completed diary cards or other records and all their unused / remaining capecitabine tablets (empty, open or unopened) with them to each clinic visit. Sites should count remaining capecitabine at each visit. The patient diary cards should not be sent to the Trial Office but kept by the centre to monitor patient drug compliance. Compliance of M6620 (Berzosertib) and Cisplatin will be monitored by the patient record.

Accountability logs are required for capecitabine to determine that patients have received at least 80% of the prescribed treatment dose. Returns should be reconciled against the patient diary and the reason for any discrepancy documented. Site staff will collect and count patient returns which must be recorded on the drug accountability log.

## Management of overdose

Overdose of M6620 (Berzosertib) can result in an increase in the severity of M6620 (Berzosertib) toxicities (section 9.3.2). Medical management of overdose should include stopping the chemotherapeutic agents and customary therapeutic and supportive medical interventions aimed at correcting the presenting clinical manifestations and preventing their possible complications. An overdose of cisplatin or capecitabine should be managed as per standard policy.

# toxicity Management

## Dose Limiting Toxicity

## Reporting a DLT

A dose limiting toxicity is a toxicity that is considered related to M6620 (Berzosertib) or the interaction between M6620 (Berzosertib) and radiotherapy or chemoradiotherapy. **Dose limiting toxicities should be reported within 24 hours of the site becoming aware using the SAE form and scan and email as a PDF attachment to** [**octo-safety@oncology.ox.ac.uk**](mailto:octo-safety@oncology.ox.ac.uk) **and send an email notification to octo-CHARIOT@oncology.ox.ac.uk.** For management of M6620 (Berzosertib) toxicities see section 9.3. Chemotherapy and radiotherapy toxicities should be managed as per standard practice.

## Definition of a DLT

**Stage A1**

DLTs will be defined as per NCI CTCAE v4.03 and include:

- Absolute neutrophil count (ANC) <0.5 x 10^9^/L for >7days
- Febrile neutropenia (fever of unknown origin without clinically or microbiologically documented infection) (ANC <1.0 x 10^9^/L, fever >38.5^o^C) lasting >3 days
- Infection (documented clinically or microbiologically) with Grade 3 or 4 neutropenia (absolute neutrophil count <1.0 x 10^9^/L)
- Platelets <25 x 10^9^/L
- Clinically significant bleeding attributed to grade 3 thrombocytopenia or requiring platelet transfusion
- Grade ≥3 oesophagitis onset within 2 weeks of starting radiotherapy
- Grade ≥3 pneumonitis onset within 3 months of starting radiotherapy
- Grade ≥3 nausea or vomiting not controlled by optimal outpatient anti-emetic treatment
- Grade ≥3 diarrhoea despite optimal outpatient anti-diarrhoeal medication use
- Other grade 3 ≥ effects thought to be directly treatment related to the combination of M6620 (Berzosertib) with radiotherapy
- Any toxicity causing a delay of radiotherapy completion by greater than one week
- Missing 2 consecutive doses of M6620 (Berzosertib) within a cycle due to Grade ≥3 toxicity
- A delay of any of the 3 treatments of 7 days or more within a cycle due to treatment related toxicity
- An elevation of ALT or AST >5 x ULN lasting 8 days or more
- A concurrent elevation of ALT or AST >3 × ULN and total bilirubin >2 × ULN in whom there is no evidence of biliary obstruction or other causes that can reasonably explain the concurrent elevation
- Death due to drug related complications
- Cardiac:
- QTc prolongation (any QTc interval ≥500 msec or any change in QTc interval ≥60 msec from baseline) on ECG, unless related to an electrolyte abnormality and prolongation resolves with correction of electrolyte abnormality
- Any of the following (CTCAE criteria): Grade 2 or greater ventricular arrhythmia (second or third degree AV block), severe sustained/symptomatic sinus bradycardia less than 45 beats per minute (bpm) or sinus tachycardia >120 bpm not due to other causes (e.g., fever), persistent supraventricular arrhythmia (e.g., uncontrolled/new atrial fibrillation, flutter, atrioventricular nodal tachycardia, etc.) lasting more than 24 hours, ventricular tachycardia defined as >9 beats in a row or any length of torsades de pointes (polymorphic ventricular tachycardia with long QTc), or unexplained recurrent syncope
- Symptoms suggestive of congestive heart failure with confirmed Ejection Fraction (EF) <40% (by 2D-echocardiogram or Multiple Gated Acquisition [MUGA] scan)
- Troponin T: level which is consistent with myocardial infarction

**Stage A2**

DLTs will be defined as per NCI CTCAE v4.03 and include:

- Absolute neutrophil count (ANC) <0.5 x 10^9^/L for >7days
- Febrile neutropenia (fever of unknown origin without clinically or microbiologically documented infection) (ANC <1.0 x 10^9^/L, fever >38.5^o^C) lasting >3 days
- Infection (documented clinically or microbiologically) with Grade 3 or 4 neutropenia (absolute neutrophil count <1.0 x 10^9^/L)
- Platelets <25 x 10^9^/L
- Clinically significant bleeding attributed to grade 3 thrombocytopenia or requiring platelet transfusion
- Grade ≥3 nausea or vomiting not controlled by optimal outpatient anti-emetic treatment
- Grade ≥3 diarrhoea despite optimal outpatient anti-diarrhoeal medication use
- Other grade ≥3 effects thought to be directly treatment related to the combination of M6620 (Berzosertib) with chemotherapy
- Missing 2 consecutive doses of M6620 (Berzosertib) within a cycle due to Grade ≥3 toxicity
- A delay of any of the 3 treatments of 7 days or more within a cycle due to treatment related toxicity
- An elevation of ALT or AST >5 x ULN lasting 8 days or more
- A concurrent elevation of ALT or AST >3 × ULN and total bilirubin >2 × ULN in whom there is no evidence of biliary obstruction or other causes that can reasonably explain the concurrent elevation
- Death due to drug related complications
- Cardiac:
- QTc prolongation (any QTc interval ≥500 msec or any change in QTc interval ≥60 msec from baseline) on ECG, unless related to an electrolyte abnormality and prolongation resolves with correction of electrolyte abnormality
- Any of the following (CTCAE criteria): Grade 2 or greater ventricular arrhythmia (second or third degree AV block), severe sustained/symptomatic sinus bradycardia less than 45 beats per minute (bpm) or sinus tachycardia >120 bpm not due to other causes (e.g., fever), persistent supraventricular arrhythmia (e.g., uncontrolled/new atrial fibrillation, flutter, atrioventricular nodal tachycardia, etc.) lasting more than 24 hours, ventricular tachycardia defined as >9 beats in a row or any length of torsades de pointes (polymorphic ventricular tachycardia with long QTc), or unexplained recurrent syncope
- Symptoms suggestive of congestive heart failure with confirmed Ejection Fraction (EF) <40% (by 2D-echocardiogram or Multiple Gated Acquisition [MUGA] scan)
- Troponin T: level which is consistent with myocardial infarction

**Stage B**

Acute DLTs will be defined as per NCI CTCAE v4.03 and include:

- Absolute neutrophil count (ANC) <0.5 x 10^9^/L for >7days
- Febrile neutropenia (fever of unknown origin without clinically or microbiologically documented infection) (ANC <1.0 x 10^9^/L, fever >38.5^o^C) lasting >3 days
- Infection (documented clinically or microbiologically) with Grade 3 or 4 neutropenia (absolute neutrophil count <1.0 x 10^9^/L)
- Platelets <25 x 10^9^/L
- Clinically significant bleeding attributed to grade 3 thrombocytopenia or requiring platelet transfusion
- Grade ≥3 oesophagitis onset during induction chemotherapy or within 2 weeks of starting radiotherapy
- Grade ≥3 pneumonitis onset within 3 months of starting radiotherapy
- Grade ≥3 nausea or vomiting not controlled by optimal outpatient anti-emetics
- Grade ≥3 diarrhoea despite optimal outpatient anti-diarrheal medication use
- Other grade 3 ≥ effects thought to be directly treatment related to the combination of M6620 (Berzosertib) with chemotherapy or radiotherapy
- Any toxicity causing radiotherapy delivery delay by >3 consecutive fractions
- An elevation of ALT or AST >5 x ULN lasting 8 days or more
- A concurrent elevation of ALT or AST >3 × ULN and total bilirubin >2 × ULN in whom there is no evidence of biliary obstruction or other causes that can reasonably explain the concurrent elevation
- Missing 2 consecutive doses of M6620 (Berzosertib) within a cycle due to Grade ≥3 toxicity
- A delay of any of the 3 treatments of 7 days or more within a cycle due to treatment related toxicity
- Death due to drug related complications
- Cardiac:
- QTc prolongation (any QTc interval ≥500 msec or any change in QTc interval ≥60 msec from baseline) on ECG, unless related to an electrolyte abnormality and prolongation resolves with correction of electrolyte abnormality
- Any of the following (CTCAE criteria): Grade 2 or greater ventricular arrhythmia (second or third degree AV block), severe sustained/symptomatic sinus bradycardia less than 45 beats per minute (bpm) or sinus tachycardia >120 bpm not due to other causes (e.g., fever), persistent supraventricular arrhythmia (e.g., uncontrolled/new atrial fibrillation, flutter, atrioventricular nodal tachycardia, etc.) lasting more than 24 hours, ventricular tachycardia defined as >9 beats in a row or any length of torsades de pointes (polymorphic ventricular tachycardia with long QTc), or unexplained recurrent syncope
- Symptoms suggestive of congestive heart failure with confirmed Ejection Fraction (EF) <40% (by 2D-echocardiogram or Multiple Gated Acquisition [MUGA] scan) or a relative decrease >20% from screening assessment of EF or if performed within 12 months
- Troponin T: level which is consistent with myocardial infarction

**Late DLTs – onset 6 weeks after completion of radiotherapy will be defined as per RTOG/EORTC late toxicity and Mellow Dysphagia score (Appendix C, D):**

- Grade ≥3 lung and heart toxicity
- Grade 3 oesophageal toxicity with an increase in Mellow score by ≥2 since baseline (need to exclude disease progression on CT and/or endoscopy and biopsy)
- Grade 4 oesophageal toxicity

**Notes:**

- In the event of a Grade 4 neutropenia, a full blood count must be performed no more than 7 days after the onset of the event to determine if a DLT has occurred. Continue to monitor the subject closely until resolution to Grade 3 or less.
- In the event of a Grade 3 or higher elevation in ALT or AST, follow-up laboratory assessments should be performed every 48 to 72 hours until reduced to Grade 2 or less.

## Management of treatment toxicities

In response to a toxicity of unknown or indeterminate causality M6620 (Berzosertib) treatment should be primarily withheld followed by chemotherapy. In the case of M6620 (Berzosertib) or chemotherapy toxicity or dose modification during the concurrent chemoradiotherapy phase patients should continue with RT where possible. The decision as to whether to continue radiotherapy is at the discretion of the treating clinician. If toxicity could be attributable to systemic therapy or radiotherapy, systemic therapy should be withheld first (see section 12.8 for management of radiotherapy toxicities).

**Appropriate dose modifications should be considered for all agents if grade 3 or 4 toxicity occurs (see section 9.3 and 9.4 below). In stage A2 dose reductions should not be made beyond 50% of starting dose and the patient should come off study if further Grade 3 or 4 non-haematologic toxicity or Grade 4 haematologic toxicity is then experienced.**

## Management of M6620 (Berzosertib) drug toxicities

No dose modifications of M6620 (Berzosertib) may be made (see 9.3.1 for exception in stage A2 below). Treatment may be interrupted because of a non-DLT of Grade 3 or higher, at the discretion of the Investigator. Treatment may be resumed when all toxicities have returned to grade 2 or less, at the discretion of the Investigator. If two consecutive doses of M6620 (Berzosertib) within a cycle are missed due to M6620 (Berzosertib) related Grade ≥3 toxicity, this constitutes a DLT, and no further M6620 (Berzosertib) will be administered. Missed doses due to ≤Grade 2 events, will be classified as non-compliance, not a DLT. A delay of any of the 3 treatments of 7 days or more within a cycle due to treatment related toxicity would be considered a DLT.

If the subject misses a dose of M6620 (Berzosertib) for any reason other than toxicity the dose should not be made up and the scheduling should continue as normal, from the next planned dose of M6620 (Berzosertib), unless it is a Tuesday dose which can be made up on the following day (Wednesday). If radiotherapy is withheld for toxicity or other reasons, M6620 (Berzosertib) should also be withheld and the dosing schedule should continue from the next planned dose once radiotherapy has resumed (see also section 8.4).

In Stage A2, if a patient experiences a DLT, they may continue on trial treatment at the next lowest dose level at the discretion of the treating investigator.

In Stage B, if a patient experiences a DLT, the TMG will convene to decide whether it is appropriate for the patient to continue receiving trial treatment, potentially at a reduced frequency of doses.

## Additional guidance Stage A2

In stage A2 M6620 (Berzosertib) must not be dose reduced in the first 4 weeks of treatment but doses may be omitted for toxicity (as above). The M6620 (Berzosertib) dose may be reduced after the 4 week DLT window is complete as follows:

1.     For Grade 4 hematologic toxicity: dose of M6620 (Berzosertib) to be reduced by 25%.

2.     For Grade 3 non-hematologic toxicity: dose of M6620 (Berzosertib) to be reduced by 25%.

3.     For Grade 4 non-hematologic toxicity: dose of M6620 (Berzosertib) to be reduced by 50%.

If the dose of M6620 (Berzosertib) has already been reduced by 25% a further dose reduction of 25% may be made to 50% of starting dose. However no further dose reductions may be made beyond 50% of starting dose and the patient should come off study if further Grade 3 or 4 non-haematologic toxicity or Grade 4 haematologic toxicity is then experienced.

## Toxicities associated with M6620 (Berzosertib)

Acute hypersensitivity reactions are a toxicity of M6620 (Berzosertib). Serious acute hypersensitivity reactions have occurred during the second M6620 (Berzosertib) infusion in approximately 5% of subjects administered M6620 (Berzosertib) and should be reported as an Serious adverse reaction (see section 8.4 for management of hypersensitivity reactions). In at least one sixth of patient reactions at the site of infusion were noted including erythema, swelling, pruritus. The most common toxicities reported in patients who have received M6620 (Berzosertib) are: fatigue, abdominal pain, diarrhoea, nausea and vomiting, decreased appetite, cough, headache and fever. Almost all instances of these toxicities occurred in patients who were also receiving chemotherapy in combination with M6620 (Berzosertib). M6620 (Berzosertib) in combination with chemotherapy may exacerbate the risks associated with the chemotherapy. The most frequently noted grade ≥ 2 laboratory abnormalities observed (in combination with chemotherapy) are neutropenia, low lymphocytes, low haemoglobin and elevated ALT/AST. M6620 (Berzosertib) absorbs UV-visible radiation spectrum and is widely distributed including to the skin, so subjects should be cautioned to minimise exposure to the sun and other sources of visible and UV radiation and to take protective measures when necessary. Full range of toxicities can be found in the M6620 (Berzosertib) IB.

## Management of chemotherapy toxicities

Toxicity due to capecitabine or cisplatin administration may be managed by symptomatic treatment and/or modification of the dose (treatment interruption or dose reduction) as per standard of care. Once the dose has been reduced, it should not be increased at a later time. Patients taking capecitabine should be informed of the need to interrupt treatment immediately if moderate or worse toxicity occurs. Detailed information can be found in the product SPCs available from [www.medicines.org.uk](http://www.medicines.org.uk) .

## Haematological toxicity

Myelosuppression is uncommonly observed with cisplatin and capecitabine. Neutropaenia and thrombocytopaenia should be monitored according to the recommended protocol and appropriate dose modifications made. Anaemia may occur cumulatively with cisplatin and should be corrected during radiotherapy to maintain the haemoglobin ≥10g/dL. The FBC should be taken and reviewed (up to 3 days) prior to Day 1 of each cycle of chemotherapy.

**Dose modification for haematological toxicities**

| **Neutrophil / platelet count (10^9^/L)**  **(day 1 of subsequent cycles)** | **Action** |
| --- | --- |
| ANC ≥ 1 and/or plts ≥ 75 | Full dose drugs |
| ANC 0.5- <1 and/or plts 50- <75 OR any episode of neutropenic sepsis during the previous cycle | Stop chemotherapy until recovery.  Restart with 25% dose reduction cisplatin and capecitabine |
| ANC <0.5 and/or plts <50 | Stop chemotherapy until recovery.  Restart with 50% dose reduction cisplatin and capecitabine |

## Non-haematological toxicity

**Gastrointestinal toxicity:**

Nausea and vomiting is common following cisplatin, usually starting within 1 hour of treatment and lasting up to 24 hours. Anorexia, nausea and occasional vomiting may persist for up to one week. Nausea occurs less commonly with capecitabine (Diarrhoea occurs with capecitabine and patients should receive advice regarding discontinuation of therapy and use of loperamide or codeine phosphate). Clinicians should be aware of infective causes of diarrhoea (e.g. *Clostridium difficile)*, and patients should be tested in cases of concern. Antibiotic treatment is not recommended routinely but may be required in such circumstances. Stomatitis occurs with capecitabine and patients should receive advice regarding good oral care, and the use of mouthwash (e.g. Corsodyl™).

**Anaphylaxis:** Reactions to cisplatin therapy have been occasionally reported in patients who were previously exposed to cisplatin. Patients who are particularly at risk are those with a prior history or family history of atopy. Serious reactions may be controlled by IV adrenaline, corticosteroids or antihistamines.

**Serum Electrolyte Disturbances:** Hypomagnesaemia, hypocalcaemia, hyponatraemia, hypokalaemia and hypophosphataemia have been reported to occur in patients treated with cisplatin and hypomagnesemia may occur and should be monitored according to the protocol.

**Other Toxicities:** Hair loss is not expected with this combination but may rarely occur with most chemotherapeutic agents. Vascular toxicities coincident with the use of cisplatin in combination with other antineoplastic agents have been reported rarely. These events may include myocardial infarction, cerebrovascular accident, thrombotic microangiopathy (haemolytic uraemic syndrome) or cerebral arteritis. There have been reports of optic neuritis, papilloedema and cerebral blindness following treatment with cisplatin.

| **Non-haematological toxicity dose reductions for Capecitabine and Cisplatin Toxicity** | During a course of therapy – Cisplatin and Capecitabine | Dose adjustment for next cycle (% of starting dose) |
| --- | --- | --- |
| Grade 1 | Maintain dose level for both drugs | 100% Cisplatin & Capecitabine |
| Grade 2 | | |
| 1^st^ appearance | Interrupt until resolved to grade 0-1 | 100% Cisplatin & Capecitabine |
| 2^nd^ appearance | Interrupt until resolved to grade 0-1 | 75% Cisplatin & Capecitabine |
| 3^rd^ appearance | Interrupt until resolved to grade 0-1 | 50% Cisplatin & Capecitabine |
| Grade 3 | | |
| 1^st^ appearance | Interrupt until resolved to grade 0-1 | 75% Cisplatin & Capecitabine |
| 2^nd^ appearance | Interrupt until resolved to grade 0-1 | 50% Cisplatin & Capecitabine |
| 3^rd^ appearance | Discontinue treatment permanently |  |
| Grade 4 | | |
| 1^st^ appearance | Discontinue permanently  *Or*  If physician deems it to be in the patient’s best interest to continue, interrupt until resolved to grade 0-1 after discussion with Chief Investigator | 50% Cisplatin & Capecitabine |

## Additional capecitabine toxicities

The following are the recommended dose modifications for toxicity. In addition patients should receive loperamide in case of diarrhoea and mouthwashes and anti-emetics according to local policy. Toxicities should be graded according to CTCAE v4.03. In particular, diarrhoea, nausea, vomiting, stomatitis and skin reactions are to be noted. Please use the alternative specific toxicity for Hand-foot syndrome (PPE), the frequency of which in patients receiving capecitabine has led to altered toxicity ratings.

**Nephrotoxicity**

| GFR (mls/min) Baseline and prior to Day 1 | Capecitabine dose |
| --- | --- |
| ≥ 50ml/min | 100% |
| 30-49 ml/min | 75% |
| < 30ml/min | This group of patients should be withdrawn from trial treatment (please complete withdrawal form at Level 1) and treated according to local investigator choice and continue to complete CRFs |

| Grade of  hand-foot syndrome |  |
| --- | --- |
| 1 | Numbness, dysaesthesia/paraesthesia, tingling, painless swelling or erythema of the hands and/or feet and/or discomfort which does not disrupt the patient's normal activities. |
| *2* | Painful erythema and swelling of the hands and/or feet and/or discomfort affecting the patient's activities of daily living. |
| 3 | Moist desquamation, ulceration, blistering and severe pain of the hands and/or feet and/or severe discomfort that causes the patient to be unable to work or perform activities of daily living. |

If grade 2 or 3 hand-foot syndrome occurs, administration of capecitabine should be interrupted until the event resolves or decreases in intensity to grade 1. Following grade 3 hand-foot syndrome, subsequent doses of capecitabine should be decreased.

**Chest pain**

For patients with a history of angina please ensure they have GTN spray at home and remain on their cardiac medication. If unexplained chest pain occurs on treatment, capecitabine should be stopped, an ECG performed and cardiac enzymes measured. In the case of angina or myocardial infarction being confirmed this should be managed according to usual local practice. Patients should not recommence capecitabine therapy and further therapy should be discussed with the Chief Investigator. Such cardiac toxicity should be reported through a SAE form.

**Hepatotoxicity**

Isolated elevation of serum transaminases may be related to capecitabine and will not require dose interruption unless AST/ALT levels are ≥ 5 times ULN. If AST/ALT is above this level, capecitabine will be interrupted till it returns to ≤ 2.5 times ULN.

**DPD deficiency**

If a patient has not received capecitabine in the past DPD testing should be undertaken as per institutional protocol. Patients with partial or full DPD deficiency are not eligible for the study.

Occasionally (approximately 1-3%) a patient may have a markedly exaggerated toxicity due to reduced 5FU catabolism. If this occurs, await full recovery of toxicities. Further treatment should be discussed with the Chief Investigator or one of the clinical co-investigators.

## Additional Cisplatin toxicities

**Nephrotoxicity**

Cisplatin produces cumulative nephrotoxicity. If a baseline estimate of renal function using the Cockroft & Gault formula predicts the GFR to be ≥ 60mL/min full dose cisplatin should be used. If the estimate is <60mls/min a formal measure of Creatinine clearance should be performed (EDTA or 24 hour urine test) and the appropriate cisplatin dose used (see table below). In the case of a 25% deterioration in estimated renal function (using the Cockroft & Gault formula) on pre-treatment blood samples a formal Creatinine clearance test should be performed and pending this an appropriate dose reduction in cisplatin should be made. The formal Creatinine clearance result, when available, takes precedent over estimated GFR for subsequent cisplatin dose calculations.

| GFR (mls/min) Baseline and prior to Day 1 | Cisplatin Dose |
| --- | --- |
| ≥ 60ml/min | 100% |
| 45-59ml/min | 50% |
| 30-44 ml/min | Stop Cisplatin. This group of patients if in dose level 1 should be withdrawn from trial treatment (please complete withdrawal form at Level 1) and treated according to local investigator choice and continue to complete CRFs. |
| < 30ml/min | This group of patients should be withdrawn from trial treatment (please complete withdrawal form at Level 1) and treated according to local investigator choice and continue to complete CRFs |

**Neurotoxicity/ototoxicity**

Neurotoxicity/ototoxicity appears to be cumulative. Prior to each course, any new or progressive symptoms of peripheral neuropathy should be established.

# Other Treatments (NON-IMPs)

## Background systemic therapy

The chemotherapy agents cisplatin and capecitabine are not licensed for use in oesophageal cancer but are used as the standard treatment for oesophageal cancer in the UK. Details of dose administration and toxicity management can be found in the SPC, standard practices and policies apply throughout (Stage B). However, cisplatin and capecitabine are not considered standard practice in stage A2 & B therefore these drugs are considered IMPs for the purpose of this trial.

## Support medication

Pre-medication and supportive medication should be given as per local practice.

## Concomitant medication and non-drug therapies

Concomitant medication may be given as medically indicated. All patients will be asked to provide a complete list of prescription and over-the-counter medications that have been taken within the previous 4 weeks prior to the first treatment visit. They must also inform the Investigator about any new medication started while in the trial. Details (including indication, doses, frequency and start / stop dates) of concomitant medication taken during the trial until the completion of the off-study visit must be recorded in the medical record and the appropriate CRF.

## Prohibited therapies

Patients should not be prescribed any other anti-cancer or investigational therapies while participating in this study. In addition, the following should be noted.

- Radiation therapy is permitted as per schedule.
- Oesophageal stent is not permitted.
- Live vaccine is not permitted within 30 days prior to treatment start, for the duration of trial treatment and for 6 weeks after the last administration of IMP dose.

## Potential Drug Interactions

## M6620 (Berzosertib)

Drug interaction profile of M6620 (Berzosertib) has not been fully characterised, caution should be used when co-administering medications with M6620 (Berzosertib). Based on its mechanism of action, M6620 (Berzosertib) may increase the frequency or severity of adverse reactions associated with cisplatin.

As M6620 (Berzosertib) is primarily metabolised by CYP3A4, concomitant administration with potent inhibitors or potent inducers of CYP3A4 should be avoided. Up to 14 days prior to receiving study drug and through the duration of the study, the following list of potent CYP3A4 inhibitors or inducers should be avoided. Please note that the following list is not exhaustive:

**Potent CYP3A4 inhibitors**: Clarithromycin, itraconazole, ketoconazole, mibefradil, hepatitis C virus and HIV protease inhibitors, nefazodone, posaconazole, telithromycin, voriconazole

**Potent CYP3A4 inducers**: carbamazepine, rifampicin, rifapentine, phenobarbital, phenytoin, primidone, St John’s wort

Grapefruit/grapefruit juice, Seville or blood oranges or marmalade – none allowed within 14 days before first dose or during treatment period with M6620 (Berzosertib).

**Haemopoetic growth factors** - none allowed within 14 days before first dose or prophylactic use with cycle 1.

## Capecitabine and Cisplatin

Capecitabine and Cisplatin interact with several medications and the following precautions should be followed:

Drugs to be avoided:

- Cumulative nephrotoxicity may be potentiated by aminoglycoside antibiotics e.g. gentamicin. These should not be administered, if possible, simultaneously or 1-2 weeks after treatment with Cisplatin
- Thymine antivirals and analogues, including Brivudine (would require a 4-week wash out prior to entering in the trial)
- Methotrexate, Bleomycin - Reduce renal excretion of bleomycin and methotrexate which increases their toxicity
- Anti-gout agents (like allopurinol, colchicine, probenecid or sulfinpyrazone) - reduce the efficacy of Capecitabine
- Warfarin - Coumarin derivative anticoagulants (like warfarin) require more frequent monitoring due to altered coagulation parameters, and effects may occur up to several months after initiating Capecitabine therapy. A low molecular weight heparin can be used as a replacement during trial treatment.

The following drugs may require dose modification:

- Ototoxic drugs like aminoglycoside antibiotics or loop diuretics (e.g. furosemide), may increase ototoxic potential of Cisplatin.
- Anti-epileptics; the serum level of phenytoin may be reduced and levels should be monitored and the dose adjusted accordingly - may increase Capecitabine levels
- Folic acid/folinic acid – reduces maximum tolerated dose and may increase Capecitabine toxicity
- Aluminium hydroxide or magnesium hydroxide containing antacids – increase plasma concentrations of Capecitabine and its metabolite 5DFCR (5’-deoxy-5-flurocytidine)
- Interferon alpha – reduces maximum tolerates dose of Capecitabine
- Cytochrome p450 down regulation by Capecitabine may affect the following class of drugs – angiotensin II blockers (losartan, valsartan); oral hypoglycaemic agents (glipizide, tolbutamide, rosiglitazone); NSAIDS (indomethacin, celecoxib, diclofenac, ibuprofen)

Please note that the above contraindications are not exhaustive and investigators should refer to the SPC for full guidance [www.medicines.org.uk](http://www.medicines.org.uk).

# Drug management

All details regarding M6620 (Berzosertib) packaging, labelling and dispensing will be included in the Pharmacy Manual. Cisplatin and capecitabine should be managed as per local policies and procedures.

## Drug supplies

## M6620 (Berzosertib)

M6620 (Berzosertib) will be supplied by MERCK KGAA, DARMSTADT GERMANY in 10ml vials of a 20mg/ml solution for infusion. MERCK KGAA, DARMSTADT GERMANY will ship drug to Fisher Clinical Services where it will be labelled according to applicable regulatory requirements and QP released.

## Capecitabine and cisplatin

Cisplatin as a solution for injection and capecitabine as a tablet for oral use should be supplied from trial site’s own stock and funded locally.

## Drug ordering

Initial supplies of M6620 (Berzosertib) are sent out by Fisher Clinical Services after they have been informed by the Trial Office that all approvals are in place. Subsequent supplies will be ordered by the Trial office. Pharmacy should request additional shipments of M6620 (Berzosertib) using the drug order form provided. Email the completed form to the Trial Office

(octo-CHARIOT@oncology.ox.ac.uk).

Pharmacy is responsible for monitoring the M6620 (Berzosertib) stock and re-ordering when required. Complete drug orders received by the Trial Office before 4pm will arrive at the Pharmacy within 10 working days.

If a vial of M6620 (Berzosertib) is accidentally destroyed or damaged, i.e. by dropping the vial, damaged packaging or through contamination, the pharmacist should contact the Trial Office for replacement.

## IMP Receipt

A copy of each M6620 (Berzosertib) delivery note and temperature monitoring form should be **scanned and emailed as a PDF attachment to** [**octo-CHARIOT@oncology.ox.ac.uk**](mailto:octo-CHARIOT@oncology.ox.ac.uk)**.** The original should be kept in the Pharmacy File. If supplies are damaged on arrival contact the Trial Office. Damaged supplies should be destroyed on site and a Drug Destruction Log completed.

## Handling and storage

## M6620 (Berzosertib)

Unopened vials of single use sterile light protected M6620 (Berzosertib) should be stored at controlled room temperature (15^o^C to 30^o^C). It should be retained in the original package to protect from light. Shelf life as instructed on packaging. Following the preparation of diluted M6620 (Berzosertib), intravenous bags should be covered to protect from light and stored in the dark.

The Investigator or an authorised designee will ensure that all the investigational products are stored in a secured area, under recommended storage conditions and in accordance with applicable regulatory requirements. To ensure adequate records, all study drugs will be accounted for via drug accountability forms as instructed by the Trial Office.

## Capecitabine and cisplatin

Will be handled and stored as per the local practice.

## Labelling

Ensure the phrase “Keep out of reach of children” is used on all medications.

## M6620 (Berzosertib)

A 20mg/mL (10mL total volume) M6620 (Berzosertib) sterile solution will be supplied by Merck KGaA, Darmstadt Germany. The responsible Pharmacy will ensure that IMP supplies dispensed for trial use are appropriately labelled as per local practice for infusions in accordance with all applicable regulatory requirements.

## Capecitabine and cisplatin:

Labelling will be as per local practice.

## Dosing and dispensing

## M6620 (Berzosertib)

M6620 (Berzosertib) will be supplied as 20 mg/mL M6620 (Berzosertib) (in betadex sulfobutyl ether and acetate buffer) to be diluted in Glucose 5% IV infusion before intravenous infusion. M6620 (Berzosertib) solution will be constituted into the individual dosing containers by a qualified member of pharmacy staff. Details of dose preparation will be provided in the Pharmacy Manual (see Formulation Preparation Instructions).

M6620 (Berzosertib) will be administered intravenously over 60 minutes (±10 minutes). When the total volume of infusion exceeds 600mL, the infusion may be extended beyond 60 minutes (as tolerated), but no more than 90 minutes.

## Cisplatin and capecitabine

Refer to the Summary of Products Characteristics (SPC) for full prescribing information and details of drug reconstitution, administration and stability (<http://www.medicines.org.uk/emc/>). Mannitol may be given concurrently with cisplatin or may be given as a short infusion according to local policy.

## Drug accountability

Drug accountability is the responsibility of the site pharmacist listed on the trial delegation log. Full drug accountability records must be maintained for M6620 (Berzosertib), cisplatin and capecitabine. Hospitals may amend the Drug Accountability Logs provided or use their own documentation if it captures all the information requested on the Drug Accountability Logs and has been approved by the Trial Office in advance.

At the conclusion of the study the overall numbers of drug shipped to the centre, the number dispensed and the number destroyed or returned will be provided by the pharmacy. An account must be given of any discrepancy.

## Drug destruction

Chemotherapy drugs will be disposed of as per local hospital policy. Disposal of M6620 (Berzosertib) will be according to the table below.

| Used / partially used vials | Disposal at site according to local hospital policy. |
| --- | --- |
| Patient returns (Capecitabine only) | Disposal at site according to local hospital policy.  Documented on Drug Accountability Log |
| Expired drug | Any expired drug should be disposed of at site according to local hospital policy. A Drug Destruction Log should be completed. |
| Drug left unused | Once authorised to do so, any unused drug should be disposed of at site according to local hospital policy. A Drug Destruction Log should be completed. |

The original drug destruction logs should be placed in the Pharmacy File and a copy **scanned and emailed to**

[**octo-CHARIOT@oncology.ox.ac.uk**](mailto:octo-CHARIOT@oncology.ox.ac.uk)**.**

## Occupational safety

Vein extravasation and accidental spillages should be dealt with according to hospital policy. The product is not expected to pose an occupational safety risk to site staff under normal conditions of use and administration.

# Radiotherapy (or chemoradiotherapy)

## **Dose prescription and fractionation**

It is highly recommended that the radiotherapy will be delivered in a single phase, treating each field daily Monday to Friday and prescribed and recorded as per ICRU 50/62. Conformal radiotherapy with a pixel based inhomogeneity correction is essential. Photon energy should be between 6MV and 10MV (energies in excess of 10MV should only be used in exceptional cases due to secondary build-up depth.

**Stage A1**

Patients recruited to Stage A1 of CHARIOT will receive a planned single-phase treatment delivered with IMRT. If IMRT is not possible then a 3D Conformal approach (meeting the required constraints) should be used. The total dose of radiation will be 35Gy in 15 fractions treating once daily, 5 days a week Monday to Friday and prescribed and recorded as per ICRU 62.

**Stage B**

Patients recruited to Stage B of CHARIOT should have a single phase inverse-planned IMRT treatment plan produced and treatment delivered with multiple field static or rotational fields.

The total dose of radiation will be 50Gy in 25 fractions treating once daily, 5 days per week Monday to Friday and prescribed and recorded as per ICRU 62.

## **Radiotherapy localisation**

A contrast enhanced CT (CECT) and depending on the tumour location, this will be followed by 4DCT in treatment position, i.e. supine with their arms above their heads, must be acquired for RT planning. The 4DCT is not mandated for Stage A and imaging will be done as per local policy. Intravenous contrast should be used (providing adequate renal function), to help distinguish the GTV from surrounding tissues; but oral contrast should not be used as it is not helpful in most cases and may interfere with planning calculations. To enable accurate assessment of the doses to organs at risk (OAR) the scan should extend superiorly to at least one CT slice above the apices of the lungs and inferiorly to the iliac crest (L2). Scans for upper third tumours may need to extend superiorly to the tragus.

For Stage B, the planning scan should be performed as per local guidelines ideally within 2 weeks of starting the neoadjuvant phase of chemotherapy. CT slice thickness should be no greater than 3mm. It is recommended that all patients have a CT scan of the thorax, abdomen and pelvis and an endoscopic ultrasound (EUS), noting the full extent of the disease with reference to anatomical landmarks. PET has an established role in the UK in terms of staging oesophageal cancer and can be useful in determining the extent of the disease, but the volume as defined by CT and EUS should not be reduced based on PET findings alone.

## **Target volume definition (TVD)**

Patients will be divided into two separate groups according to the location of the *centre of the primary tumour*:

a) Proximal tumours (tumours of upper and middle 1/3 oesophagus) defined here as primary tumour whose midpoint is above 32cm ab oral (NB proximal extent of primary being below 15cm is an eligibility criteria).

b) Distal tumours (tumours of distal 1/3 of oesophagus and gastro-oesophageal junction (GOJ)), defined here as being eligible patients with tumours whose midpoint is below 32cm ab oral (NB distal extent of primary being less than 2cm from the GOJ is an eligibility criteria).

This distinction accounts for the need to manually outline the elective nodal regions below the GOJ for the distal tumours. There is also significant movement in this region due to respiration requiring a larger PTV margin. Where possible, the centres are encouraged to use 4DCT planning scans for distal tumours.

Targets are defined following the principles of ICRU 50 and 62. The target volumes are localised on axial slices of the planning 3DCT or 4DCT scan.

**Stage A1 - All tumours**

| Volume | Description |
| --- | --- |
| **GTV** | Consists of the primary tumour and involved nodes deemed treatable and the circumference of the oesophagus at the level of disease. GTV definition is aided by information from EUS, diagnostic spiral CT scan, barium studies and 18-FDG PET scan if available. Encompass ‘tumour’ seen on the planning CT even if outside the EUS defined disease extent i.e. the GTV should be the most proximal and distal extension of disease as seen on EUS or CT scan. The lateral and anterior-posterior GTV margins are derived from the planning CT scan. |
| **CTVA** | GTV is copied and labelled ‘CTVA’ and is grown manually to include the circumference of the oesophagus superiorly and inferiorly. The superior-inferior margin of CTVA will be 10mm from the edges of GTV. |
| **CTVB** | CTVA is copied and labelled ‘CTVB’. It is grown by adding 10mm in right-left and anterior-posterior directions using the Treatment Planning System (TPS). |
| **PTV** | CTVB is copied and labelled ‘PTV’ and is grown by adding 10mm in the superior – inferior and 7 mm circumferentially using the TPS (2mm Internal Margin, IM and 5mm Setup Margin, SM). |

**Stage B** **-** Proximal tumours

| Volume | Description |
| --- | --- |
| **GTVp** | Consists of the primary tumour and the circumference of the oesophagus at the level of disease. GTV definition is aided by information from EUS, diagnostic spiral CT scan, barium studies and 18-FDG PET scan. Encompass ‘tumour’ seen on the planning CT even if outside the EUS defined disease extent i.e. the GTV should be the most proximal and distal extension of disease as seen on EUS or CT scan. The lateral and anterior-posterior GTV margins are derived from the planning CT scan. |
| **GTVn** | Consists of involved nodes not in continuity with the tumour. These should be outlined separately and named separately e.g. GTVn1, GTVn2 etc. where more than one GTVn exist. Only the node should be outlined – it is not required to include the full circumference of the oesophagus. |
| **GTVpn** | Combine GTVp and all GTVn and the circumference of the intervening oesophagus. |
| **CTVA** | GTVp +  20mm sup-inf (manually grown along direction of oesophagus) OR GTVn + 10mm superior-inferior margin (If GTVn is more proximal/distal than GTVp). |
| **CTVB** | CTVB = CTVA + 10mm circumferentially (but not superior-inferior), edited for normal structures but NOT beyond  CTVC. Normal structures include lung, pericardium, large vessels, trachea, right and left main bronchi, liver and the vertebrae, both above and below the diaphragm. |
| **CTVC** | CTVA + 5mm circumferentially (but not superior-inferior) – this is the minimum expansion around CTVA and is respected when CTVB is edited. |
| **PTV** | CTVB is copied and labelled ‘PTV’ and is grown by adding 10mm in the superior – inferior and 7 mm circumferentially using the TPS (2mm Internal Margin, IM and 5mm Setup Margin, SM). Posteriorly this margin may be reduced if the PTV extends across the vertebrae by more than 5mm as the margin for internal movement is not required as tumour cannot move into vertebrae and only setup margin is required. |

**Stage B -** Distal tumours (3DCT)

| Volume | Description |
| --- | --- |
| **GTVp** | As for proximal tumour |
| **GTVn** | As for proximal tumour |
| **CTVA** | GTVp is copied and labelled ‘CTVA’ and is grown manually to include the circumference of the oesophagus superiorly and inferiorly by 20mm. |
| **CTVN** | GTVn is coped and labelled ‘CTVn’ and is grown by a 5mm margin in all directions (for multiple nodes, label CTVn1, CTVn2 etc). This defines a minimum margin around positive nodes to assist in the delineation of CTVB. |
| **CTVB** | CTVA is copied and labelled ‘CTVB’. It is grown by adding 10mm in right-left and anterior-posterior directions using the Treatment Planning System. CTVB is edited to exclude lung, pericardium, large vessels and the vertebrae. CTVA is copied and labelled CTVC. It is grown by adding 5 mm circumferential, this contour is then combined with CTVB to create CTVB1. *Below the GOJ* CTVB1 is grown manually to include the volume at risk to a total of 20mm below GTVp and at least 10mm below lowest GTVn. Therefore this volume should include CTVn and the elective nodal regions at high risk of microscopic spread (lymph node stations along the lesser curve to include the para-cardial, and left gastric lymph nodes along the lesser curve of the stomach). |
| **PTV** | CTVB1 is copied and labelled ‘PTV’ and grown 10mm superiorly and 7 mm circumferentially (2mm IM + 5mm SM) and 15mm inferiorly (10mm IM + 5mm SM) using the TPS. Posteriorly this margin may be reduced if the PTV extends across the vertebrae by more than 5mm as the margin for internal movement is not required as tumour cannot move into vertebrae and only setup margin is required. |

**Distal tumours (4DCT)**

The principle involved in 4D planning is to account for intra-fraction motion and therefore generate patient specific volumes. It is expected that sites will have detailed local guidance for acquisition and outlining on 4D dataset, which has been accredited as part of previous trial involvement. Briefly, GTVp. GTVn, CTVA and CTVB contours are generated (as per the distal tumour 3DCT protocol above) on the Maximum Inhalation scan, Maximum Exhalation scan and Reference scan of the 4DCT dataset resulting in the following volumes:

**Maximum inhalation scan - GTVp_MaxIn, GTVn_MaxIn, CTVA_MaxIn, CTVB_MaxIn**

**Maximum exhalation scan – GTVp_MaxEx, GTVn_MaxEx, CTVA_MaxEx, CTVB_MaxEx**

**Reference scan - GTVp_Ref, GTVn_Ref, CTVA_Ref, CTVB_Ref**

**ITV** is derived by combining CTV_MaxIn, CTVB_MaxEx and CTVB_Ref as the composite CTVB volumes, edited to account for any additional motion seen from all other 4DCT phases (i.e. the editing will result in the ITV only being made bigger, to account for the maximum extent of these motion effects). The above contours must be associated with the reference dataset.

**PTV** = ITV + SM = ITV + 5mm

**Set up margin (SM)** is applied for treatment inaccuracies, such as set up error, and for the purpose of this protocol ITV is grown by 5mm in all directions using the TPS. Following the generation of the PTV, outlining of the organs at risk structures and planning should be carried out on the reference data set only. Maximum length of PTV for Stage B = 15cm

## **Organs at risk**

The organs at risk (OARs) that must be contoured for CHARIOT and the naming convention used in naming these structures are detailed below. The spinal cord should be outlined on slices which include or are within 20mm of the PTV in the superior and inferior directions and a Planning Risk Volume (PRV) for the cord is created to account for positioning error. The full extent of the right and left lungs are outlined, this should be done in such a way that the planning system will be able to calculate a combined lung dose volume histogram (DVH). The whole heart is outlined to the extent of the pericardial sac (if visible). The major blood vessels (superior to the organ) and the inferior vena cava (towards the inferior extent of the heart) are excluded. The whole liver is outlined if the level of its superior edge overlaps with the level of the inferior extent of the PTV. Each kidney is outlined separately if the level of its superior edge overlaps with the level of the inferior extent of the PTV. The whole stomach should be outlined in such a way that a stomach DVH can be produced

## **Treatment Plan Optimisation**

A single phase IMRT plan (or equivalent if using 3D Conformal approach) should be produced for patients treated in Stage A1 and an inverse-planned IMRT treatment plan should be produced for patients treated in Stage B. Type B algorithms (e.g., collapsed cone, AAA) must be used for dose calculation.

## **Dose constraints**

**Stage A1**

**All constraints corrected using the linear quadratic equation and are valid only for 35Gy/15# and should not be corrected**

| **Structure** | **Naming convention*** | **DVH constraint** |
| --- | --- | --- |
| PTV | PTV | D99% > 95%  V95% > 99% |
| ICRU Maximum Dose | Please label patient outline as “External” | D1.8cc < 107% |
| Spinal Cord PRV | SpinalCord_PRV | None |
| Heart | Heart | Dmean < 23Gy  V28Gy < 45% |
| Combined lungs | Lungs | Dmean < 15Gy  V18Gy < 25% |
| Stomach | Stomach | None |
| Liver | Liver | Dmean < 26Gy  V28Gy<30% |
| Individual kidneys | Kidney_L and Kidney_R | V18Gy < 25% |

**Stage B:**

The following dose objectives (Warren et al., 2014) and nomenclature (Santanam et al., 2012) should be used:

| **Structure name** | **Constraint** | **Optimal** | **Mandatory** |
| --- | --- | --- | --- |
| PTV | V95% (47.5Gy)  Dmedian | > 95%  100% | ≥ 90%  The median should be between 98-102% of the prescription dose. |
| External | D1.8cc |  | <107% of highest prescribed dose |
| SpinalCord_PRV | D0.1cc | < 40Gy | < 42 Gy |
| Heart | Dmean  V30Gy | < 25Gy  < 45% | <30Gy  - |
| Lungs  (Combined lungs) | Dmean  V20Gy | < 17Gy  < 20% | <19Gy  ≤25% |
| Stomach_excl_PTV  (Stomach excluding PTV) | V50Gy | < 16cc | < 25cc |
| Liver | Dmean  V30Gy | ≤28Gy  < 30% | ≤30Gy  - |
| Kidney_L and Kidney_R  (Individual kidneys) | V20Gy | < 25% | ≤30% |

## **Treatment delivery and verification**

The treatment should be delivered in a single IMRT (or 3D Conformal approach in Stage A1 only) given the target volume described and the normal tissue constraints above, it is up to the individual participating centre to decide the field arrangements. It is recommended that the best available positional verification methods should be used to ensure correct delivery.

**Stage A1**

The use of cone beam CT matched to planning CT scans is recommended. The minimum on-treatment verification is for imaging the initial three fractions so that a correction for systematic error can be applied and then continue with weekly imaging. The isocentre should be moved if disagreement is seen in excess of agreed tolerance levels based on local study – typically 5mm.

**Stage B**

**The use of cone beam CT matched to planning CT scans is mandated**.

The minimum protocol for verification is on-line imaging of the initial five fractions so that a correction for systematic error can be applied and then continue with minimum weekly imaging thereafter for Stage B patients. The isocentre should be moved if disagreement is seen in excess of agreed tolerance levels based on local study – typically 5mm.

## **Management of radiotherapy toxicity**

Radiotherapy interruptions will usually not be necessary. However, if radiation is held for any reason, all systemic therapy must also be held, including M6620 (Berzosertib). Interruptions may be kept to a minimum by the use of ancillary therapy and vigorous nutritional support. Interruptions are permitted only on the basis of toxicity. However, if the RT interruptions are due to either technical reasons or due to bank holidays, the schedule should be made up to account for this either on a Saturday or at the end of the treatment.

| **Toxicity** | **Radiotherapy treatment interruption required:** | **Restart radiotherapy:** |
| --- | --- | --- |
| Oesophagitis | Grade 4; see management of symptoms below. Aim to limit interruption to 3 treatment days (fractions). |  |
| **Haematological toxicity** | |  |
| Neutrophils | Stop RT treatment if neutrophils <0.5 x 10^9^/L or platelet count <25 x 10^9^/L and monitor every 48 hours.  Note: systemic therapy should be interrupted initially as per local policy. | If levels recover to 0.5 x 10^9^/L (neutrophils) and 25 x 10^9^/L (platelets) |
| Platelets |  |  |
| **Non-Haematological toxicity** | |  |
| **All toxicities** | Grade 3: stop systemic treatment first and aggressive management should be pursued as per protocol, if no recovery to ≤grade 2 within 48h, consider withholding radiotherapy as follows: | |
| Vomiting | ≥6 episodes of vomiting (≥ grade 3) lasting ≥ 3 days and unresponsive to antiemetics | Resolves to ≤ grade 2 |
| Diarrhoea | An increase from a patients usual bowel habit of ≥ 7 watery stools/day (≥ grade 3) and unresponsive to antidiarrhoeals | Resolves to ≤ grade 2 |
| Weight loss | Loss ≥ 10% (≥ grade 2) of pre-treatment weight |  |
| Other^1^ | Grade 3: withhold radiotherapy and chemotherapy | Resolves to ≤ grade 2 |

^1^Rarely, non-treatment related or unexpected toxicities may require interruption of therapy at the discretion of the treating oncologist. Interruption of therapy may continue until the toxicity has regressed to ≤grade 2 to allow resumption of therapy; however, every effort should be made to limit treatment interruptions to 1-2 weeks

**Oesophagitis**

If Grade 4 oesophagitis occurs, and a treatment interruption is being considered, every effort should be made to limit it to 3 treatment days or less. Patients requiring hospitalization because of oesophagitis may have their treatment interrupted. Acute oesophageal toxicity, which typically can occur within 2 weeks of the initiation of treatment and manifests as dysphagia, odynophagia, reflux symptoms, etc. should be pharmacologically managed with the following approach and should be initiated at the first signs or symptoms of oesophageal toxicity. Double PPI dose or start PPI, soluble paracetamol, oramorph, fluconazole and follow local policy. If a patient develops grade 3 oesophagitis in the last week of treatment (i.e., with 5 or fewer radiation treatments remaining), radiation therapy (but not chemotherapy) may continue at the discretion of the treating physician.

If interruption of therapy (< 2 weeks) becomes necessary, radiation therapy should be completed to the prescribed doses. If treatment restarts and there are week-ends left aim to compensate for days lost with treatment Saturdays. No BID fractionation is permitted.

Total number of fractions and elapsed days should be carefully reported. If an interruption of more than 2 weeks is necessary, resumption of treatment is at the discretion of CI. The patient’s treatment plan will be considered a major deviation, but follow-up will be continued. Any toxicity that requires a dose reduction must be documented in the patient notes.

## **The management of unscheduled gaps in radiotherapy treatment**

In the event of unscheduled gaps to radiotherapy treatment, these should be managed as described above (section 12.8)

CHARIOT patients should be managed as Category 1 patients in Stage B and Category 3 in stage A.

Where possible during concurrent chemoradiotherapy phase patients should continue with RT if unable to tolerate chemotherapy in Stage B. Patients should be withdrawn from trial if RT is delayed by greater than 2 weeks.

## **Radiotherapy quality assurance**

In the first instance, any queries regarding radiotherapy quality assurance for CHARIOT should be addressed to the national Radiotherapy Trials QA group (RTTQA) contact ([CHARIOT.RTTQA@wales.nhs.uk](mailto:CHARIOT.RTTQA@wales.nhs.uk) or [www.rttrialsqa.org.uk](http://www.rttrialsqa.org.uk)).

Centres accredited for SCOPE2 can take part in the trial with no further pre-trial QA required, as these centres will have already completed an outlining and planning exercise, and submitted a process document for review by RTTQA.

Real time review of all patients will not be required instead there will be timely retrospective review of radiotherapy plans in the event of any unexpected or severe toxicity. All images, outlines, plan and dose data (DICOM) should be submitted to the RTTQA contact who will co-ordinate review of the data to check protocol compliance.

# Evaluation of response

## Tumour assessment

A clinical and radiological evaluation of malignancy, as judged appropriate by the Investigator, and in line with the protocol, must be performed before starting the study treatment where applicable. The same methods that detect lesions at baseline will be used to follow these lesions throughout the study. To ensure compatibility, the radiological assessments used to assess response must be performed using identical techniques. Imaging based evaluation is preferred to evaluation by clinical examination when both methods have been used to assess the anti-tumour effect of a treatment.

**Baseline evaluations**

These will include radiological measurements of the extent of disease by CT scan or PET-CT scans and endoscopy will also be performed. All areas of disease present must be mentioned (even if specific lesions are not going to be followed for response) and the measurements of all measurable lesions must be recorded on the scan reports. Any non-measurable lesions must be stated as being present.

**Evaluations during treatment and at off-study**

Tumour assessment will be repeated as per the schedule of events given or more frequently if clinically indicated. All lesions measured at baseline must be measured at subsequent disease assessments, and recorded on the scan reports. All non-measurable lesions noted at baseline must be reported as present or absent. Investigators must ensure that their radiologists are aware of the requirement to follow up and measure every target lesion mentioned at baseline and comment on the non-target lesions in accordance with RECIST (V1.1) criteria.

## Tumour response

To be assigned a status of CR or PR, changes in tumour measurements must be confirmed by two consecutive observations. To be assigned a status of stable disease (SD), follow-up measurements must have met the SD criteria at least once and at least six weeks after study treatment is started. Should rapid tumour progression occur before the completion of treatment the patient will be classified as having early progression (EP). Tumour response should be classified as “not evaluable” (NE), only when it is not possible to classify it under another response category, e.g., when baseline and/or follow-up assessment is not performed or not performed appropriately. The applicable overall response category for each visit that includes disease assessment must be recorded in the medical record for inclusion in the appropriate CRF in OpenClinica.

## Other definitions of outcome:

**Toxic death:** Any death to which drug toxicity is thought to have a major contribution.

**Early death:** Death during the first three weeks of treatment that is not a toxic death.

# safety reporting

The Investigator will monitor each patient for clinical and laboratory evidence of adverse events on a routine basis throughout the study. Should an Investigator become aware of any study drug related SAEs following this period these must also be reported as stated below. Adverse event monitoring starts from the time the patient consents to the study until they complete the trial. All reportable AEs will be followed to a satisfactory conclusion. Any reportable drug-related AEs that are unresolved at the end of treatment visit are to be followed up by the Investigator until resolution or stabilisation. All AEs reported to the Trial Office will be processed according to internal SOPs. The Trial Office may request additional information for any AE as judged necessary.

## Adverse Event Definitions

An Adverse Event or experience (AE) is any untoward medical occurrence in a study subject temporally associated with the administration of an investigational medicinal product (IMP) or a comparator product, whether or not considered related to the IMP or a comparator product. An AE can therefore be any unfavourable and unintended sign, symptom, disease (new or exacerbated) and /or significant abnormal laboratory or physiological observation temporally associated with the use of a medicinal product. For marketed medicinal products, this also includes failure to produce expected benefits (i.e. lack of efficacy), abuse or misuse.

**A Serious Adverse Event (SAE)** is any AE, regardless of dose, causality or expectedness, that:

| - **Results in death** |  |
| --- | --- |
| - **Is life-threatening** | This refers to an event in which the subject was at risk of death at the time of the event. It does not refer to an event, which hypothetically might have caused death, if it were more severe. |
| - **Requires in-patient hospitalisation  or prolongs existing inpatient hospitalisation** | In general, hospitalisation signifies that the subject has been admitted (usually involving at least an overnight stay) at the hospital or emergency ward for observation and/or treatment that would not have been appropriate in the physician’s office or out-patient setting. Complications that occur during hospitalisation are AEs. If a complication prolongs hospitalisation or fulfils any other serious criteria, the event is serious. When in doubt as to whether hospitalisation occurred or was necessary, the AE should be considered serious. |
| - **Results in persistent or significant incapacity or disability** | This means a substantial disruption of a person’s ability to conduct normal life functions. It does not include experiences of relatively minor medical significance or accidental trauma (e.g. sprained ankle), which do not constitute a substantial disruption. |
| - **Is a congenital anomaly or birth defect** |  |
| - **Is any other medically important event** | Defined as an event that may jeopardise the patient or may require intervention to prevent one of the outcomes listed above. Any new primary cancer must be reported as an SAE. |

**An Adverse Drug Reaction (ADR)** is an AE which is considered to be causally related to any dose of the IMP. This means that a causal relationship between the IMP and the AE is at least a reasonable possibility, i.e., the relationship cannot be ruled out.

**An Unexpected Drug Reaction** is an adverse drug reaction, the nature or severity of which, is not consistent with applicable product information (referring to information in SPC or IB).

**A Suspected Unexpected Serious Adverse Drug Reaction (SUSAR)** is a serious adverse drug reaction, the nature or severity of which is not consistent with the applicable product information (e.g. Investigator’s Brochure for an unapproved investigational product or SPC for an approved product).

## Clinical laboratory abnormalities and other abnormal assessments as AEs and SAEs

Abnormal laboratory findings (e.g., clinical chemistry, haematology, urinalysis) or other abnormal assessments (e.g., ECGs, X-rays and scans) that are judged by the Investigator as clinically significant will be recorded as AEs or SAEs if they meet the definitions given above.

Clinically significant abnormal laboratory findings or other abnormal assessments that are detected during the study or are present at baseline and significantly worsen following the start of the study will be reported as AEs or SAEs. However, clinically significant abnormal laboratory findings or other abnormal assessments that are associated with the disease being studied, unless judged by the Investigator as more severe than expected for the patient’s condition, or that are present or detected at the start of the study and do not worsen, will not be reported as AEs or SAEs. The Investigator will exercise his or her medical and scientific judgment in deciding whether an abnormal laboratory finding or other abnormal assessment is clinically significant.

## Determining adverse event causality

The assessment of “relatedness” must be determined by a medically qualified individual and is the responsibility of the PI at site or agreed designee. AEs that will be considered related will include any AE that is documented as possibly, probably or definitely related to protocol treatment. The assessment of relatedness is made using the following:

| **Classification** | **Relationship** | **Definition** |
| --- | --- | --- |
| **drug-related** | **Definitely related** | - Starts within a time related to the study drug administration *and* - No obvious alternative medical explanation. |
|  | **Probably related** | - Starts within a time related to the study drug administration *and* - Cannot be reasonably explained by known characteristics of the patient’s clinical state. |
|  | **Possibly related** | - Starts within a time related to the study drug administration *and* - A causal relationship between the study drug and the adverse event is at least a reasonable possibility. |
| **not drug related** | **Probably not related** | - The time association or the patient’s clinical state is such that the study drug is not likely to have had an association with the observed effect. |
|  | **Definitely not related** | - The AE is definitely not associated with the study drug administered. |

The Investigator must endeavour to obtain sufficient information to confirm the causality of the adverse event (i.e. relation to surgery, study drug, background treatment, other illness, progressive malignancy etc) and give their opinion of the causal relationship between each AE and study drug. This may require instituting supplementary investigations of significant AEs based on their clinical judgement of the likely causative factors and/or include seeking a further specialist opinion.

## Reference safety information (RSI) for assessment of expectedness

- The reference safety information (RSI) for the IMP M6620 (Berzosertib) is section 7 of the IB for M6620 (Berzosertib) and lists all the expected side effects associated with the use of M6620 (Berzosertib).
- The Reference Safety Information for the IMP capecitabine is the SmPC version provided by OCTO (approved for use in this trial by the MHRA).^1^
- The Reference Safety Information for the IMP cisplatin is the SmPC version provided by OCTO (approved for use in this trial by the MHRA).^1^

^1^It is not specified that any particular brand of Capecitabine or Cisplatin must be prescribed, however irrespective of the brand prescribed, the RSI to be referenced is provided by OCTO. N.B: This may not be the latest SmPC version available online.

A copy of the current approved version of the RSI documents for each IMP must be held in the Site File for reference. Any change or update to the RSI during the trial will be made via substantial amendment.

Please note that the list of expected side effects in the SmPCs for cisplatin and capecitabine are those listed for patients receiving standard chemoradiotherapy or chemotherapy alone. It is therefore possible that in this study population, where combination of M6620 (Berzosertib) is used with standard chemoradiotherapy or chemotherapy alone, other side effects may occur, or the patient might suffer a more severe reaction.

## Expected adverse events associated with Radiotherapy

Fatigue, oesophagitis, odynophagia, dyspnoea, nausea, vomiting, skin erythema and desquamation. Late toxicity risks of lung fibrosis, oesophageal stricture, oesophageal perforation, cardiac toxicity and secondary malignancy.

## Summary of trial safety reporting requirements

Please note all standard treatment toxicities **must be** reported because the TiTE-CRM trial design is based on an expected frequency of standard treatment toxicities plus M6620 (Berzosertib) related toxicities.

| **Event** | **DLT** | **SAE^1^** | **AE/SAE** | **AE/SAE** |
| --- | --- | --- | --- | --- |
| **AE/SAE defined as Dose limiting toxicity (DLT)** defined as per NCI CTCAE v4.03 | **Email reporting form within 24 hours** | | **Report in AE CRF** | **Non reportable** |
| Absolute neutrophil count (ANC) <0.5 x 10^9^/L for >7days | **X** | **X** | **X** |  |
| Febrile Neutropenia (ANC <1.0 x 10^9^/L, fever >38.5^o^C) for >3 days | **X** | **X** | **X** |  |
| Infection (documented clinically or microbiologically) with Grade 3 or 4 neutropenia (absolute neutrophil count <1.0 x 10^9^/L) | **X** | **X** | **X** |  |
| Platelets <25 x 10^9^/L (Grade ≥4) | **X** | **X** | **X** |  |
| Clinically significant bleeding attributed to grade 3 thrombocytopenia or requiring platelet transfusion | **X** | **X** | **X** |  |
| Grade ≥3 oesophagitis onset during induction chemotherapy or within 2 weeks of starting radiotherapy | **X** | **X** | **X** |  |
| Grade ≥3 pneumonitis onset within 3 months of starting radiotherapy | **X** | **X** | **X** |  |
| Grade ≥3 elevation of ALT or AST lasting 8 days or more | **X** | **X** | **X** |  |
| A concurrent elevation of ALT or AST >3 × ULN and total bilirubin >2 × ULN in whom there is no evidence of biliary obstruction or other causes that can reasonably explain the concurrent elevation | **X** | **X** | **X** |  |
| Grade ≥3 nausea or vomiting not controlled by optimal outpatient anti-emetic treatment | **X** | **X** | **X** |  |
| Grade ≥3 diarrhoea despite optimal outpatient anti-diarrhoeal medication use | **X** | **X** | **X** |  |
| Other grade ≥ 3 effects thought to be directly treatment related to the combination of M6620 (Berzosertib) with radiotherapy or chemotherapy | **X** | **X** | **X** |  |
| Any toxicity causing a delay of radiotherapy completion by greater than one week for Stage A | **X** | **X** | **X** |  |
| Any toxicity causing radiotherapy delivery delay by ≥3 consecutive fractions for stage B | **X** | **X** | **X** |  |
| Missing 2 consecutive doses of M6620 (Berzosertib) within a cycle due to Grade ≥3 toxicity | **X** | **X** | **X** |  |
| Death due to drug related complications | **X** | **X** | **X** |  |
| Cardiac toxicity: see section 9.1.2 | **X** | **X** | **X** |  |
| A delay of any of the 3 treatments of 7 days or more within a cycle due to treatment related toxicity | **x** | **x** | **x** |  |
| **AE/SAE defined as late DLT** (onset 6 weeks after completion of radiotherapy) as per RTOG/EORTC late toxicity and Mellow Dysphagia score (Appendix C, D) |  |  |  |  |
| Grade ≥3 lung and heart toxicity – onset 6 weeks after completion of radiotherapy | **X** | **X** | **X** |  |
| Grade 3 oesophageal toxicity with an increase in Mellow score by ≥2 since baseline (need to exclude disease progression on CT and/or endoscopy and biopsy) - onset 6 weeks after completion of radiotherapy | **X** | **X** | **X** |  |
| Grade 4 oesophageal toxicity - onset 6 weeks after completion of radiotherapy | **X** | **X** | **X** |  |
| **Medically important events in the context of this trial (considered dose limiting and possibly related to M6620 (Berzosertib) combined with Radiotherapy +/- chemotherapy by the TMG)** | **Email reporting form within 24 hours** | | **Report in AE CRF** | **Non reportable** |
| Any AE not listed above that is grade ≥3 |  | **X** | **X** |  |
| AE considered more severe than expected |  | **X** | **X** |  |
| AE Grade <3 that is **unexpected** and thought to be directly treatment related to the combination of M6620 (Berzosertib) with radiotherapy +/- chemotherapy |  | **X** | **X** |  |
| AE resulting in withdrawal |  | **X** | **X** |  |
| Any late grade ≥3 toxicities – onset 6 weeks after completion of radiotherapy |  | **X** | **X** |  |
| Any late grade 2 toxicities – onset 6 weeks after completion of radiotherapy |  |  | **X** |  |
| Acute Hypersensitivity reaction |  | **X** | **X** |  |
| **Expected non-dose limiting toxicities** | **Email reporting form within 24 hours** | | **Report in AE CRF** | **Non reportable** |
| Anaemia |  | **If grade≥4** | **X** |  |
| Abdominal pain |  | **If grade≥4** | **X** |  |
| Cough |  |  | **X** |  |
| Decreased appetite |  | **If grade≥4** | **X** |  |
| Elevated ALT/AST lasting less than 8 days |  | **If grade≥4** | **X** |  |
| Fatigue |  | **If grade≥4** | **X** |  |
| Headache |  |  | **X** |  |
| Low lymphocytes |  |  | **X** |  |
| Infusion-related site reaction |  | **If grade≥3** | **X** |  |
| Skin rash/discolouration related to sun exposure |  | **If grade≥4** | **X** |  |
| Grade <3 nausea or vomiting |  |  | **X** |  |
| **All other AEs, abnormal assessments or laboratory results** | **Email reporting form within 24 hours** | | **Report in AE CRF** | **Non reportable** |
| All other AEs, assessments, abnormal laboratory results, if clinically significant |  | **If grade≥3** | **X** |  |
| AE is life-threatening |  | **X** | **X** |  |
| AE requires in-patient hospitalisation or prolongs existing inpatient hospitalisation |  | **X** | **X** |  |
| AE results in persistent or significant incapacity or disability |  | **X** | **X** |  |
| AE is a congenital anomaly or birth defect |  | **X** | **X** |  |
| AE is any other medically important event |  | **X** | **X** |  |
| **Disease progression and resultant death** | **Email reporting form within 24 hours** | | **Report in AE CRF** | **Non reportable** |
| Hospitalisation (for progression or procedures planned prior to informed consent ) |  |  |  | **X** |
| Clinical symptoms of progression |  |  |  | **X** |
| Death |  | **Possibly related directly to M6620 (Berzosertib) or the combination of M6620 (Berzosertib) + radiotherapy +/- chemotherapy** | **Report death and reason on death notification CRF** |  |

## Suspected Unexpected Serious Adverse Drug Reactions (SUSARs)

All SUSARS must be reported by the Trial Office to the responsible Authority and REC within the required timelines:

- Fatal or life threatening SUSARs will be reported within 7 days of the Trial Office receiving the initial report. Any additional information will be reported within eight days of sending the first report.
- All other SUSARs will be reported within 15 days of the Trial Office receiving the initial report

In addition, other safety issues qualify for expedited reporting where they might materially alter the current risk assessment of an IMP or be sufficient to change IMP administration or the overall conduct of the trial. Chemotherapy SUSARs will be reported via the Yellow Card Scheme.

## Expedited reporting of SAEs

The following SAE reporting requirements apply regardless of the Investigator’s assessment of the causality or expectedness of the SAE. All SAEs should be reported on the trial SAE report form (see SAE report form and completion guidelines).

If a Serious Adverse Event occurs that requires reporting, a Serious Adverse Event reporting form should be completed and **scanned and emailed as a PDF attachment** within 24 hours of becoming aware of the event to:

**Pharmacovigilance Office, OCTO**

**Email:** octo-safety@oncology.ox.ac.uk

**Tel no:** +44 (0) 01865 617082

If the SAE has not been reported within the specified timeframe, a reason for lateness must be provided when sending the SAE Report Form.

Investigators should also adhere to their local Trust policy for incident and SAE reporting in research. AEs which are serious must be reported to the Trial Office from the first dose of study medication up to and including 30 days after administration of the last dose of study treatment, or the end of the DLT reporting period, whichever is longer. Any SAE that occurs at any time after completion of treatment or after the designated follow-up period that the Investigator and/or Sub-Investigator consider to be related to any study drug must be reported to the Trial Office.

The Trial Office will be responsible for reporting all SAEs to MERCK KGAA, DARMSTADT GERMANY within 24 hours of receiving an SAE report.

## Follow-up of Serious Adverse Events

A follow-up report must be completed when the SAE resolves, is unlikely to change, or when additional information becomes available. If the SAE is a suspected SUSAR then follow up information must be provided as requested by the Trial Office.

If new or amended information on a reported SAE becomes available, the Investigator should report this on a new SAE form using the completion guidelines. If using the original form to notify further information, you must initial and date all new or amended information so that all changes are clearly identified.

SAEs that are considered to be definitely unrelated to the trial intervention will not be followed up and monitored.

## Reporting Adverse Events on the CRF

All AEs, including Serious AEs must be recorded on the case report forms (CRF) for that patient (unless otherwise specified in section 14.10). The information provided will include date of onset, event diagnosis (if known) or sign/symptom, severity, time course, duration and outcome and relationship of the AE to study drug. Any concomitant medications or any other therapy used to treat the event must be listed. The Investigator will provide an “other” cause for serious AEs considered to be unrelated to the study drug. Sites should ensure data entered into the CRF is consistent with the SAE report information where applicable.

Each separate AE episode must be recorded. For example, if an AE resolves completely or resolves to baseline and then recurs or worsens again, this must be recorded as a separate AE. For AEs to be considered intermittent, the events must be of similar nature and severity.

AEs occurring from the first dose of study medication up to the end of study visit must be recorded on the CRF. AEs may be spontaneously reported by the patient and/or in response to an open question from study personnel or revealed by observation, physical examination, or other diagnostic procedures. Any clinically relevant deterioration in laboratory assessments or other clinical finding is considered an AE. AEs which result in early withdrawal must be reported using the Early Withdrawal Form.

***Terms and Grading of Events***

All adverse events and toxicities must be graded according to the NCI Common Terminology Criteria for adverse events (NCI-CTCAE) Version 4.0 (currently up to Version 4.03).

## Events exempt from being reported as AE/ SAEs

### Progression of underlying disease

Disease progression and resultant death will be captured on the CRF.  Adverse events including hospitalisation that are clearly consistent with disease progression will not be reported as individual AE/SAEs. Clinical symptoms of progression will only be reported as adverse events if the symptom cannot be determined as exclusively due to the progression of the underlying malignancy, or does not fit the expected pattern of progression for the disease under study.

Every effort should be made to document the objective progression of underlying malignancy. In some cases, the determination of clinical progression may be based on symptomatic deterioration. For example, progression may be evident from clinical symptoms, but is not supported by tumour measurements. Or, the disease progression is so evident that the Investigator may elect not to perform further disease assessments.

### Death on study

Death on study is to be recorded on the Death CRF. The Investigator must clearly state in the source data if a causal relationship to the study IMP or other protocol treatment intervention is suspected and this should be the primary reason given on the Death CRF. If study treatment or other protocol intervention is suspected an SAE form should also be completed.

### Elective admissions and supportive care

Admissions to hospital for patient convenience or for procedures planned prior to informed consent or investigations or treatment as specified in this protocol and standard supportive care are not SAEs, and do not require SAE reporting.

## Informing Investigators of new safety information

The Trial Office or the Chief Investigator will ensure that all investigators are kept informed in a timely manner, as new safety profile information becomes available. Investigators are responsible for briefing their study team and onward transmission to their R&D office as appropriate.

# pregnancy

Expedited reporting is required for pregnancies (in a participant or partner) occurring within 6 months of a participants last dose of the study drug or 13 weeks after last patient treatment, whichever comes sooner. A Pregnancy Form should be completed and scanned and emailed to the Trial Office within the same timelines as an SAE. All reported pregnancies should be followed and the outcome reported using the same form. If the outcome of the pregnancy meets any of the criteria for seriousness, it must also be reported as an SAE.  Examples of pregnancy outcomes that are SAEs include reports of:

- congenital anomalies or developmental delay, in the foetus or the child.
- foetal death and spontaneous abortion.
- suspected adverse reactions in the neonate that are classified as serious.

Women who become pregnant should be withdrawn from trial treatment immediately.

# Defining the end of trial

For Stage A1 the last patient last visit (LPLV) will be 9 weeks post end of radiotherapy for Stage A2 LPLV will be 8 weeks post end of chemotherapy, and for Stage B the LPLV will be 13 weeks post end of radiotherapy. However for Stage A1 & A2 this will be followed by the non-interventional phase of follow-up, which will continue for 12 months from start of treatment or when all participants have died (whichever comes first). This will be done via the hospital. But in the longer term this may be carried out via the Health & Social Care Information Centre. For the purpose of the Research Ethics Committee approval the trial end date will be the last patient start of treatment plus 24 weeks for Stage B. The Clinical Study Report (CSR) will be presented within 1 year of end of trial.

The sponsor and the Chief Investigator reserve the right to terminate the study earlier at any time. In terminating the study, they must ensure that adequate consideration is given to the protection of the participants’ best interests.

# Statistical considerations

## Sample size and power

Sample size estimates are based on 1,000 simulated TiTE-CRM trials using the same characteristics that the actual trial will be based upon. The patients will not be replaced and the TiTE-CRM will use all accumulated critical toxicity summary data. For Stage A1, to treat 10 patients at a particular treatment plan or reach a maximum of 20 patients, 18 (95% C.I.: (10, 20)) patients are required. For Stage A2, to treat 6 patients at dosing schedule 4 or reach a maximum of 20 patients, 16 (95% C.I.: (11, 20)) are required. Stage B is expected to recruit a minimum of 15 patients and has a maximum sample size of 25.

# Statistical analysis plan

For all analyses, patients will be included according to the treatment to which they are assigned. All patients, regardless of how much treatment received and follow-up completed, will contribute to analysis.

It is therefore important that every effort is made to encourage patients, including those patients who do not receive/complete their allocated treatment, to attend for follow-up clinic visits to avoid bias in the analysis of the results.

A detailed statistical analysis plan will be available from the time the first patient is recruited and will be finalised before any analysis is undertaken. The analysis plan will be written in accordance with the current OCTRU standard operating procedures and will be finalised and agreed by the trial statistician and the CI. Sites must report any unintended deviations/violations to OCTO according to the procedure outlined during site initiation training.

## Inclusion in analysis

All patients enrolled in the study, will be accounted for and included in the analyses. The number of patients who were not evaluable, who died or withdrew before treatment began will be recorded. The distribution of follow-up time will be described and the number of patients lost to follow-up will be given.

Variables will be analysed to determine whether the criteria for the study conduct are met. This will include a description of patients who did not meet all the eligibility criteria, an assessment of protocol deviations, study drug accountability and other data that impact on the general conduct of the study.

Baseline characteristics will be summarised for all enrolled patients. Patients who died or withdrew before treatment started or do not complete the required safety observations will be described and evaluated separately. Treatment related toxicity will be tabulated by type and grade of toxicity. Evaluable for toxicity: All patients will be evaluable for toxicity from the time of their first treatment. Adverse events will be summarised by the number of patients experiencing each type of event. The grades and causality will be reported.

## Subgroup analysis

No subgroup analysis is planned.

## Interim Analyses

The trial will use the TiTE-CRM to allocate dose and   critical toxicity data will be reviewed prior to deciding the treatment schedule for each patient recruited.

## Procedures for reporting any deviation(s) from the protocol

Any deviations from the original statistical plan will be described and justified in the final report.

## Analysis for Safety

The variables that define the DLTs and safety variables will be summarized by descriptive statistics with patients grouped according to schedule received. Number (with percentages) of patient with and without DLT will also be presented according to schedule.

## Final analysis

Based upon projected accrual rates, this trial (Stage A1, A2 and B) is expected to complete recruitment within 30 months of opening to recruitment. Final analysis for Stage A will be after all patients have been followed up for at least 3 months in Stage A1 and 26 weeks in Stage A2 while for Stage B, it will be performed within 12 months after Stage B last patient start of treatment.

# Trial committees

## Trial Management Group (TMG)

The Chief Investigator will chair a TMG responsible for overseeing the successful conduct and publication of the trial. The TMG will include Chief Investigator, Co‐ Investigators, Radiotherapy Team Representative, Trial Manager, Trial Statistician and others as required. The TMG will meet as necessary to discuss toxicity data and to decide on dose escalation. TMG membership and decision making procedures will be documented in the TMG charter.

## Safety Review Committee

There is no independent Data and Safety Monitoring Committee (DSMC) for this study. The Safety Review Committee (SRC) will be convened as required to review DLTs, review decisions as to the recommended dose to be administered, and review the stop/start rules for each stage. The main outcomes will be analysed as stated above in the analysis plan and will not be analysed as an interim analysis. SRC will have 3 independent members who will attend both open and closed session of the SRC meeting.

The SRC will consist of:

1. Trial Statistician

2. Independent Statistician

3. OCTO trial management representative

4. Either:

a. One Medical Oncologist and one Clinical Oncologist or

b. Two Clinical Oncologists

The SRC Charter document for this study will define the exact membership and who should be present for decisions to

be made. Further internal or external experts may be consulted by the SRC, as necessary. Any PI can request an ad hoc

SRC meeting at any time in order to facilitate the immediate communication of any emerging safety issues during

the course of the study.

## Trial Steering Committee

RIOC will act as the TSC. The role of RIOC is to provide oversight for the trial on behalf of the Sponsor and Funder. The TSC will provide overall supervision of the safe and effective conduct of the study. The TSC will review trial progress against agreed milestones, adherence to protocol, and patient safety, and consider new information. The TSC has the authority to recommend study closure where appropriate.

# Data management

## Database considerations

Data management will be performed via a web-based, bespoke trial database (OpenClinica). OpenClinica is a dedicated and validated clinical trials database designed for electronic data capture. See: <http://www.openclinica.org>.

The Trial Office will provide sites with instructions and a video link for training purposes.

The participants will be identified by a unique trial specific number and year of birth. Initials, age and gender will be stored as data items within the eCRF but will not be used to identify patient records. These details will be used to assess eligibility and for statistical review of participant demographics. The name and any other identifying detail will NOT be included in any trial data electronic file.

## Case reports forms (CRFs)

The Investigator and study site staff will ensure that data collected on each subject is recorded in the CRF as accurately and completely as possible. All appropriate laboratory data, summary reports and Investigator observations will be transcribed into the CRFs from the relevant source data held in the site medical record(s). CRFs entries will not contain any source data (unless otherwise specified in the completion instructions provided by the Trial Office). It is important to ensure that:

- the relevant CRFs are completed.
- all CRF data are verifiable in the source documentation or the discrepancies must be explained.
- CRF sections are completed in a timely fashion, as close to the visit or event being recorded as possible. This trial uses a continual reassessment model for dose escalation, where escalation decisions are made based on all critical toxicity summary data entered to date. It is necessary to enter all participant data as soon as possible to allow accurate assessment of the data and appropriate dose escalation decisions to be made.
- Data queries are resolved and documented by authorised study staff in a timely fashion. The reason for the change or correction should be given where appropriate.
- As much data as possible is entered and cleaned in preparation for each study database lock point.

**Note**: ‘in a timely fashion’ means within no more than 5 working days of the initial event and within 14 days of receipt of a data query unless otherwise specified.

**If a patient withdraws from the study the Trial Office must be informed within 24 hours using the Early Withdrawal Form, the reason must be noted and the patient must be followed-up as per protocol.**

## Accounting for missing, unused, or spurious data.

The statistical analysis plan will describe the procedure for accounting for missing, unused or spurious data.

## Clinical study report

All clinical data will be presented at the end of the study as data listings. These will be checked to confirm the lists accurately represents the data collected during the course of the study. The trial data will then be locked and a final data listing produced. The locked trial data may then be used for analysis and publication. The Clinical Study Report will be based on the final data listings. The Clinical Study Report will include the analysis of the routine survival data collected up to 12 months for Stage A1 and A2. The CSR will be presented within 1 year of end of trial.

# Study site management

## Study site responsibilities

The Principal Investigator (the PI or lead clinician for the study site) has overall responsibility for conduct of the study, but may delegate responsibility where appropriate to suitably experienced and trained members of the study site team. All members of the study site team must complete the Staff Contact and Responsibilities Sheet provided prior to undertaking any study duties. The PI must counter sign and date each entry in a timely manner, authorising staff to take on the delegated responsibilities.

## Study site protocol deviations

The Investigator must document and explain any deviations/violations from the approved protocol. The Investigator should promptly report any important deviations that might impact patient safety, data integrity or be a possible serious breach (see 22.7 below) to the Trial Office.

## Study site set up and activation

The PI leading the investigational study site is responsible for providing all required core documentation. Mandatory Site Training organised by the Trial Office must be completed before the site can be activated. The Trial Office will check to confirm that the site has all the required study information/documentation and is ready to recruit. The site will then be notified once they are activated on the trial database and able to enter patients.

## Arrangements for sites outside the UK

Not applicable

## Study documentation

The Trial Office will provide an Investigator Site File and Pharmacy File to each investigational site containing the documents needed to initiate and conduct the study. The Trial Office must review and approve any local changes made to any study documentation including patient information and consent forms prior to use. Additional documentation generated during the course of the trial, including relevant communications must be retained in the site files as necessary to reconstruct the conduct of the trial.

# Regulatory and ethical considerations

The Sponsor and Investigators will ensure that this protocol will be conducted in compliance with the UK Clinical Trials Regulations^^[[1]](#footnote-2)^^ and the applicable policies of the sponsoring organisation. Together, these implement the ethical principles of the Declaration of Helsinki (1996) and the regulatory requirements for clinical trials of an investigational medicinal product under the European Union Clinical Trials Directive.

## Ethical conduct of the trial and ethics approval

The Protocol, Patient Information Sheet, Consent Form and any other information that will be presented to potential trial patients (e.g. patient card or information that supports or supplements the informed consent) will be reviewed and approved by an appropriately constituted, independent Research Ethics Committee (REC). Principal Investigators will be approved by the REC.

## Regulatory Authority approval

This study will be conducted under a UK Medicines and Healthcare Products Regulatory Agency (MHRA) Clinical Trials Authorisation (CTA). Approval to conduct the study will be obtained from the Responsible Authority prior to initiating the study.

## NHS Research Governance

Investigators are responsible for ensuring they obtain local Trust management agreement to conduct the trial in accordance with local arrangements and policies.

## Protocol amendments

Amendments are changes made to the research following initial approval. A ‘substantial amendment’ is an amendment to the terms of the Responsible Authority application (if applicable), the REC application, or to the protocol or any other supporting documentation, that is likely to affect to a significant degree:

- the safety or physical or mental integrity of the subjects of the trial;
- the scientific value of the trial;
- the conduct or management of the trial; or
- the quality or safety of the investigational medicinal product(s) used in the trial.

Non-substantial amendments are those where the change(s) involve only minor logistical or administrative aspects of the study. All amendments will be generated and managed according to the Trial Office standard operating procedures to ensure compliance with applicable regulation and other requirements. Written confirmation of all applicable REC, regulatory and local approvals must be in place prior to implementation by Investigators. The only exceptions are for changes necessary to eliminate an immediate hazard to study patients (see below).

It is the Investigator’s responsibility to update patients (or their authorised representatives, if applicable) whenever new information (in nature or severity) becomes available that might affect the patient’s willingness to continue in the trial. The Investigator must ensure this is documented in the patient’s medical notes and the patient is re-consented if appropriate.

## Urgent safety measures

The sponsor or Investigator may take appropriate urgent safety measures to protect trial participants from any immediate hazard to their health or safety. Urgent safety measures may be taken without prior authorisation. The trial may continue with the urgent safety measures in place. **The Investigator must inform the Trial Office IMMEDIATELY if the study site initiates an urgent safety measure:**

The notification must include:

- Date of the urgent safety measure;
- Who took the decision; and
- Why the action was taken.

The Investigator will provide any other information that may be required to enable the Trial Office to report and manage the urgent safety measure in accordance with the current regulatory and ethical requirements for expedited reporting and close out. The Trial Office will follow written procedures to implement the changes accordingly.

## Temporary halt

The sponsor and Investigators reserve the right to place recruitment to this protocol on hold for short periods for administrative reasons **or** to declare a temporary halt. A temporary halt is defined a formal decision to:

- interrupt the treatment of subjects already in the trial for safety reasons;
- stop recruitment on safety grounds; or
- stop recruitment for any other reason(s) considered to meet the substantial amendment criteria, including possible impact on the feasibility of completing the trial in a timely manner.

The Trial Office will report the temporary halt via an expedited substantial amendment procedure. The trial may not restart after a temporary halt until a further substantial amendment to re-open is in place. If it is decided not to restart the trial this will be reported as an early termination.

## Serious Breaches

The Medicines for Human Use (Clinical Trials) Regulations require the Sponsor to notify any "serious breaches" to the MHRA within 7 days of the sponsor becoming aware of the breach. A serious breach is defined as “A breach of GCP or the trial protocol which is likely to effect to a significant degree:

- the safety or physical or mental integrity of the subjects of the trial; or
- the scientific value of the trial”

Investigators must notify the Trial Office within one working day if any serious breach of GCP is suspected. The Trial Office will review the event and, if appropriate a report will be submitted to the REC, Regulatory Authority and the NHS host organisation within 7 days of the Trial Office becoming aware of the breach as per Trials Office SOPs.

## Trial Reports

This protocol will comply with all current applicable Regulatory Authority, Research Ethics Committee and Sponsor reporting requirements.

The Trial Office will determine which reports need to be circulated to Principal Investigators and other interested parties. Study sites are responsible for forwarding trial reports they receive to their local Trust as required.

# Expenses and benefits

The participating study site may provide reasonable travel expenses incurred for attending additional research visits in excess of standard of care as per local practice. The local arrangements will be explained to the patient during the informed consent discussions prior to trial entry. However, there is no direct study funding to reimburse patient expenses.

# Quality assurance

## Risk assessment

A risk assessment and a monitoring plan will be prepared before the study opens and will be reviewed throughout the study if necessary in the light of significant changes while the study is ongoing or in response to outcomes from monitoring activities. Monitoring plans will be amended as appropriate.

## Monitoring

Regular monitoring will be performed according to the monitoring plan. Data will be evaluated for compliance with the protocol, completeness and accuracy. The Investigator and institutions involved in the study will permit study-related monitoring and provide direct on-site access to all study records and facilities if required. They will provide adequate time and space for the completion of monitoring activities.

Study sites will be monitored centrally by checking incoming data for compliance with the protocol, consistency, completeness and timing. The case report data will be validated using appropriate set criteria, range and verification checks. The study site must resolve all data queries in a timely manner. All queries relating to key outcome and safety data and any requiring further clarification will be referred back to the study site for resolution. For other non-critical data items, OCTO staff may resolve data queries centrally providing the correct answer is clear. Such changes will be clearly identified in the CRF and the study site informed.

Study sites will also be monitored remotely and/or by site visit as necessary to ensure their proper conduct of the trial. OCTO staff will be in regular contact with site personnel to check on progress and deal with any queries that they may have. Monitoring reports will be sent to the site in a timely fashion. The Investigator is expected to action any points highlighted through monitoring and must ensure that corrective and preventative measures are put into place as necessary to achieve satisfactory compliance.

If sites are required to provide copies of participant information to the Trial Office for remote monitoring purposes, all patient personal identifiers must be obliterated from the information.

## Audit and Regulatory Inspection

All aspects of the study conduct may be subject to internal or external quality assurance audit to ensure compliance with the protocol, GCP requirements and other applicable regulation or standards. It may also be subject to a regulatory inspection. Such audits or inspections may occur at any time during or after the completion of the study. Investigators and their host Institution(s) should understand that it is necessary to allow auditors/inspectors direct access to all relevant documents, study facilities and to allocate their time and the time of their staff to facilitate the audit or inspection visit. Anyone receiving notification of a Regulatory Inspection that will (or is likely to) involve this trial must inform the Trial Office without delay.

# Records retention & archiving

During the clinical trial and after trial closure the Investigator must maintain adequate and accurate records to enable the conduct of a clinical trial and the quality of the research data to be evaluated and verified. All essential documents must be stored in such a way that ensures that they are readily available, upon request for the minimum period required by national legislation or for longer if needed. The medical files of trial subjects must be retained in accordance with applicable national legislation and the host institution policy.

Retention and storage of laboratory records for clinical trial samples must also follow these guidelines.

Retention and storage of central laboratory records supporting PD endpoints and the disposition of samples donated via the trial must also comply with applicable legislation and Sponsor requirements.

It is the University of Oxford’s policy to store data for a minimum of 5 years. Investigators may not archive or destroy study essential documents or samples without written instruction from the Trial Office.

# Patient confidentiality

Personal data recorded on all documents will be regarded as confidential, and to preserve each patient’s anonymity, unique and anonymous trial specific number will be used for identification and year of birth will be recorded on the CRFs. Initials, age and gender will be stored as data items within the eCRF but will not be used to identify patient records. These details will be used to assess eligibility and for statistical review of participant demographics.

The Investigator site must maintain the patient’s anonymity in all communications and reports related to the research. The Investigator site team must keep a separate log of enrolled patients’ personal identification details as necessary to enable them to be tracked. These documents must be retained securely, in strict confidence. They form part of the Investigator Site File and are not to be released externally.

The study will comply with the General Data Protection Regulation (GDPR) and Data Protection Act 2018, which require data to be de-identified as soon as it is practical to do so. The processing of the personal data of participants will be minimised by making use of a unique participant study number only on all study documents and any electronic database(s) with the exception of the CRF, where participant initials and Year of Birth may be added.  All documents will be stored securely and only accessible by study staff and authorised personnel. The study staff will safeguard the privacy of participants’ personal data.

# Study funding

The CHARIOT trial is being funded in part by the New Agents Committee (NAC), on behalf of Cancer Research UK (CRUKD/15/011). The Oncology Clinical Trials Office is supported by Cancer Research UK core funding. Merck KGaA, Darmstadt Germany are providing a grant and free M6620 (Berzosertib) to support the study. This study is further supported via the University of Oxford core clinical and research infrastructure underpinned by strategic research programme grant funds. This trial is on the NIHR portfolio. Local research network support should be available at each site taking part to support entry of participants into this trial.

# Sponsorship and indemnity

## Sponsorship

The Sponsor will provide written confirmation of Sponsorship and authorise the trial commencement once satisfied that all arrangements and approvals for the proper conduct of the trial are in place. A separate study delegation agreement, setting out the responsibilities of the Chief Investigator and Sponsor will be put in place between the parties.

## Indemnity

The University has a specialist insurance policy in place which would operate in the event of any participant suffering harm as a result of their involvement in the research (Newline Underwriting Management Ltd, at Lloyd’s of London). NHS indemnity operates in respect of the clinical treatment that is provided.

## Contracts/Agreements

This trial is subject to the Sponsor’s policy requiring that written contracts/agreements are agreed formally by the participating bodies as appropriate. A Clinical Trial Agreement (CTA) will be placed between the Sponsor and participating organisations prior to site activation.

The Sponsor will also set up written agreements with any other external third parties involved in the conduct of the trial as appropriate.

# Publication policy

The sponsor will retain ownership of all data arising from the trial. The intention is to publish this research in a specialist peer reviewed scientific journal on completion of the study. The results may also be presented at scientific meetings and/or used for a thesis. The Investigators will be involved in reviewing drafts of the manuscripts, abstracts, press releases and any other publications arising from the trial and retain final editorial control. Authors will acknowledge that the study was Sponsored by and performed with the support of the Sponsor and other funding bodies as appropriate.

# References

1. Lewis KA, Lilly KK, Reynolds EA, Sullivan WP, Kaufmann SH, Cliby WA. Ataxia telangiectasia and rad3-related kinase contributes to cell cycle arrest and survival after cisplatin but not oxaliplatin. Molecular cancer therapeutics. 2009 Apr;8(4):855-63. PubMed PMID: 19372558. Pubmed Central PMCID: 2690640.

2. Cliby WA, Roberts CJ, Cimprich KA, Stringer CM, Lamb JR, Schreiber SL, et al. Overexpression of a kinase-inactive ATR protein causes sensitivity to DNA-damaging agents and defects in cell cycle checkpoints. The EMBO journal. 1998 Jan 2;17(1):159-69. PubMed PMID: 9427750. Pubmed Central PMCID: 1170367.

3. Ding L, Getz G, Wheeler DA, Mardis ER, McLellan MD, Cibulskis K, et al. Somatic mutations affect key pathways in lung adenocarcinoma. Nature. 2008 Oct 23;455(7216):1069-75. PubMed PMID: 18948947. Pubmed Central PMCID: 2694412.

4. Greenman C, Stephens P, Smith R, Dalgliesh GL, Hunter C, Bignell G, et al. Patterns of somatic mutation in human cancer genomes. Nature. 2007 Mar 8;446(7132):153-8. PubMed PMID: 17344846. Pubmed Central PMCID: 2712719.

5. Abedi-Ardekani B, Kamangar F, Sotoudeh M, Villar S, Islami F, Aghcheli K, et al. Extremely high Tp53 mutation load in esophageal squamous cell carcinoma in Golestan Province, Iran. PloS one. 2011;6(12):e29488. PubMed PMID: 22216294. Pubmed Central PMCID: 3246475.

6. Dulak AM, Stojanov P, Peng S, Lawrence MS, Fox C, Stewart C, et al. Exome and whole-genome sequencing of esophageal adenocarcinoma identifies recurrent driver events and mutational complexity. Nature genetics. 2013 May;45(5):478-86. PubMed PMID: 23525077. Pubmed Central PMCID: 3678719.

7. He H, Tian D, Guo J, Liu M, Chen Z, Hamdy FC, et al. DNA damage response in peritumoral regions of oesophageal cancer microenvironment. Carcinogenesis. 2013 Jan;34(1):139-45. PubMed PMID: 23027622.

8. Charrier JD, Durrant SJ, Golec JM, Kay DP, Knegtel RM, MacCormick S, et al. Discovery of potent and selective inhibitors of ataxia telangiectasia mutated and Rad3 related (ATR) protein kinase as potential anticancer agents. Journal of medicinal chemistry. 2011 Apr 14;54(7):2320-30. PubMed PMID: 21413798.

9. Reaper PM, Griffiths MR, Long JM, Charrier JD, Maccormick S, Charlton PA, et al. Selective killing of ATM- or p53-deficient cancer cells through inhibition of ATR. Nature chemical biology. 2011 Jul;7(7):428-30. PubMed PMID: 21490603.

10. Pires IM, Olcina MM, Anbalagan S, Pollard JR, Reaper PM, Charlton PA, et al. Targeting radiation-resistant hypoxic tumour cells through ATR inhibition. British journal of cancer. 2012 Jul 10;107(2):291-9. PubMed PMID: 22713662. Pubmed Central PMCID: 3394988.

11. Fokas E, Prevo R, Pollard JR, Reaper PM, Charlton PA, Cornelissen B, et al. Targeting ATR in vivo using the novel inhibitor VE-822 results in selective sensitization of pancreatic tumors to radiation. Cell death & disease. 2012;3:e441. PubMed PMID: 23222511. Pubmed Central PMCID: 3542617.

12. Crosby T, Hurt CN, Falk S, Gollins S, Mukherjee S, Staffurth J, et al. Chemoradiotherapy with or without cetuximab in patients with oesophageal cancer (SCOPE1): a multicentre, phase 2/3 randomised trial. The lancet oncology. 2013 Jun;14(7):627-37. PubMed PMID: 23623280.

13. Conroy T, Galais MP, Raoul JL, Bouche O, Gourgou-Bourgade S, Douillard JY, et al. Definitive chemoradiotherapy with FOLFOX versus fluorouracil and cisplatin in patients with oesophageal cancer (PRODIGE5/ACCORD17): final results of a randomised, phase 2/3 trial. The lancet oncology. 2014 Mar;15(3):305-14. PubMed PMID: 24556041

14. Knyrim K, Wagner H, Bethge N, Keymling, Vakil N. (1993). A controlled trial of an expansile metal stent for palliation of esophageal obstruction due to inoperable cancer. The New England Journal of Medicine; 78:1302-1307. PMID:7692297

15. [Allum WH](http://www.ncbi.nlm.nih.gov/pubmed/?term=Allum%20WH%5BAuthor%5D&cauthor=true&cauthor_uid=21705456), [Blazeby JM](http://www.ncbi.nlm.nih.gov/pubmed/?term=Blazeby%20JM%5BAuthor%5D&cauthor=true&cauthor_uid=21705456), [Griffin SM](http://www.ncbi.nlm.nih.gov/pubmed/?term=Griffin%20SM%5BAuthor%5D&cauthor=true&cauthor_uid=21705456), [Cunningham D](http://www.ncbi.nlm.nih.gov/pubmed/?term=Cunningham%20D%5BAuthor%5D&cauthor=true&cauthor_uid=21705456), [Jankowski JA](http://www.ncbi.nlm.nih.gov/pubmed/?term=Jankowski%20JA%5BAuthor%5D&cauthor=true&cauthor_uid=21705456), [Wong R](http://www.ncbi.nlm.nih.gov/pubmed/?term=Wong%20R%5BAuthor%5D&cauthor=true&cauthor_uid=21705456); Guidelines for the management of oesophageal and gastric cancer. [Gut.](http://www.ncbi.nlm.nih.gov/pubmed/?term=allum+2011+oesophageal+cancer) 2011 Nov;60(11):1449-72.

# APPENDIX A: ECOG performance scale

| Activity Performance Description | Score |
| --- | --- |
|  |  |
| Fully active, able to carry out all on all pre-disease performance without restriction. | 0 |
|  |  |
| Restricted in physically strenuous activity but ambulatory and able to carry out work of a light or sedentary nature, e.g. light housework, office work. | 1 |
|  |  |
| Ambulatory and capable of all self-care, but unable to carry out any work activities. Up and about more than 50% of waking hours. | 2 |
|  |  |
| Capable of only limited self-care. Confined to bed or chair more than 50% of waking hours. | 3 |
|  |  |
| Completely disabled. Cannot carry out any self-care. Totally confined to bed or chair. | 4 |

# Appendix B: measurement of disease - recist criteria

RESPONSE EVALUATION CRITERIA IN SOLID TUMOURS

Objective tumour response and time of progression will be measured according to the RECIST (Response Evaluation Criteria In Solid Tumours) criteria (version 1.1).

Response criteria are essentially based on a set of measurable lesions identified at baseline as target lesions, and – together with other lesions that are denoted as non-target lesions – followed until disease progression.

The following paragraphs are a quick reference to the RECIST criteria (version 1.1). The complete criteria are included in the published RECIST document:

Eisenhauer, EA, Therasse, P, Bogaerts, J, et al. New response evaluation criteria in solid tumours: revised RECIST guideline (version 1.1). Eur J Cancer 2009;45:228-247

And also available at: <http://www.eortc.be/RECIST>

**B.1 Measurability of tumour lesions at baseline**

**B.1.1 Definitions**

- **Measurable disease** - the presence of at least one measurable lesion. If the measurable disease is restricted to a solitary lesion, its neoplastic nature should be confirmed by cytology/histology.
- **Measurable lesions** - *tumour lesions* that can be accurately measured in at least one dimension (longest diameter to be recorded) as ≥ 20 mm with chest x-ray, and as ≥ 10 mm with CT scan or clinical examination [using callipers]. Bone lesions are considered measurable only if assessed by CT scan and have an identifiable soft tissue component that meets these requirements (soft tissue component > 10 mm by CT scan). *Malignant lymph nodes* must be ≥ 15 mm in the short axis to be considered measurable; only the short axis will be measured and followed. All tumour measurements must be recorded in millimetres (or decimal fractions of centimetres) by use of a ruler or callipers. Tumour lesions situated in a previously irradiated area, or in an area subjected to other loco-regional therapy, are usually not considered measurable unless there has been demonstrated progression in the lesion.
- **Non-measurable lesions** - All other lesions (or sites of disease), including small lesions are considered non-measurable disease. Bone lesions without a measurable soft tissue component, leptomeningeal disease, ascites, pleural/pericardial effusions, lymphangitis cutis/pulmonis, inflammatory breast disease, lymphangitic involvement of lung or skin and abdominal masses followed by clinical examination are all non-measurable. Nodes that have a short axis <10 mm at baseline are considered non-pathological and should not be recorded or followed.
- **Target Lesions.** When more than one measurable tumour lesion or malignant lymph node is present at baseline all lesions up to *a maximum of 5* *lesions total* (and a maximum of *2 lesions per organ*) representative of all involved organs should be identified as target lesions and will be recorded and measured at baseline. Target lesions should be selected on the basis of their size (lesions with the longest diameter), be representative of all involved organs, but in addition should be those that lend themselves to *reproducible repeated measurements*. Note that pathological nodes must meet the criterion of a short axis of ≥ 15 mm by CT scan and only the *short* axis of these nodes will contribute to the baseline sum. At baseline, the sum of the target lesions (longest diameter of tumour lesions plus short axis of lymph nodes: overall maximum of 5) is to be calculated and recorded.
- **Non-target Lesions**. All non-measurable lesions (or sites of disease) including pathological nodes (those with short axis ≥ 10 mm but < 15 mm), plus any measurable lesions over and above those listed as target lesions are considered *non-target lesions*. Measurements are not required but these lesions should be noted at baseline and should be followed as “present” or “absent”.

All baseline evaluations should be performed as closely as possible to the beginning of treatment and never more than 4 weeks before the beginning of the treatment.

**B.1.2 Methods of measurements**

The same method of assessment and the same technique should be used to characterize each identified and reported lesion at baseline and during follow-up. Assessments should be identified on a calendar schedule and should not be affected by delays in therapy, which may be treatment arm dependent. While on study, all target lesions recorded at baseline should have their actual measurements recorded on the CRF at each subsequent evaluation, even when very small (e.g. 2 mm). If it is the opinion of the radiologist that the lesion has likely disappeared, the measurement should be recorded as 0 mm. If the lesion is believed to be present and is faintly seen but too small to measure, a default value of 5 mm should be assigned. For lesions which fragment/split add together the longest diameters of the fragmented portions; for lesions which coalesce, measure the maximal longest diameter for the “merged lesion”.

- **Clinical Lesions**. Clinical lesions will only be considered measurable when they are superficial and ≥ 10 mm as assessed using callipers (e.g. skin nodules). For the case of skin lesions, documentation by colour photography including a ruler to estimate the size of the lesion is recommended. If feasible, imaging is preferred.
- **Chest X-ray.** Chest CT is preferred over chest X-ray, particularly when progression is an important endpoint, since CT is more sensitive than X-ray, particularly in identifying new lesions. However, lesions > 20 mm on chest X-ray may be considered measurable if they are clearly defined and surrounded by aerated lung.
- **CT, MRI.** CT is the best currently available and reproducible method to measure lesions selected for response assessment. This guideline has defined measurability of lesions on CT scan based on the assumption that CT slice thickness is 5 mm or less. When CT scans have slice thickness greater than 5 mm, the minimum size for a measurable lesion should be twice the slice thickness. MRI is also acceptable in certain situations (e.g. for body scans). While PET scans are not considered adequate to measure lesions, PET-CT scans may be used providing that the measures are obtained from the CT scan and the CT scan is of identical diagnostic quality to a diagnostic CT (with IV and oral contrast).
- **Ultrasound.** Ultrasound is not useful in assessment of lesion size and should not be used as a method of measurement. If new lesions are identified by ultrasound in the course of the study, confirmation by CT should be obtained.
- **Endoscopy, Laparoscopy**. The utilization of these techniques for objective tumour evaluation is not advised. However, they can be useful to confirm complete pathological response when biopsies are obtained or to determine relapse in trials where recurrence following complete response or surgical resection is an endpoint.
- **Tumour Markers**. Tumour markers alone cannot be used to assess objective tumour response. If markers are initially above the upper normal limit, however, they must normalize for a patient to be considered in complete response.
- **Cytology, Histology**. These techniques can be used to differentiate between PR and CR in rare cases if required by protocol (for example, residual lesions in tumour types such as germ cell tumours, where known residual benign tumours can remain). When effusions are known to be a potential adverse effect of treatment (e.g. with certain taxane compounds or angiogenesis inhibitors), the cytological confirmation of the neoplastic origin of any effusion that appears or worsens during treatment when the measurable tumour has met criteria for response or stable disease is advised to differentiate between response or stable disease and progressive disease.

**B.2 Tumour response evaluation**

All patients will have their BEST RESPONSE from the start of study treatment until the end of treatment classified as outlined below:

Complete or partial responses may be claimed only if the criteria for each are met at a subsequent time point at least 4 weeks later. Refer to the table B1and table B2 below.

Complete Response (CR): disappearance of all *target* and *non-target* lesions and normalization of tumour markers. Pathological lymph nodes must have short axis measures < 10 mm (Note: continue to record the measurement even if < 10 mm and considered CR). Tumour markers must have normalized. Residual lesions (other than nodes < 10 mm) thought to be non-malignant should be further investigated (by cytology or PET scans) before CR can be accepted.

Partial Response (PR): at least a 30% decrease in the sum of measures (longest diameter for tumour lesions and short axis measure for nodes) of target lesions, taking as reference the baseline sum of diameters. Non target lesions must be non-PD.

Stable Disease (SD): Neither sufficient shrinkage to qualify for PR nor sufficient increase to qualify for PD taking as reference the smallest sum of diameters on study.

Progressive Disease (PD): at least a 20% increase in the sum of diameters of measured lesions taking as references the smallest sum of diameters recorded on study (including baseline) AND an absolute increase of ≥ 5 mm. Appearance of new lesions will also constitute PD (including lesions in previously unassessed areas). In exceptional circumstances, unequivocal progression of non-target disease may be accepted as evidence of disease progression, where the overall tumour burden has increased sufficiently to merit discontinuation of treatment, for example where the tumour burden appears to have increased by at least 73% in volume (which is the increase in volume when all dimensions of a single lesion increase by 20%). Modest increases in the size of one or more non-target lesions are NOT considered unequivocal progression. If the evidence of PD is equivocal (target or non-target), treatment may continue until the next assessment, but on further documentation, the earlier date must be used.

**Table B1: Integration of target, non-target and new lesions into response assessment**

| **Target Lesions** | **Non-Target Lesions** | **New Lesions** | **Overall Response** | **Best Response for this category also requires** |
| --- | --- | --- | --- | --- |
| ***Patients with Target lesions ± non target lesions*** | | | | |
| CR | CR | No | CR | Normalization of tumour markers  All tumour nodes < 10 mm  Documented at least once ≥ 4 weeks from baseline |
| CR | Non-CR/Non-PD | No | PR | Documented at least once ≥ 4 weeks from baseline |
| CR | Not all evaluated | No | PR |  |
| PR | Non-PD/ not all evaluated | No | PR |  |
| SD | Non-PD/ not all evaluated | No | SD |  |
| Not all evaluated | Non-PD | No | NE |  |
| PD | Any | Any | PD |  |
| Any | PD | Any | PD |  |
| Any | Any | Yes | PD |  |
| ***Patients with Non target lesions ONLY*** | | | | |
| No Target | CR | No | CR | Normalization of tumour markers  All tumour nodes < 10 mm  Documented at least once ≥ 4 weeks from baseline |
| No Target | Non-CR/non-PD | No | Non-CR/ non-PD |  |
| No Target | Not all evaluated | No | NE |  |
| No Target | Unequivocal PD | Any | PD |  |
| No Target | Any | Yes | PD |  |
| Note: Patients with a global deterioration of health status requiring discontinuation of treatment without objective evidence of disease progression [or evidence of unequivocal disease progression] at that time should be reported as “*symptomatic deterioration”*. This is a reason for stopping therapy, but is NOT objective PD. Every effort should be made to document the objective progression even after discontinuation of treatment. | | | | |

Complete or partial responses may be claimed only if the criteria for each are met at a subsequent time point at least 4 weeks later. The best overall response can be interpreted from Table B2.

**Table B2: Response assessment after subsequent scan**

| **Response: First time point** | **Subsequent time point** | **BEST overall response** | **Also requires** |
| --- | --- | --- | --- |
| CR | CR | CR | Normalization of tumour markers  All tumour nodes < 10 mm |
| CR | PR | SD, PD or PR (see comment*) |  |
| CR | SD | SD provided minimum criteria for SD duration met, otherwise, PD |  |
| CR | PD | SD provided minimum criteria for SD duration met, otherwise, PD |  |
| CR | NE | SD provided minimum criteria for SD duration met, otherwise NE |  |
| PR | CR | PR |  |
| PR | PR | PR |  |
| PR | SD | SD |  |
| PR | PD | SD provided minimum criteria for SD duration met, otherwise, PD |  |
| PR | NE | SD provided minimum criteria for SD duration met, otherwise NE |  |
| NE | NE | NE |  |
| * may consider PR providing initial “CR” likely PR on subsequent review – then original CR should be corrected. Recurrence of lesion after true CR is PD. | | | |

**B.2.1 Frequency of tumour re-evaluation**

Participants will receive CT scans at baseline and at 24 weeks after the start of treatment as per standard treatment.

**B.2.2 Date of progression**

This is defined as the first day when the RECIST (version 1.1) criteria for PD are met.

**B.3 Reporting of tumour response**

All patients included in the study must be assessed for response to treatment, even if there is a major protocol treatment deviation or if they are ineligible, or not followed/re-evaluated. Each patient will be assigned one of the following categories: complete response, partial response, stable disease, progressive disease, early death from malignant disease, early death from toxicity, early death from other cause or unknown (not assessable, insufficient data).

Early death is defined as any death occurring before the first per protocol time point of tumour re-evaluation. The responsible investigator will decide if the cause of death is malignant disease, toxicity or other cause.

Patients for whom response is not confirmed will be classified as "unknown", unless they meet the criteria for stable disease (or the criteria for partial response in case of an unconfirmed complete response). Patients’ response will also be classified as "unknown" if insufficient data were collected to allow evaluation per these criteria.

# Appendix C: RTOG Late Toxicity score

Please refer to <http://www.rtog.org/ResearchAssociates/AdverseEventReporting/RTOGEORTCLateRadiationMorbidityScoringSchema.aspx>

|  | Grade 0 | Grade 1 | Grade 2 | Grade 3 | Grade 4 | Grade 5 |
| --- | --- | --- | --- | --- | --- | --- |
| Lung | None | Asymptomatic or mild symptoms (dry cough) Slight radiographic appearances | Moderate symptomatic fibrosis or pneumonitis (severe cough) Low grade fever Patchy radiographic appearances | Severe symptomatic fibrosis or pneumonitis Dense radiographic changes | Severe respiratory insufficiency/ Continuous O_2_/ Assisted ventilation | Death |
| Heart | None | Asymptomatic or mild symptoms Transient T wave inversion & ST changes Sinus tachycardia >110 (at rest) | Moderate angina on effort Mild pericarditis Normal heart size Persistent abnormal T wave and ST changes Low ORS | Severe angina Pericardial effusion Constrictive pericarditis Moderate heart failure Cardiac enlargement EKG abnormalities | Tamponade/ Severe heart failure/ Severe constrictive pericarditis | Death |
| Oesophagus | None | Mild fibrosis Slight difficulty in swallowing solids No pain on swallowing | Unable to take solid food normally Swallowing semi-solid food Dilatation may be indicated | Severe fibrosis Able to swallow only liquids May have pain on swallowing Dilation required | Necrosis/ Perforation Fistula | Death |

# Appendix D: Mellow Dysphagia score

0 = able to eat normal diet / no dysphagia.

1 = able to swallow some solid foods

2 = able to swallow only semi solid foods

3 = able to swallow liquids only

4 = unable to swallow anything / total dysphagia

# Appendix E: Protocol Amendment history

| **Amendment No.** | **Protocol Version No.** | **Date issued** | **Author(s) of changes** | **Details of Changes made** |
| --- | --- | --- | --- | --- |
| 001 | V3.0 | 13Dec2016 | Maria Hawkins, Claire Hamill, Stephanie Levy. | Protocol V1.0 28Jun2016 REC approved 19Aug2016.  The MHRA and OCTO chemotherapy, pharmacy advisory service (CPAS) initial reviews required changes to protocol V1.0 which updated the protocol to V2.0 10Aug2016. Version 2.0 received MHRA approval 19Aug2016. This was then submitted to the REC as a substantial amendment with additional minor amendments updating the document to V3.0 13Dec2016. |
| 002 | V4.0 | 14May2018 | Stephanie Levy | IMP name change from VX-970 to M6620 (Berzosertib). Change in IMP manufacturer. Additional secondary endpoint to Stage A2. Change to definition of end of study. DLT specification updated. Additional minor clarifications or corrections. |
| 004 | V5.0 | 26Oct2020 | Maria Hawkins, Jane Holmes, Alex Ooms, Evan Ridgeon, Usha Wahengbam, Steph Levy | Significant design changes to Stage B, including confirmed dose, treatment levels, follow up duration, and escalation/recruitment process. Change in end of trial timepoint, final report, pregnancy follow up. Flexibility in recruitment gaps. PK sampling removed. Eligibility criteria updates. Remove use of carboplatin. Stage A1 archival biopsy added. RSI update. Updates per IB & SmPCs. DLT added, DLT amended & clarity on treatment of patients with DLTs. Hb values corrected. Flexibility in assessments & treatment. RTTQA update. Administrative changes. |

1. The Medicines for Human Use (Clinical Trials) Regulations (S.I. 2004/1031) and any subsequent amendments to it. [↑](#footnote-ref-2)
