# Supplementary material for: CHARIOT: a phase I study of berzosertib with chemoradiotherapy in oesophageal and other solid cancers using time to event continual reassessment method
Source: Br J Cancer. 2023 Dec 21;130(3):467–75. doi: 10.1038/s41416-023-02542-1 (PMC10844302; doi:10.1038/s41416-023-02542-1)
Supplement: Supplementary file 3 — Supplementary information - CHARIOT Radiotherapy Quality Assurance Summary [file 41416_2023_2542_MOESM3_ESM.pdf]

CHARIOT Radiotherapy Quality Assurance Summary (Stage A1)

Patients recruited to Stage A1 of CHARIOT were prescribed a radiotherapy dose of 35Gy in 15 fractions. Approval from the Radiotherapy Trials Quality Assurance (RTTQA) group was required for centres to recruit to Stage A1 of CHARIOT. This report summarises the radiotherapy quality assurance programme for patients within the trial.

Pre-Trial Quality Assurance

RTTQA approval for participation in CHARIOT was streamlined based on the completion of pre-trial benchmarking exercises for SCOPE2 or NeoSCOPE, to reduce the barriers to centres opening to the trial as far as possible. In all, five radiotherapy centres were given RTTQA approval through the streamlining process.

The use of 4DCT was permitted for use within CHARIOT, with streamlined approval based on the completion of the corresponding pre-trial benchmarking exercises for SCOPE2 or NeoSCOPE.

Table 1 summarises the QA status of each centre participating in Stage A1 of CHARIOT.

Table 1: Summary of Pre-Trial Quality Assurance Approvals for Centres Participating in CHARIOT Stage A1

**Centre Name**	**SCOPE2 Centre #**	**Lower-third Outlining Status**	**Lower 3rd 4DCT Outlining Status**	**Planning Exercise Status**
Oxford, Churchill	121	Approved (NeoSCOPE)	Approved (NeoSCOPE)	Approved (SCOPE2)
Glasgow, Beatson	067	Approved (SCOPE2)	Approved (SCOPE2)	Approved (SCOPE2)
Leeds, St James	182	Approved (NeoSCOPE)	Approved (SCOPE2)	Approved (SCOPE2)
Cardiff, Velindre	001	Approved (SCOPE2)	Approved (SCOPE2)	Approved (SCOPE2)
Manchester, Christie	006	Approved (SCOPE2)	Approved (SCOPE2)	Approved (SCOPE2)

On-Trial Quality Assurance

The radiotherapy plan data for all patients recruited to Stage A1 of CHARIOT were subject to timely retrospective review. RTTQA reviewers assessed the delineation of target volumes and organs at risk, as well as the final treatment plan, with feedback given to the recruiting centre in advance of the 10th treatment fraction.

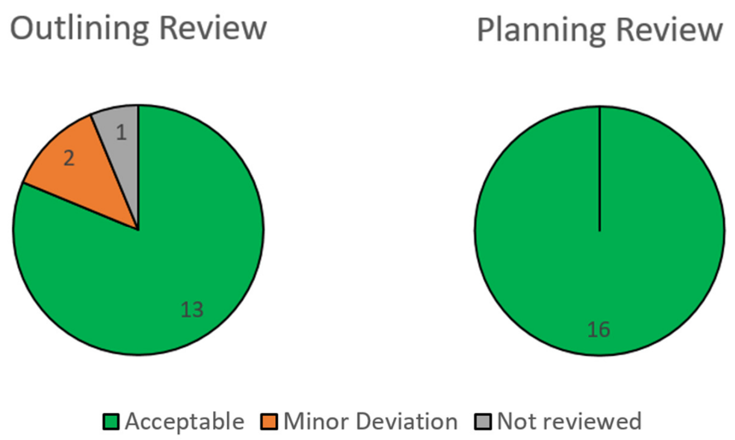

Figure 1: QA Outcome for outlining and planning review for on-trial patients in Stage A1

Figure 1 summarises the outcomes of the on-trial reviews for the 16 patients recruited to Stage A1. Minor deviations from protocol were recorded for 2 cases. Both deviations were related to the incorrect growth of margins for target volumes.

One case was not reviewed within the agreed timescales due to the availability of clinical staff to review the case. Arrangements were made for cross-cover to reduce the likelihood of this reoccurring.

Radiotherapy treatment plan review found all 16 cases met the dose objectives for the trial, and displayed an acceptable dose distribution. Centres used the CHARIOT Plan Assessment Form (PAF) to submit dose-volume histogram (DVH) data for each patient recruited to Stage A1 of the trial. The reported DVH statistics were independently verified as part of the plan review process. Table 2 summarises the data submitted for all 16 patients recruited to Stage A1 of CHARIOT.

Conclusion

The CHARIOT RTTQA programme verified that the delineation of target volumes and organs at risk, as well as the optimisation of the radiotherapy treatment plan met the specifications laid out in the CHARIOT trial protocol. Deviations from protocol were recorded in 12.5% of on-trial cases, emphasising the need for ongoing quality assurance checks in radiotherapy clinical trials.

Table 2: DVH statistics reported through plan assessment forms for each on-trial patient

Patient number	GTV Volume (cm^3)	PTV Volume (cm^3)	PTV D99% (Gy)	PTV V95% (%)	External D1.8cc (%)	SpinalCord_PRV D0.1cc (Gy)	Heart Dmean (Gy)	Heart V28Gy (%)	Lungs Dmean (Gy)	Lungs V18Gy (%)	Liver Dmean (Gy)	Liver V28Gy (%)	Kidney_L V18Gy (%)	Kidney_R V18Gy (%)
CH-A1-101	107.7	642.7	33.5	99.5	103.1	30.8	18.3	13.5	10.6	11.1	8.0	13.5	0.0	0.0
CH-A1-102	23.4	257.8	33.7	100.0	103.3	26.8	13.6	11.8	3.0	4.7	3.8	2.6	0.0	0.0
CH-A1-103	75.0	548.3	33.8	99.9	103.3	27.0	17.8	10.8	8.0	8.2	12.8	7.1	Not outlined	0.0
CH-A1-104	109.1	563.9	33.9	99.9	104.8	32.3	20.0	18.8	8.6	12.2	0.9	0.0	0.0	0.0
CH-A1-105	12.4	222.9	34.0	99.5	105.7	34.5	0.1	0.0	3.1	4.5	0.0	0.0	0.0	0.0
CH-A1-106	56.5	417.2	34.8	100.0	105.0	27.4	16.3	12.1	9.0	12.3	8.3	2.3	0.0	0.0
CH-A1-107	51.3	377.0	33.3	99.1	104.9	24.3	9.6	7.1	8.4	6.3	0.6	0.0	Not outlined	Not outlined
CH-A1-108	63.1	482.8	31.8	91.7	104.9	32.9	5.2	5.6	8.7	11.7	Not outlined	Not outlined	Not outlined	Not outlined
CH-A1-109	283.7	912.7	33.6	99.7	103.8	27.7	12.4	11.0	3.9	2.2	12.4	4.8	0.0	0.0
CH-A1-110	65.3	408.8	32.3	95.6	104.1	30.6	6.2	2.7	8.9	10.3	0.4	0.0	Not outlined	Not outlined
CH-A1-111	39.7	375.2	33.4	99.5	104.3	23.5	15.9	10.2	6.5	7.5	5.8	1.4	0.0	0.0
CH-A1-112	74.8	462.1	33.5	99.6	104.6	24.1	13.4	5.4	8.9	7.2	8.4	1.7	Not outlined	Not outlined
CH-A1-113	124.4	690.7	34.1	100.0	106.3	24.5	15.9	7.9	8.1	11.4	7.1	1.7	0.0	0.0
CH-A1-114	43.5	396.1	33.4	99.2	104.4	30.6	18.5	22.5	8.4	6.7	Not outlined	Not outlined	Not outlined	Not outlined
CH-A1-115	30.2	350.0	34.1	98.7	101.8	27.4	14.4	7.5	8.9	7.1	6.3	1.5	Not outlined	Not outlined
CH-A1-116	48.1	352.1	33.3	99.2	103.8	18.0	9.8	5.4	4.9	5.1	11.0	3.5	Not outlined	Not outlined
